# PAD2 Deficiency Reveals Sex-Divergent Colonic Homeostasis, β-Catenin Remodeling, and Antibiotic-Sensitive Phenotypes

**DOI:** 10.3390/biom16101415

**Published:** 2026-09-30

**Authors:** Zachary D. Dawson, Tamriage Martin, Eunhye Ji, Kyung Eun Lee, Xi Chen, Xinyu Zhou, Samantha St. Jean, Yongqing Li, Jianjie Ma

**Affiliations:** 1 Department of Surgery, Division of Surgical Sciences, University of Virginia School of Medicine, Charlottesville, VA 22908, USA; nwm4pp@virginia.edu (Z.D.D.); tam9wx@uvahealth.org (T.M.);; 2 Experimental Pathology, Biomedical Sciences Graduate Program, University of Virginia, Charlottesville, VA 22908, USA; 3 Comparative Pathology and Veterinary Diagnostics, University of Virginia, Charlottesville, VA 22908, USA; 4 Department of Surgery, University of Michigan Health System, Ann Arbor, MI 48109, USA

**Keywords:** citrullination, inflammatory bowel disease, intestinal microbiome, mucosal healing

## Abstract

Peptidylarginine deiminase 2 (PAD2) catalyzes protein citrullination and is implicated in inflammatory bowel disease and colorectal cancer. However, its role in regulating the intestinal epithelium and the influence of biological sex and the microbiome remain poorly defined. This study investigated the relationships among PAD2 deficiency, sex-divergent epithelial states, β-catenin regulation, and the intestinal microbiome. Global PAD2 knockout and wild-type littermate mice of both sexes were characterized under basal conditions and following dextran sodium sulfate-induced colitis. Intestinal epithelial phenotypes were assessed by histology, immunofluorescence, immunoblotting, quantitative RT-PCR, and immunoprecipitation. Gut microbial communities were evaluated by shotgun metagenomics, while broad-spectrum antibiotic treatment, fecal protein immunoblotting, and recombinant human PAD2 exposure in vitro were used to examine relationships among antibiotic-sensitive factors, β-catenin regulation, and epithelial phenotypes. PAD2 deficiency was associated with distinct epithelial states in male and female colons before injury. Male KO mice displayed increased β-catenin phosphorylation and increased susceptibility to DSS colitis, whereas female KO mice demonstrated increased total β-catenin abundance and preservation of structural epithelial features following injury. Female KO microbiota exhibited genotype-associated differences in microbial community composition by exploratory Bray-Curtis PERMANOVA. Broad-spectrum antibiotic treatment altered the female epithelial phenotype, reduced active β-catenin, and progressively depleted luminal PAD2-immunoreactive proteins. Exogenous recombinant human PAD2 increased epithelial protein citrullination and altered active β-catenin abundance in HT29 cells. Collectively, these findings demonstrate both sex-specific and sex-divergent colonic phenotypes while supporting a framework connecting PAD2 biology, epithelial architecture, and the microbial environment during intestinal maintenance and injury.

## 1. Introduction

Inflammatory bowel disease (IBD) is sustained by dysregulated immune activity, epithelial barrier dysfunction, and impaired mucosal repair [1,2]. Although modern therapies can suppress inflammatory signaling, durable remission relies on restoration of a functional epithelial barrier. Intestinal repair, therefore, requires coordinated control of crypt-cell proliferation, lineage allocation, mucus production, and restitution after injury [3,4,5]. Identifying molecular pathways that integrate these processes remains central to the development of therapies that promote mucosal healing rather than inflammatory control alone.

Canonical Wnt/β-catenin signaling is a principal regulator of intestinal stem-cell maintenance, crypt regeneration, and epithelial lineage organization [6,7,8]. In the absence of Wnt stimulation, sequential phosphorylation of β-catenin by casein kinase 1 (CK1) and glycogen synthase kinase 3β (GSK3β) targets β-catenin for ubiquitination and proteasomal degradation [9,10]. Conversely, stabilization of β-catenin supports nuclear transcriptional programs that sustain epithelial renewal. Insufficient β-catenin signaling can impair epithelial repair, whereas excessive signaling can promote neoplasia. Therefore β-catenin abundance, localization, and transcriptional activity must be tightly controlled [11,12]. Phosphorylation-dependent regulation is well defined; substantially less is understood about how alternative post-translational modifications, such as citrullination, shape β-catenin signaling cascades during intestinal homeostasis and injury.

Citrullination is an irreversible, calcium-dependent conversion of protein arginine residues to citrulline catalyzed by the peptidylarginine deiminase (PAD) family [13]. PAD2 is broadly expressed and has been detected in the colonic mucosa [14,15]. Prior findings from patient tissues and biochemical studies identified β-catenin as a PAD2 substrate and showed that PAD2-dependent citrullination can alter β-catenin stability and Wnt signaling [16,17]. Altered PAD expression and citrullinated proteins have also been reported in IBD and colorectal cancer [14,15,18]. Although increasing evidence indicates that epithelial responses in experimental colitis vary by sex, the direction and magnitude of these effects across experimental models remain incompletely understood [19,20]. Despite these observations, studies of intestinal PAD biology have largely emphasized inflammatory-cell response, extracellular-trap formation, and histone citrullination [21,22,23,24,25]. Whether PAD2 contributes to epithelial organization under basal conditions, and whether its regulation of the colonic epithelium differs between males and females, remain insufficiently defined.

The intestinal microbiota adds an additional layer of regulation by shaping mucus production, epithelial metabolism, stem-cell activity, and recovery after injury [26,27,28]. Microbial community structure and metabolism are influenced by host genome, biological sex, and environmental factors [29,30,31]. Conversely, microbial metabolites and proteins can influence host physiology [32]. Catalytically active PAD or PAD-like enzymes have been identified in select bacterial species, raising the possibility that extracellular or microbial PAD-like peptides participate in host–microbial communication [33,34,35,36]. The identity, abundance, and functional relevance of such mechanisms in the colon remain largely unknown.

In this study, we investigated whether PAD2 deficiency is associated with sex differences in the colonic epithelium and susceptibility to DSS-induced injury. We further examined whether these phenotypes are accompanied by distinct β-catenin states and intestinal microbial composition. We then assessed whether broad-spectrum antibiotic treatment alters PAD2-associated epithelial and β-catenin phenotypes. Together, these experiments were designed to lay a foundation for understanding the relationships among PAD2, biological sex, β-catenin, and the intestinal microbiome during colonic homeostasis and injury.

## 2. Materials and Methods

### 2.1. Animals

PAD2 knockout (*PAD2*^−/−^) mice were generously provided by Yongqing Li (University of Michigan) and maintained under specific pathogen-free conditions at the University of Virginia on a 12-h light/dark cycle (24–26 °C) with ad libitum access to irradiated chow (Teklad, Madison, WI, USA; LM-485) and water. Mice were backcrossed for six generations onto a C57BL/6J background (Jackson Lab, Sacramento, CA, USA). Genome-wide single nucleotide polymorphism analysis (MiniMUGA; TransnetYX, Cordova, TN, USA) confirmed a >98% C57BL/6J genetic background. Residual non-C57BL/6J markers were detected, including a heterozygous interval on chromosome 4 adjacent to the targeted *Padi2* locus. MiniMUGA background assignments for this interval were not definitive. The female sample was classified as C57BL/6J × LP/J by the genome-background algorithm, whereas diagnostic-marker analysis identified FVB/NJ as an alternative background assignment. The residual interval is therefore described as non-C57BL/6J rather than assigned to a specific donor strain (Figure S1B).

To reduce maternal microbiome, epigenetic, and environmental confounders, experimental cohorts were produced using heterozygous (*PAD2^+/−^*) breeding pairs (Figure S1A), generating wild-type (WT) and PAD2 knockout (KO) littermates within the same breeding cage. Offspring were genotyped at weaning using previously described primers (Table A2), separated by sex and genotype, and studied between 12 and 16 weeks of age. Independent biological cohorts were generated over approximately two years of continuous breeding. Estrous cycle stage was not monitored in female mice, and gonadal weights or circulating sex hormone concentrations were not assessed in either sex.

Because individual litters did not consistently yield equal numbers of male and female mice of each genotype, group sizes varied among experiments according to the availability of appropriately matched littermates. For individual downstream assays, sample numbers may be unequal when samples were unavailable or could not be reliably analyzed due to assay-specific technical limitations.

### 2.2. Basal Phenotyping and DSS Colitis

Following genotyping, WT and KO littermates were housed according to sex and genotype. Mice received heparin (100 μL, intraperitoneal) 30 min before tissue collection. Blood was obtained from the inferior vena cava under isoflurane anesthesia, followed by whole-body perfusion with phosphate-buffered saline (PBS) and cervical euthanasia. Colons were excised, cleared of mesenteric fat, weighed and measured with the cecum intact. Animals used for intestinal permeability assays were excluded from subsequent biochemical analyses.

For experimental colitis, mice were transitioned from automated water systems to drinking bottles for seven days before treatment. Survival studies were performed using 3% (*w*/*v*) dextran sulfate sodium (DSS; 36–50 kDa; MP Biomedicals, Irvine, CA, USA) administered ad libitum until humane endpoint. Humane endpoint was defined according to predefined criteria, including ≥20% body-weight loss. Terminal tissues from the survival cohort were collected at euthanasia; consequently, the duration of DSS exposure at tissue collection varied among animals. Separate cohorts were euthanized after seven days of 3% DSS exposure for histologic and biochemical analyses. Antibiotic-dependent colitis experiments were performed using 2.5% DSS following microbiome disruption as described below.

### 2.3. Disease Activity Index

Body weight, stool consistency, fecal blood, and hematochezia were recorded daily. DAI was calculated using predefined scoring criteria [37].

### 2.4. Intestinal Permeability

Mice were fasted for 16 h then given oral gavage of 3–5-kDa FITC-dextran (Sigma-Aldrich, St. Louis, MO, USA) at 4.4 μL/g body weight from a freshly prepared 100 mg/mL stock in PBS. Blood was collected 4 h later. Serum fluorescence was measured at excitation 485 nm and emission 528 nm against FITC-dextran standards.

### 2.5. Histology and Morphometry

Colons were opened longitudinally, gently flushed with PBS, and prepared as Swiss rolls with the proximal colon positioned centrally. Tissues were fixed overnight in 4% paraformaldehyde at 4 °C, transferred to 70% ethanol, further processed using the Excelsior AS automated tissue processor (Epredia, Kalamazoo, MI, USA), and paraffin embedded. Tissues were sectioned at 8 μm and mounted on charged glass slides. Sections from comparable depths of each Swiss roll were used for all histologic analyses.

Hematoxylin and eosin (H&E) staining was performed using a commercial staining kit (Abcam; ab245880) according to the manufacturer’s instructions with minor timing modifications. Whole-slide images were acquired using a Leica DMi8 microscope (Wetzlar, Germany) with a 10× objective and stitched using LAS X software version 4.6.1. Images were analyzed using Fiji (ImageJ) version 2.16.0/1.54p. Crypt depth was measured in well-oriented proximal colonic crypts as the distance from the crypt base to the luminal surface [38]. Basal crypt depth analysis was done using a fixed sampling scheme of exactly 130 well-oriented proximal colonic crypts measured per mouse. Measurements were averaged within each mouse, and the resulting per-mouse mean was used as the biological replicate for statistical analysis.

Unless otherwise specified, quantitative morphometric and image analyses were not performed blinded to genotype or sex. In select immunofluorescence analyses, measurements were independently verified by a second investigator; however, independent evaluation was not performed for all datasets. Samples or images that could not be reliably quantified because of technical limitations, including inadequate crypt orientation for morphometric measurements or insufficient image quality to reliably distinguish fluorescence signal from background, were not included in the corresponding quantitative analysis.

### 2.6. Alcian Blue Staining and Goblet Cell Analysis

Acidic mucins were visualized with Alcian Blue, pH 2.5 (Vector Laboratories, Newark, CA, USA; H-3501) according to the manufacturer’s protocol. Goblet cells were quantified in longitudinally oriented proximal crypts from well-preserved tissue sections at 200× magnification. Using a fixed sampling scheme, exactly 103 crypts were assessed per mouse [39]. Per-mouse means were used as the biological replicate for statistical analysis.

For Alcian Blue-positive area quantification, a common threshold was established using representative intact tissue from multiple mice at baseline to distinguish positive staining from background. Once established, this threshold was fixed and applied consistently across all images within the corresponding analysis. Alcian Blue-positive area was normalized to the total tissue area.

### 2.7. BrdU Labeling

Mice received 1.5 mg BrdU (BD, San Jose, CA, USA; 550891) intraperitoneally. Colonic tissue was harvested 24 h later as described above. BrdU-positive epithelial nuclei were quantified via immunofluorescence, and the BrdU labeling index (%) was calculated as the number of BrdU-positive nuclei divided by the total number of nuclei. One Swiss roll section per mouse was analyzed, with each mouse considered the biological replicate.

### 2.8. Immunofluorescence and Microscopy

FFPE sections were deparaffinized, subjected to citrate-based heat-induced antigen retrieval (BioSB, Santa Barbara, CA, USA; 0020), permeabilized and blocked in 2% goat serum (SouthernBiotech, Birmingham, AL, USA) and 0.01% Triton X-100 (Sigma-Aldrich) buffer. Mouse-on-Mouse reagent (Vector Laboratories) was used where appropriate. Sections were incubated with primary antibodies [1:100] overnight at 4 °C, followed by Alexa Fluor-conjugated secondary antibodies [1:1000] (Table A1) and mounted with Fluoromount+DAPI (SouthernBiotech, Birmingham, AL, USA). Images were acquired on a Leica Stellaris 5 confocal microscope (Wetzlar, Germany) using a 20× or 63× objective, with acquisition settings held constant within each experiment. Only linear brightness and contrast adjustments were applied to images for visualization and were performed equally across comparison groups using FIJI.

### 2.9. Histopathology

Histopathologic scoring was performed by a board-certified veterinary pathologist (SSJ) blinded to genotype and treatment. Parameters of colonic injury and inflammation were evaluated using a semiquantitative scoring system ranging from 0 to 4, with scores defined as normal (0), minimal (1), mild (2), moderate (3), and marked (4). The composite score incorporated inflammatory-cell infiltration, mucosal erosion/ulceration, epithelial hyperplasia, submucosal edema, crypt abscesses, fibrin deposition, and anatomic distribution.

### 2.10. Shotgun Metagenomic Sequencing and Functional Analysis

Fresh fecal pellets were collected from 10–12-week WT and PAD2 KO littermate mice across independent breeding cohorts into nucleic acid stabilization buffer (TransnetYX, Cordova, TN, USA). Within each sex, WT and KO mice were analyzed as littermate pairs derived from the same breeding cohort. Each pair originated from a different breeder, such that no breeder contributed more than one sequenced pair. Following genotyping and separation at 4–6 weeks, mice were housed according to sex and genotype, with the number of animals per cage varying according to the available genotype distribution. Male and female samples were analyzed separately due to limited overlap in breeding cohort timing. Barcoded samples were submitted to TransnetYX for sample extraction and whole-genome shotgun metagenomic sequencing, performed using the Illumina NextSeq 2000 (San Diego, CA, USA) at a depth of 2 million 2 × 150-bp read pairs. Taxonomic assignment was performed against the One Codex reference genome database. Functional profiling was performed using the One Codex Functional Analysis workflow, which implements a modified HUMAnN3 pipeline for gene-family annotation and MetaCyc pathway reconstruction [40,41,42]. Microbial community composition was evaluated at the phylum and genus levels. Alpha diversity was assessed at the phylum level using the Shannon and Simpson diversity indices. Beta diversity was evaluated using Bray–Curtis dissimilarity and weighted UniFrac distances, with genotype-associated differences in community composition assessed by permutational multivariate analysis of variance (PERMANOVA; 999 permutations). PERMANOVA was performed using genotype as the grouping variable with unrestricted permutations across individual animals. Although WT and KO animals were selected as littermate pairs from independent breeding cohorts, permutations were not restricted by breeding cohort because each contained only one sequenced animal per genotype, resulting in a highly limited within-cohort permutation space. Accordingly, PERMANOVA was considered exploratory. Homogeneity of multivariate dispersion between genotypes was evaluated by PERMDISP using Bray–Curtis dissimilarities. Predicted microbial metabolic pathways were evaluated using MetaCyc pathway abundance profiles generated by the One Codex Functional Analysis workflow. Pathway abundance data were obtained through the One Codex Custom Plots interface, which permits visualization and export of up to 50 functions with the highest average values for the selected annotation and metric. Accordingly, the 50 MetaCyc pathways evaluated here represented the platform-defined set of highest-average-abundance pathways available through this interface and were not manually selected based on genotype or statistical significance. Within each sex, pathway abundances were compared between matched WT and KO littermates using two-sided Wilcoxon matched-pairs signed-rank tests. *p* values were adjusted across the 50 evaluated pathways using the Benjamini–Hochberg false discovery rate (FDR) procedure. Shotgun metagenomic sequence data have been deposited in NCBI under accession number PRJNA1508927.

### 2.11. Microbiome Disruption

Mice received a broad-spectrum antibiotic cocktail in drinking water for 5 days, 2 weeks, or 4 weeks, as indicated. Antibiotic water was replaced weekly throughout treatment. The cocktail contained neomycin (100 μg/mL), vancomycin (50 μg/mL), imipenem (50 μg/mL), metronidazole (100 μg/mL), streptomycin (50 μg/mL), enrofloxacin (0.5 mg/mL), penicillin (100 U/mL), and 1% sucrose to improve palatability [43]. For ABX + DSS experiments, the corresponding DSS-only control animals also received 1% sucrose in their drinking water. For microbiome-disrupted DSS experiments, two independent antibiotic-treatment paradigms were used. In the prolonged ABX experiment, male and female WT and PAD2 KO mice received ABX for 4 weeks before 2.5% DSS administration and continued to receive ABX throughout DSS exposure. Male and female mice represented contemporaneous arms of the same experiment. In the short-term ABX experiment, female WT and PAD2 KO mice received ABX for 5 days before assignment to ABX alone or ABX plus 2.5% DSS, with ABX continued throughout the experimental period to avoid microbial rebound. Fecal samples were collected longitudinally for immunoblotting at the indicated time points.

### 2.12. Fecal Protein Extraction

Proteins were extracted in freshly made SDS/urea lysis buffer containing protease and phosphatase inhibitors by mechanical homogenization and sonication [44]. Insoluble material was removed by refrigerated centrifugation, and protein concentrations were determined using the Bradford assay (BioRad, Hercules, CA, USA; 5000006). Equal amounts of sample proteins were mixed with reducing Laemmli sample buffer, boiled at 95 °C for 5 min, separated by SDS-PAGE, and transferred for immunoblot analysis as described below. Total protein staining with Ponceau S (Sigma-Aldrich, St. Louis, MO, USA) served as the loading control for all fecal immunoblots. Throughout the manuscript, anti-PAD2-reactive fecal bands are described as PAD2-immunoreactive proteins, reflecting antibody recognition without assignment of molecular identity.

### 2.13. Immunoblotting

Proximal colon tissue was homogenized in ice-cold RIPA buffer (Thermo Scientific, Waltham, MA, USA; 89900) containing protease and phosphatase inhibitors (Sigma-Aldrich). Lysates were clarified by refrigerated centrifugation and protein concentrations were determined using the Bradford assay. Equal protein quantities (typically 30 μg) were resolved by SDS-PAGE, transferred to PVDF (Bio-Rad), blocked in 5% milk in TBST for one hour at room temperature (Tris-buffered saline with 0.1% Tween-20), and incubated with primary antibodies [1:1000] (Table A1) overnight at 4 °C. After washing, HRP-conjugated secondary antibodies [1:3000] were incubated with membranes for one hour at room temperature, washed, and chemiluminescent substrates were used for detection. Total protein staining was used where appropriate. Citrullinated proteins were detected with the Anti-Modified Citrulline Detection Kit (Millipore, Burlington, MA, USA; 17-347). Uncropped blots and molecular-weight markers are presented as supplemental source data. For original immunoblot source images, lanes marked with an ‘X’ indicate samples that were not included in the corresponding displayed figure panel, in accordance with the journal’s original-image requirements. All immunoblot strips presented in the figures are contiguous crops from their respective source images. No intervening lanes were digitally removed, and no non-adjacent lanes were digitally juxtaposed or rearranged. For the revised analysis of Figure 5A,B, β-catenin densitometry was re-derived from matched WT and PAD2 KO samples analyzed on the same immunoblots. Total β-catenin and phosphorylated β-catenin were normalized to GAPDH, and phosphorylated β-catenin was additionally normalized to total β-catenin. Because WT and KO samples were matched within immunoblots, comparisons were analyzed using two-sided paired *t*-tests rather than the unpaired Welch’s *t*-tests used in the original submission. The p-β-catenin/total β-catenin ratio was log-transformed before paired analysis; β-catenin/GAPDH and p-β-catenin/GAPDH were analyzed without transformation. All band intensities were quantified using Fiji (ImageJ) and normalized to GAPDH, total protein staining, or as indicated in the corresponding figure legends.

### 2.14. HT29 Cell Culture and rhPAD2 Treatment

HT29 human colorectal epithelial cells (ATCC; HTB-38) were maintained in McCoy’s 5A medium (Cytiva, Marlborough, MA, USA) supplemented with 10% fetal bovine serum (Thermo Scientific, Waltham, MA, USA) and 1% penicillin–streptomycin (Thermo Scientific, Waltham, MA, USA) at 37 °C in a humidified incubator containing 5% CO_2_. Medium was replaced every 2–3 days for passaging.

For recombinant human PAD2 experiments (rhPAD2, Cayman; 10785), cells were seeded in six-well plates and allowed to adhere and reach >85% confluence. Preliminary experiments evaluated rhPAD2 response in both DMEM and McCoy’s 5A medium. A comparable response was not observed under the tested DMEM condition. Therefore, subsequent quantitative experiments were performed in McCoy’s 5A, the recommended culture medium for HT29 cells. Medium was replaced with fresh McCoy’s 5A containing rhPAD2 (0.5 μg/mL), 2 mM CaCl_2_, 2 mM EGTA (Bioworld, Dublin, OH, USA), or the indicated combinations. Following 24 h of treatment, conditioned medium was collected and clarified by centrifugation, while cell lysates were prepared in RIPA buffer containing protease and phosphatase inhibitors after 3 washes with PBS. Conditioned media and cell lysates were analyzed by immunoblotting as described above.

For immunofluorescence, treated cells were cultured on chamber slides (Ibidi, Fitchburg, WI, USA; 80806), fixed in 4% paraformaldehyde for 12 h, permeabilized and blocked in PBS containing 2% goat serum (SouthernBiotech, Birmingham, AL, USA) and 0.01% Triton X-100 (Sigma-Aldrich) buffer for one hour. Samples were incubated overnight with primary antibodies [1:100], washed, and visualized using Alexa Fluor-conjugated secondary antibodies [1:1000] and DAPI. Images were acquired using identical microscope settings within each experiment.

### 2.15. Quantitative PCR

Total RNA was isolated from proximal colon using TRIzol (Invitrogen, Carlsbad, CA, USA) reagent according to the manufacturer’s instructions. RNA concentration and purity were determined spectrophotometrically (NanoDrop One, Wilmington, DE, USA), and equal amounts of RNA were reverse transcribed using the RevertAid H Minus First Strand cDNA synthesis kit (Thermo Fisher Scientific; FERK1632). Gene expression was quantified using PowerTrack SYBR Green Master Mix (Thermo Fisher Scientific; A46111) on a QuantStudio5 real-time PCR system. Ribosomal protein lateral stalk subunit P0 (*Rplp0*) served as the endogenous reference gene. Primer sequences are provided in Table A2. Relative gene expression is presented using the 2^−ΔΔCt^ method for visualization. Statistical analyses were performed on ΔCt values to satisfy assumptions of normality and variance. For genes analyzed across multiple qPCR runs, genotype and qPCR run were incorporated as fixed effects in the statistical model, and genotype × run interactions were evaluated where appropriate. Fold-change values are presented for visualization only.

### 2.16. Immunoprecipitation

Equal amounts of whole colon protein lysates (750 µg) were pre-cleared with unbound Protein A/G Dynabeads (Fisher Scientific) and incubated for 4 h at 4 °C with anti-β-catenin antibody or species-matched IgG control according to manufacturer instructions. Immune complexes were captured, washed extensively, and eluted in reducing sample buffer. Where indicated, calcium chloride was added during immunoprecipitation at the concentrations specified in the corresponding figure. Eluted proteins were analyzed by immunoblot for β-catenin, PAD2, or modified citrulline as indicated. Input lysates and IgG immunoprecipitation controls were included in each experiment.

### 2.17. Statistical Analysis

Individual mice were considered the biological unit for all in vivo analyses. When multiple measurements were obtained from the same animal (e.g., crypt depth or goblet cell counts), measurements were averaged within each mouse before statistical analysis unless otherwise specified. Data presentation is specified in the corresponding figure legends, with individual biological replicates shown whenever feasible. Two-group comparisons were analyzed using two-sided Welch’s *t* tests unless otherwise indicated. Comparisons involving multiple independent variables were analyzed using one- or two-way analysis of variance (ANOVA) followed by Šídák-adjusted planned multiple comparisons. For endpoints in which male and female cohorts could be analyzed jointly, sex-by-genotype effects were evaluated using two-way ANOVA with genotype and sex as factors including a genotype × sex interaction term, followed by Šídák-adjusted planned multiple comparisons between WT and KO mice within each sex. For experiments in which male and female samples were generated in separate experimental runs and could not be directly normalized across sex, genotype × sex interactions were not tested, and comparisons were restricted to genotype effects within sex. Longitudinal body weight and disease activity index measurements were analyzed using mixed-effects models (restricted maximum likelihood; REML) with Geisser–Greenhouse correction for repeated measures. For formal evaluation of sex-by-genotype effects in longitudinal DAI, mean DAI across the common Day 0–10 observation period was calculated for each animal and analyzed by two-way ANOVA with sex and genotype as factors, including the genotype × sex interaction term. Survival was analyzed using the log-rank (Mantel–Cox) test.

Microbial alpha-diversity was evaluated using the Shannon and Simpson diversity indices. Relative taxonomic abundances were compared using two-sided Wilcoxon matched-pairs signed-rank tests. Beta-diversity was assessed by permutational multivariate analysis of variance (PERMANOVA) with 999 permutations using Bray–Curtis dissimilarity or weighted UniFrac distances, as indicated. MetaCyc pathway abundances were compared using two-sided Wilcoxon matched-pairs signed-rank tests within each sex, with Benjamini–Hochberg correction across 50 pathways. Exact sample sizes, statistical tests, and multiple-comparison procedures are provided in the corresponding figure legends. Statistical analyses were performed using GraphPad Prism 11 (GraphPad Software, version 11.1), and One Codex where appropriate. Statistical significance was defined as *p* < 0.05. All authors had access to the study data and have reviewed and approved the final manuscript.

## 3. Results

### 3.1. PAD2 Deficiency Is Associated with Sex-Divergent Remodeling of Basal Colonic Architecture

**Figure 1:**
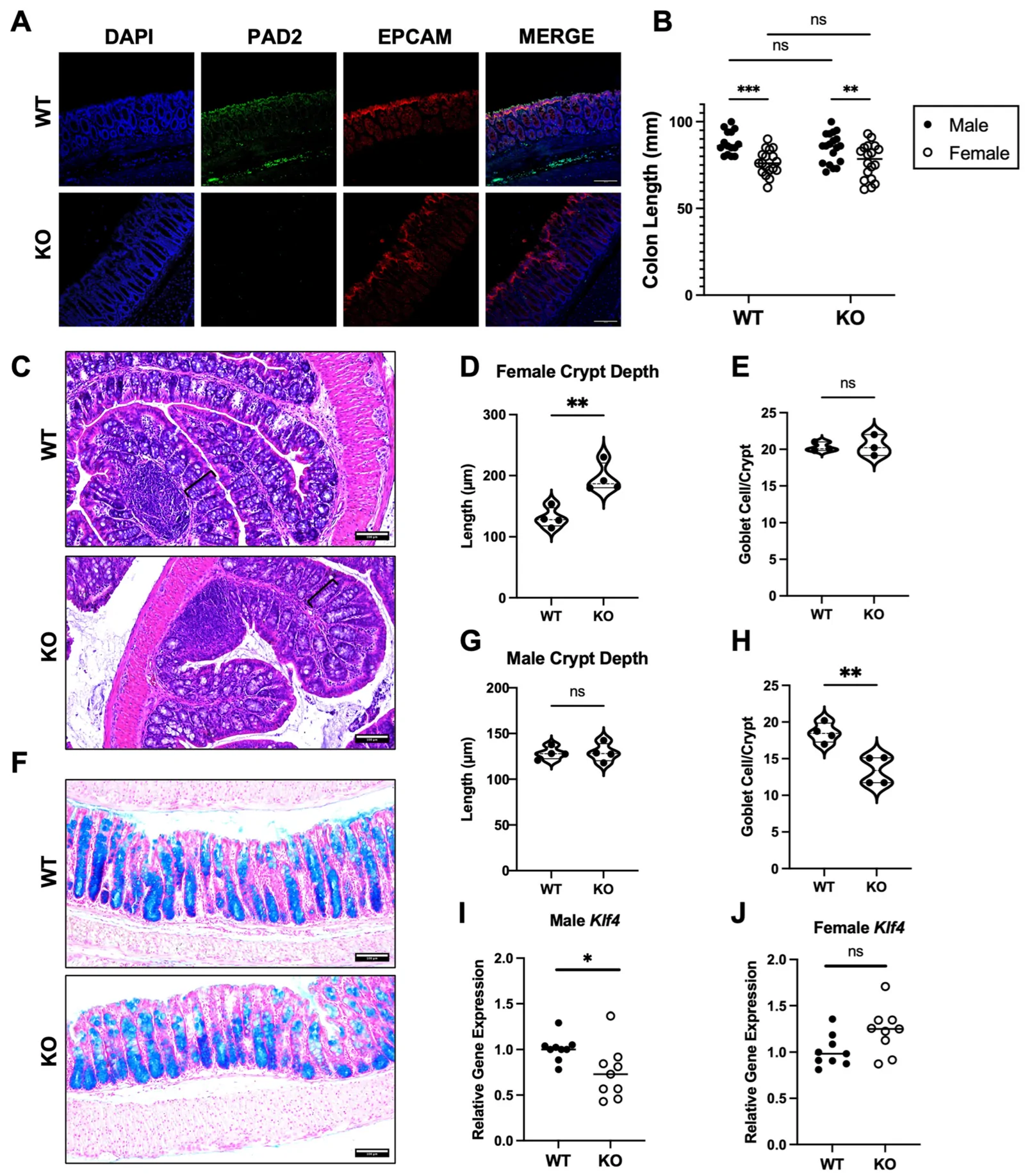
PAD2 is heterogeneously expressed in the murine colon and is associated with sex-divergent basal epithelial architecture. (**A**) Representative immunofluorescence images of distal colon sections showing PAD2 (green), EPCAM (red), nuclei (DAPI, blue), and merged images. Scale bar = 100 μm. (**B**) Baseline colon length measurements in male and female WT and PAD2 KO mice (n = 17 mice per group). (**C**) Representative hematoxylin and eosin (H&E)-stained proximal colon sections from female WT and PAD2 KO mice. Identically sized brackets indicate the mean WT crypt depth and are shown as a visual reference. Scale bar = 100 μm. (**D**,**E**) Violin plots showing crypt depth ((**D**); 130 crypts per mouse, n = 4 mice per genotype) and Alcian Blue-positive goblet cells per crypt ((**E**); 103 crypts per mouse, n = 3 mice per genotype) in female WT and PAD2 KO mice. (**F**) Representative Alcian Blue-stained distal colon sections from male WT and PAD2 KO mice, shown qualitatively. Quantitative goblet cell analysis was performed in proximal colonic crypts as described in the Methods. Scale bar = 100 μm. (**G**,**H**) Violin plots of crypt depth ((**G**); 130 crypts per mouse; n = 4 mice per genotype) and Alcian Blue-positive goblet cells per crypt ((**H**); 103 crypts per mouse; n = 4 mice per genotype) in male WT and PAD2 KO mice. For (**D**,**E**,**G**,**H**), overlaid points represent individual mouse means, which were used as the biological replicates for statistical analysis. (**I**,**J**) Relative *Klf4* mRNA expression in male ((**I**); *p* = 0.0291) and female ((**J**); *p* = 0.0788) colons (n = 9 mice per group). Data in (**I**,**J**) are presented as mean ± SEM with individual biological replicates shown. Sex-by-genotype effects were evaluated using two-way ANOVA with sex and genotype as factors including the sex × genotype interaction, followed by Šídák-adjusted planned comparisons between WT and PAD2 KO mice within each sex. Sex × genotype interactions were significant for crypt depth (*p* = 0.0106), goblet cell abundance (*p* = 0.0288), and *Klf4* expression (*p* = 0.0010). Baseline colon length was analyzed using two-way ANOVA with sex and genotype as factors. The genotype × sex interaction was not significant (*p* = 0.3440). Sex-specific endpoints were analyzed using two-sided Welch’s *t* tests. * *p* < 0.05; ** *p* < 0.01; *** *p* < 0.001. ns: not significant.

Because the epithelial function(s) of PAD2 in the basal intestine remain poorly defined, PAD2 distribution was first characterized in WT and PAD2 KO colons. PAD2 immunoreactivity was heterogeneous throughout the colon, with prominent staining in lamina propria immune cells and along the apical epithelial compartment, whereas a comparatively weak signal was detected within the lower crypt. PAD2 immunoreactivity was absent in KO tissue, supporting antibody specificity under these conditions (Figure 1A).

To examine whether constitutive PAD2 deficiency altered basal epithelial architecture, total colon length and proximal crypts were quantified in male and female littermate mice. Baseline colon length showed a significant main effect of sex (*p* < 0.0001), but no main effect of genotype (*p* = 0.5567) or genotype × sex interaction (*p* = 0.3440; Figure 1B). Female KO mice exhibited deeper crypts than WT controls, whereas no genotype-associated difference in crypt depth was detected in males (Figure 1C,D,G). Two-way ANOVA identified a significant genotype × sex interaction for crypt depth (*p* = 0.0106), indicating that the association between PAD2 genotype and crypt depth differed by sex.

Goblet cell differentiation was next evaluated by Alcian Blue staining and *Klf4* gene expression. Male KO mice displayed fewer goblet cells per crypt, together with reduced *Klf4* expression compared with WT controls (Figure 1F,H,I). In contrast, no genotype-associated difference in goblet cell abundance or *Klf4* expression was detected in female WT and KO mice (Figure 1E,J). Significant genotype × sex interactions were also detected for goblet cell abundance (*p* = 0.0288) and *Klf4* expression (*p* = 0.0010). Collectively, these findings demonstrate sex-divergent epithelial phenotypes associated with PAD2 deficiency, supported by distinct changes in crypt depth, goblet cell abundance, and Klf4 expression.

### 3.2. PAD2 Deficiency Is Associated with Increased DSS-Induced Injury in Males and Preservation of Selected Structural Endpoints in Females

**Figure 2:**
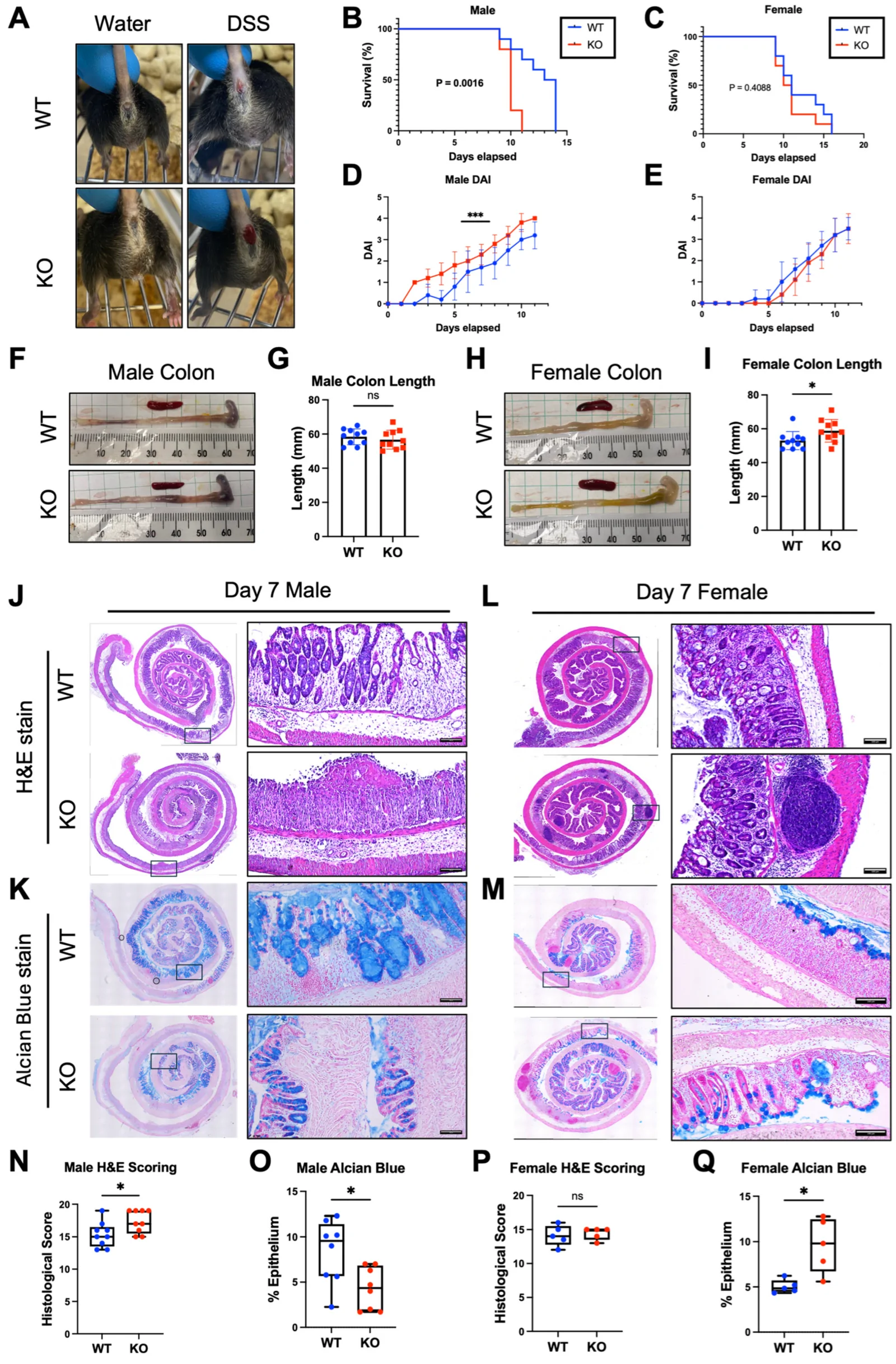
PAD2 deficiency worsens DSS colitis in males but preserves selected structural and mucin-associated endpoints in females. (**A**) Representative images of WT and PAD2 KO male mice before and on day 5 of 3% dextran sulfate sodium (DSS) exposure. (**B**,**C**) Kaplan–Meier survival curves of male (**B**) and female (**C**) WT and PAD2 KO mice during 3% DSS exposure (n = 10 mice per genotype). (**D**,**E**) Disease activity index (DAI) during 3% DSS exposure in male ((**D**); *p* = 0.0002) and female ((**E**); *p* = 0.1665) WT and PAD2 KO mice (n = 10 mice per genotype at study initiation). (**F**–**I**) Terminal colon measurements from the longitudinal 3% DSS survival cohort. Animals were euthanized and tissues collected at variable time points according to disease progression; individual DSS exposure durations at collection are reported in Table S1. (**F**,**G**) Representative gross colon images (**F**) and colon length measurements (**G**) from male WT and PAD2 KO mice (n = 10 mice per genotype). (**H**,**I**) Representative gross colon images (**H**) and colon length measurements (**I**) from female WT and PAD2 KO mice (n = 10 mice per genotype). (**J**–**Q**) Histologic and epithelial analyses from independent cohorts collected after a fixed 7-day exposure to 3% DSS. (**J,K**) Representative H&E-stained (**J**) and Alcian Blue-stained (**K**) whole Swiss-roll colon sections from male WT and PAD2 KO mice. Distal (H&E) and medial (Alcian Blue) insets are shown. (**L,M**) Representative H&E-stained (**L**) and Alcian Blue-stained (**M**) whole Swiss-roll colon sections from female WT and PAD2 KO mice. Medial (H&E) and distal (Alcian Blue) insets are shown. For (**J**–**M**), complete Swiss-roll sections are shown with magnified insets highlighting representative regional histopathologic features. Scale bars = 100 μm. (**N**,**P**) Histopathologic injury scores in male ((**N**); n = 9) and female ((**P**); n = 5) mice. (**O**,**Q**) Quantification of Alcian Blue-positive epithelial area in male ((**O**); n = 8) and female ((**Q**); n = 5) mice. Panels N-Q are presented as box-and-whisker plots showing the median, interquartile range, and minimum-to-maximum values, with individual biological replicates shown. Survival was analyzed using the log-rank (Mantel–Cox) test. Longitudinal DAI was analyzed using a mixed-effects model (REML) with Geisser–Greenhouse correction for repeated measures and Šídák-adjusted multiple comparisons. To formally evaluate sex-by-genotype effects, mean DAI across the common Day 0–10 observation period was calculated for each mouse, and mean DAI, colon length, histopathologic injury score, and Alcian Blue-positive epithelial area were analyzed by two-way ANOVA with sex and genotype as factors, including the sex × genotype interaction term. Significant interactions were detected for DAI (*p* < 0.0001), colon length (*p* = 0.0413), and Alcian Blue-positive epithelial area (*p* = 0.0006), whereas the interaction for histopathologic injury score was not significant (*p* = 0.2074). Sex-specific histopathologic injury scores and Alcian Blue-positive epithelial area were analyzed using two-sided Welch’s *t* test. * *p* < 0.05; *** *p* < 0.001. ns: not significant.

DSS disrupts the colonic epithelial barrier and therefore provides a well-established model for evaluating epithelial responses to injury [45,46]. To test whether the distinct basal epithelial phenotypes associated with constitutive PAD2 deficiency influenced susceptibility to epithelial injury, male and female WT and KO littermates were challenged with DSS across independent experimental cohorts. Male KO mice developed more severe disease than WT controls, exhibiting increased disease activity throughout DSS exposure, reduced survival, and increased intestinal permeability (Figure 2A,B,D; Figure S2A). Although no genotype-associated differences in terminal colon length were detected (Figure 2F,G), histopathologic injury was increased in male KO mice and was accompanied by reduced Alcian Blue-positive epithelial area in independent cohorts evaluated following a fixed 7-day DSS exposure (Figure 2J,K,N,O). Because colon length from the longitudinal survival cohort were obtained at terminal collection, DSS exposure duration varied among animals according to disease progression. These measurements therefore represent terminal structural outcomes rather than standardized fixed-time comparisons (Table S1).

In contrast, no genotype-associated differences were detected in disease activity, survival, intestinal permeability, or overall histopathologic injury in females (Figure 2C,E,L,P; Figure S2B). However, KO females retained significantly longer colons and greater Alcian Blue-positive epithelial area following DSS exposure compared with WT controls (Figure 2H,I). In the independent fixed Day-7 cohort, KO females also exhibited greater Alcian Blue-positive epithelial area (Figure 2M,Q), indicating preservation of epithelial structure despite the absence of detectable genotype-associated differences in the broader clinical and histopathologic outcomes. We therefore interpreted the female phenotype as selective preservation of colonic architecture and mucin-associated epithelial coverage, rather than generalized resistance to colitis.

Taken together, these findings demonstrate sex-divergent effects of PAD2 deficiency on selected epithelial outcomes following DSS-induced injury. Formal genotype × sex interactions were detected for disease activity (*p* < 0.0001), colon length (*p* = 0.0413), and Alcian Blue-positive epithelial area (*p* = 0.0006), whereas the interaction for histopathologic injury was not significant. PAD2 deficiency was associated with increased disease severity and epithelial injury in males, whereas female PAD2 deficiency was associated with selective preservation of structural epithelial endpoints following DSS exposure. These findings support distinct sex-divergent phenotypic responses of PAD2 deficiency and sex during colonic injury.

### 3.3. Female PAD2 Deficiency Is Associated with Altered Microbial Community Structure

Because the epithelial phenotype associated with PAD2 deficiency differed markedly between sexes, the baseline fecal microbiome was characterized to determine whether PAD2 deficiency was accompanied by differences in microbial community composition. Shotgun metagenomic sequencing was performed on fecal samples from matched WT and KO littermates generated across independent heterozygous breeding cohorts.

**Figure 3:**
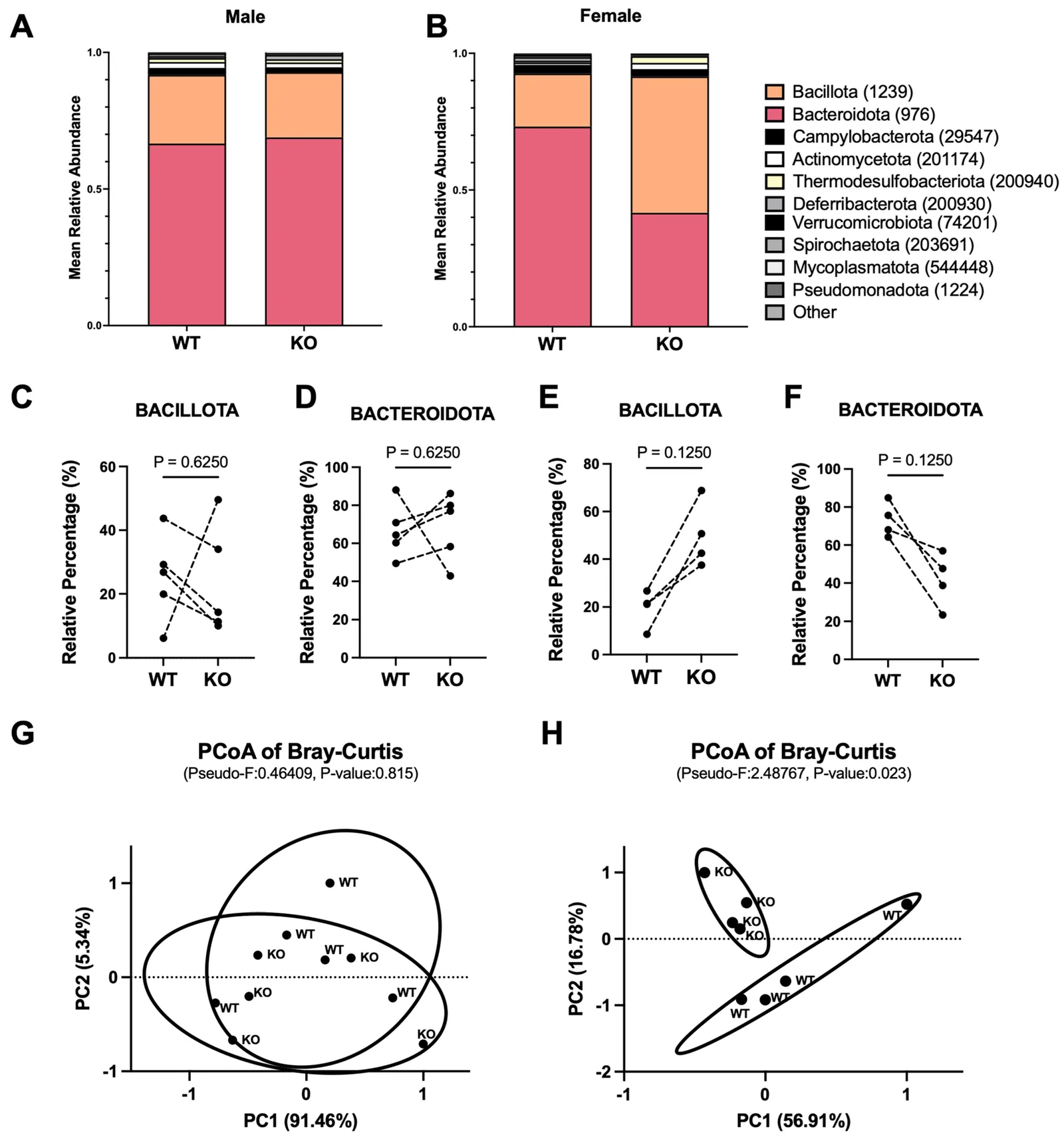
Female PAD2 deficiency is associated with differences in intestinal microbial community composition. (**A**,**B**) Relative abundance of the dominant bacterial phyla in fecal microbiota from male (**A**) and female (**B**) WT and PAD2 KO mice determined by shotgun metagenomic sequencing (male: n = 5 mice per genotype; female: n = 4 mice per genotype; matched littermates). (**C**–**F**) Relative abundances of Bacillota and Bacteroidota in male (**C**,**D**) and female (**E**,**F**) WT and PAD2 KO mice. Data are presented descriptively as paired individual values, with lines connecting matched WT and PAD2 KO littermates, and exact *p* values shown on each panel. (**G**,**H**) Principal coordinates analysis (PCoA) of genus-level microbial community composition in male (**G**) and female (**H**) mice based on Bray–Curtis dissimilarity. PERMANOVA showed no detectable genotype-associated difference in males (Pseudo-F = 0.46409, *p* = 0.815) and a genotype-associated difference in females (Pseudo-F = 2.48767, *p* = 0.023). Relative taxonomic abundances were compared using two-sided Wilcoxon matched-pairs signed-rank tests. Microbial community composition (β-diversity) was analyzed using PERMANOVA with 999 permutations.

At the phylum level, male WT and KO microbiome profiles were broadly similar, whereas female KO mice showed directional differences characterized by higher Bacillota and lower Bacteroidota relative abundance (Figure 3A–F). These phylum-level differences in females did not reach statistical significance due to the small sample size and are therefore presented descriptively. Overall microbial diversity was next examined by assessing richness and evenness using the Shannon and Simpson indices. No detectable genotype-associated differences in the measured α-diversity indices were observed in either sex (Figure S3A–D). β-diversity analyses identified genotype-associated differences in female microbial community composition. Principal coordinates analysis of Bray–Curtis dissimilarity demonstrated substantial overlap between WT and KO male microbiota, whereas female communities segregated by genotype (Figure 3G,H). PERMANOVA identified no genotype-associated difference among male communities (Pseudo-F = 0.46409, *p* = 0.815), whereas a genotype-associated difference was detected among female communities (Pseudo-F = 2.48767, *p* = 0.023). Given the modest cohort sizes and limited permutation space available for examining littermate–pair structure, these community-level PERMANOVA findings were considered exploratory. Similar clustering patterns were observed using weighted UniFrac distances (Figure S3E,F). PERMDISP analysis identified no significant difference in Bray–Curtis dispersion between genotypes in either males (*p* = 0.968) or females (*p* = 0.200), indicating that the observed community-level patterns were not attributable to detectable differences in within-group dispersion.

Exploratory functional profiling was additionally performed using MetaCyc pathway abundances. Across the 50 pathways evaluated, no individual pathway met FDR-adjusted significance in either male or female mice (Table S2). Given the small number of littermates and substantial interindividual variability, these predicted functional profiles are considered exploratory and were not used to infer specific alterations in metabolic activity. Collectively, these findings identify genotype-associated differences in female intestinal microbial community composition without detectable difference in the measured α-diversity indices.

### 3.4. Broad-Spectrum Antibiotics Alter Female PAD2-Associated Epithelial Phenotypes

The genotype-associated differences in microbial community composition prompted subsequent experiments to examine whether microbial disruption altered the female epithelial phenotype associated with PAD2 deficiency. Female WT and PAD2 KO mice were therefore treated with a broad-spectrum antibiotic cocktail (ABX) before DSS challenge.

**Figure 4:**
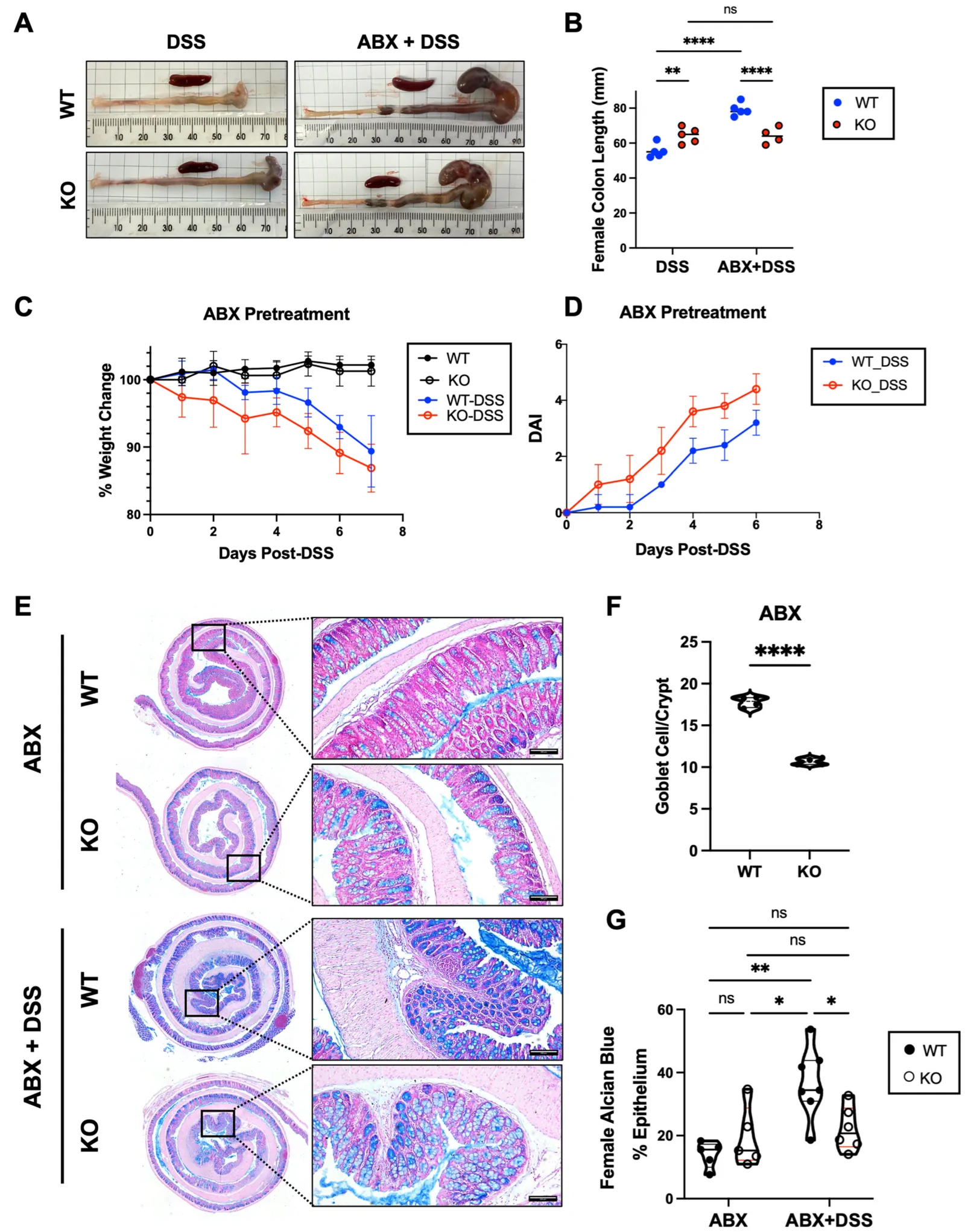
The female PAD2 KO phenotype is altered by broad-spectrum antibiotic treatment. (**A**,**B**) Representative gross colon images (**A**) and colon length measurements (**B**) from WT and PAD2 KO female mice following 4 weeks of ABX pretreatment and 7 days of 2.5% DSS. Individual data points represent one mouse (n = 4–5 mice per genotype). Male mice were evaluated as a contemporaneous arm of the same prolonged ABX experiment and are presented in Figure S3G,H. (**C**–**G**) Results from a separate short-term experiment using 5 days of ABX pretreatment followed by ABX alone or ABX plus 2.5% DSS, as indicated. (**C**,**D**) Percent body weight change ((**C**); *p* = 0.0271) and disease activity index (DAI) ((**D**); *p* = 0.0027) in WT and PAD2 KO female mice (n = 4–5 mice per genotype). (**E**) Representative Alcian Blue-stained Swiss-roll colon sections following ABX alone or ABX plus DSS. Higher-magnification medial and proximal insets illustrate Alcian Blue-positive epithelial coverage after DSS. Scale bar = 100 μm. (**F**) Violin plots of goblet cells per crypt following 5-day ABX treatment (n = 4 mice per genotype; 103 crypts quantified per mouse). Overlaid data points represent individual mouse means, which were used as the biological replicate for statistical analysis. (**G**) Alcian Blue-positive epithelial area following ABX treatment with or without DSS, presented as truncated violin plots with individual biological replicates shown (n = 5 WT + ABX, 5 KO + ABX, 7 WT + ABX + DSS, and 6 KO + ABX + DSS). Percent body weight change and DAI were analyzed using a mixed-effects model (REML) with Geisser–Greenhouse correction for repeated measures and Šídák-adjusted multiple comparisons. All other comparisons were analyzed using two-sided Welch’s *t* tests or ordinary two-way ANOVA followed by Šídák-adjusted multiple comparisons, as appropriate. * *p* < 0.05; ** *p* < 0.01; **** *p* < 0.0001. ns: not significant.

In a prolonged microbiome-disruption experiment, male and female WT and PAD2 KO mice received 4 weeks of ABX treatment followed by 7 days of 2.5% DSS exposure. Male and female mice were evaluated as contemporaneous arms of the same experiment. Within the female arm, KO mice exhibited longer colons than WT mice following DSS alone (*p* = 0.0040). However, this genotype-associated difference was reversed following ABX treatment, with ABX + DSS WT mice exhibiting longer colons than ABX + DSS KO mice (*p* < 0.0001; Figure 4A,B). This reversal was associated with reduced colonic shortening in WT mice following ABX treatment (DSS vs. ABX + DSS *p* < 0.0001), whereas colon length was unchanged in KO mice (*p* = 0.9582). This resulted in a significant genotype × treatment interaction (*p* < 0.0001).

In the contemporaneous male arm, KO mice had shorter colons than WT controls following both DSS alone (*p* < 0.0001) and ABX + DSS treatment (*p* < 0.0001; Figure S3G,H). In contrast to females, ABX treatment reduced DSS-associated colonic shortening in both male WT and KO mice (both *p* < 0.0001), while the genotype-associated difference remained directionally consistent. Formal sex × genotype analysis of the contemporaneous cohorts identified a significant interaction under DSS alone (*p* < 0.0001), whereas the sex × genotype interaction was not significant following ABX + DSS treatment (*p* = 0.2014).

In a separate short-term ABX experiment performed in females, 5 days of ABX pretreatment shifted the response of KO mice toward greater weight loss and disease activity during 2.5% DSS exposure (Figure 4C,D). Within this same short-term experiment, the effects of ABX treatment on goblet cell composition were next evaluated. Following 5 days of ABX alone, KO females displayed fewer goblet cells per crypt than WT controls (Figure 4E,F). Following 2.5% DSS challenge, WT mice exhibited greater Alcian Blue-positive epithelial coverage, whereas this response was not observed in KO females (Figure 4E,G).

Collectively, these findings demonstrate that the female epithelial phenotype associated with PAD2 deficiency is sensitive to broad-spectrum antibiotic treatment. Prolonged ABX treatment reversed the genotype-associated difference in female colon length, with WT but not KO females exhibiting reduced colonic shortening. In contrast, both male genotypes exhibited reduced colonic shortening, with the genotype-associated difference maintained. In the separate short-term experiment, ABX treatment was accompanied by greater disease activity and altered epithelial features in female KO mice during DSS exposure. Together, these findings support an association between antibiotic-sensitive factors and the female PAD2 KO phenotype.

### 3.5. PAD2 Deficiency Is Associated with Distinct β-Catenin States in Male and Female Colons

**Figure 5:**
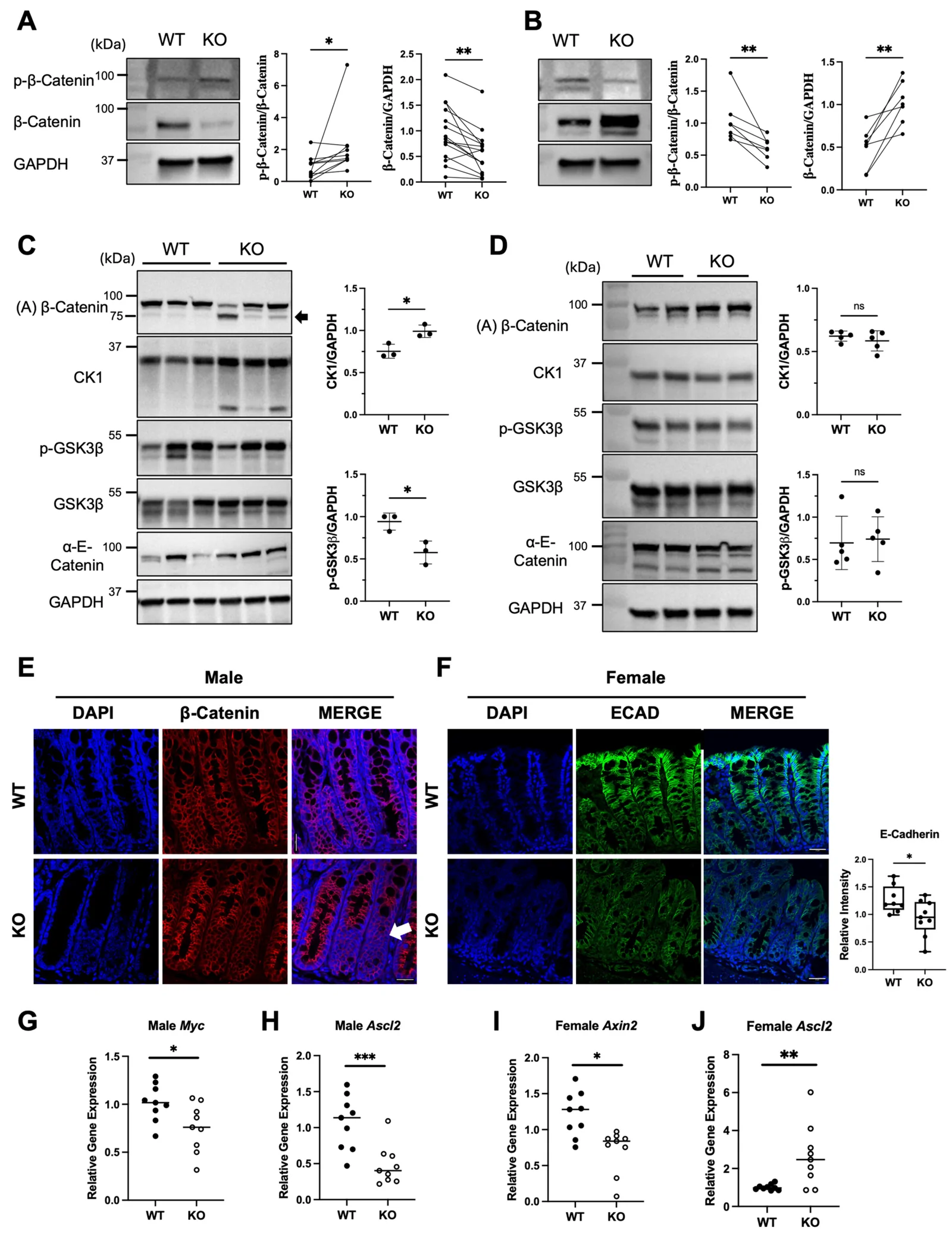
PAD2 deficiency is associated with distinct β-catenin regulatory states in male and female colons. (**A,B**) Representative immunoblots and densitometric quantification of phosphorylated β-catenin, total β-catenin, and GAPDH in proximal colon lysates from male (**A**) and female (**B**) WT and PAD2 KO mice. Phosphorylated β-catenin was normalized to total β-catenin (male; n = 10 mice per genotype, female; n = 7 mice per genotype), and total β-catenin was normalized to GAPDH (male; n = 15 mice per genotype, and female; n = 7 mice per genotype). Individual WT and PAD2 KO littermate pairs are connected by a solid black line. Data were analyzed using two-sided paired *t*-tests. Original Western blot images can be found in Supplementary Materials. (**C**,**D**) Representative immunoblots from male (**C**) and female (**D**) WT and PAD2 KO proximal colon lysates showing active β-catenin (A β-catenin), casein kinase 1 (CK1), phosphorylated glycogen synthase kinase-3β (p-GSK3β), total GSK3β, αE-catenin, and GAPDH. Densitometric quantification of CK1 and p-GSK3β normalized to GAPDH in males (n = 3 mice per genotype) and females (n = 5 mice per genotype). Data are presented as mean ± SD with individual biological replicates shown. A lower-molecular-weight β-catenin immunoreactive species in C is indicated by a black arrow. Original Western blot images can be found in Supplementary Materials. (**E**) Representative immunofluorescence images of total β-catenin (red) and nuclei (DAPI, blue), and merged images in male colonic crypts. The white arrow indicates altered β-catenin localization within PAD2 KO crypts. Scale bar = 25 μm. (**F**) Representative immunofluorescence of E-cadherin (green), nuclei (DAPI, blue), and merged images in female WT and PAD2 KO colon. Data are presented as a box-and-whisker plots showing the median, interquartile range, and minimum-to-maximum values, with individual mice shown (WT, n = 8; KO, n = 9). Scale bar = 25 μm. (**G**,**H**) Relative *Myc* (**G**) and *Ascl2* (**H**) mRNA expression in male WT and PAD2 KO colons (n = 9 mice per genotype). (**I**,**J**) Relative *Axin2* (**I**) and *Ascl2* (**J**) mRNA expression in female WT and PAD2 KO colons (n = 9 mice per genotype). Individual biological replicates are shown with the median indicated. Total β-catenin/GAPDH was analyzed using two-sided paired *t*-test, whereas p-β-catenin/total β-catenin was log-transformed and analyzed using a paired *t*-test. CK1 and p-GSK3β densitometry and E-cadherin immunofluorescence were analyzed using two-sided Welch’s *t* tests. qPCR data were analyzed using two-way ANOVA with genotype and qPCR run as factors, followed by Šídák-adjusted planned comparisons between WT and PAD2 KO mice. * *p* < 0.05; ** *p* < 0.01; *** *p* < 0.001. ns: not significant.

Because β-catenin is a reported substrate of PAD2-mediated citrullination [17] and a central regulator of epithelial proliferation and differentiation, we next sought to determine whether the sex-specific epithelial observations were accompanied by distinct alterations in β-catenin regulation. Male KO colons showed increased phosphorylation of β-catenin together with reduced total β-catenin abundance (Figure 5A). Direct normalization of phospho-β-catenin to GAPDH additionally demonstrated that the increase was not attributable solely to the accompanying reduction in total β-catenin (Figure S4A). Additional components of the β-catenin destruction complex were further evaluated. Immunoblotting for active β-catenin identified a prominent lower-molecular-weight immunoreactive species at approximately 75 kDa in KO males. Increased CK1 and decreased p-GSK3β abundance were also observed (Figure 5C). Because the identity of the lower-molecular-weight species was not independently validated, whether it represents a product of β-catenin processing or proteolysis remains unclear. Complementary immunofluorescent microscopy demonstrated redistribution of β-catenin staining toward the lower crypt in male KO mice (Figure 5E).

Female KO colons exhibited a distinct biochemical phenotype, characterized by reduced degradation-associated phospho-β-catenin and increased total β-catenin abundance, accompanied by comparatively limited changes in CK1 or p-GSK3β levels (Figure 5B,D). E-cadherin immunofluorescence was also reduced in female KO colons (Figure 5F).

Consistent with these protein-level changes, expression of β-catenin associated target genes also differed in WT and KO mice in a sex-specific manner. Male KO mice exhibited reduced expression of *Myc* and *Ascl2* (Figure 5G,H), whereas female KO mice demonstrated reduced *Axin2* expression and increased *Ascl2* (Figure 5I,J). Complementary analyses of epithelial proliferation and additional β-catenin-associated signaling targets, including BrdU incorporation, p21, Cyclin D1, *Lgr5*, *Sox9,* and *Ctnnb1* are presented in Figure S4.

Taken together, these data identify distinct β-catenin states associated with PAD2 deficiency in males and females. Male PAD2 deficiency was associated with increased β-catenin phosphorylation and reduced total β-catenin abundance, whereas female PAD2 deficiency was associated with reduced β-catenin phosphorylation and increased total β-catenin abundance. These biochemical states accompanied the divergent epithelial phenotypes observed in male and female PAD2 KO mice.

### 3.6. Antibiotic-Sensitive Fecal PAD2-Immunoreactive Proteins Accompany Epithelial β-Catenin Remodeling

**Figure 6:**
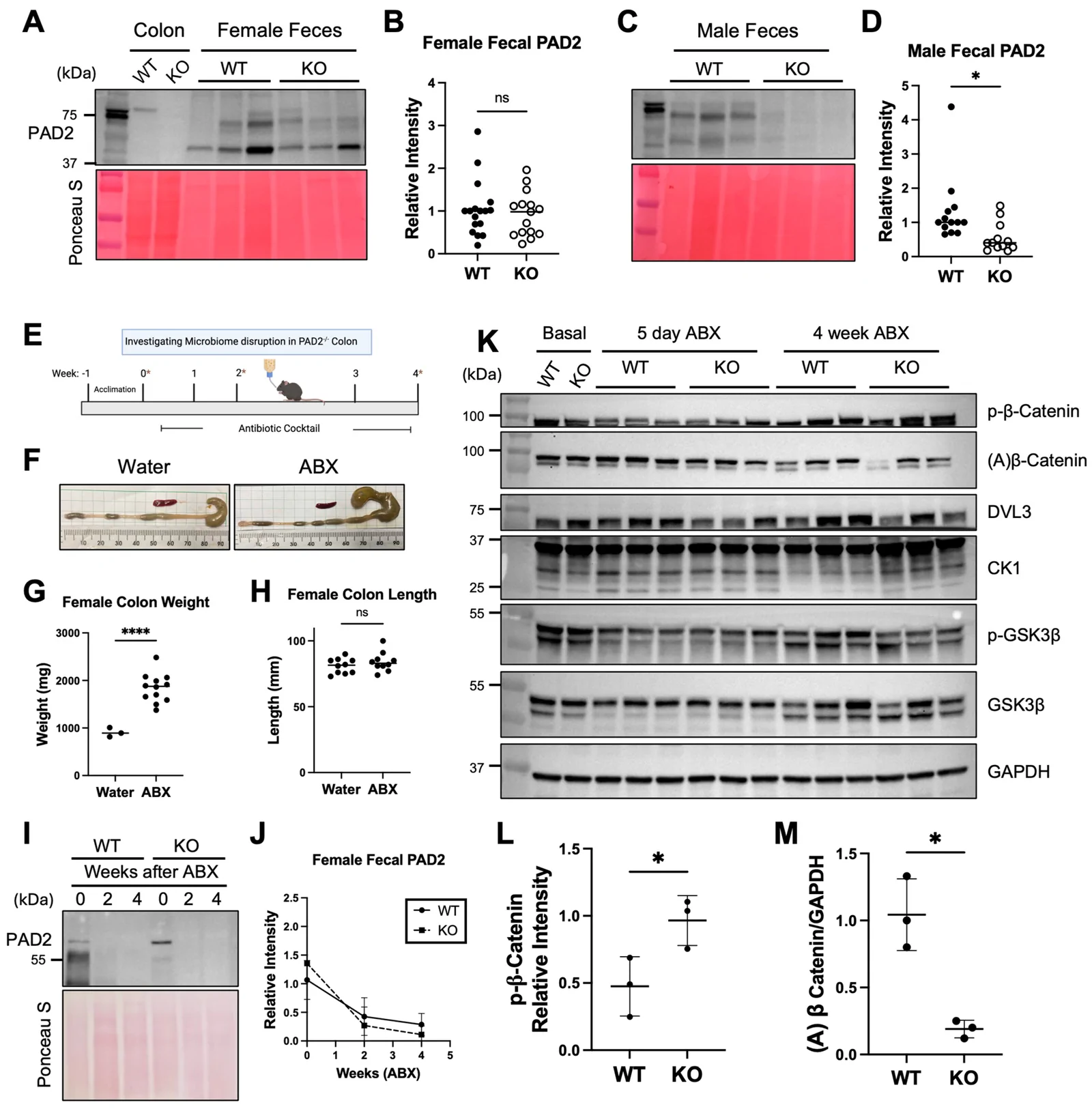
Antibiotic treatment alters fecal PAD2 immunoreactivity and epithelial β-catenin states. (**A**,**B**) Representative immunoblot (**A**) and densitometric quantification (**B**) of PAD2-immunoreactive proteins in fecal lysates from female WT and PAD2 KO mice, showing heterogeneous immunoreactivity across multiple molecular weights. Total protein staining (Ponceau S) was used as the loading control. WT and PAD2 KO colon lysates served as positive and negative antibody controls, respectively. PAD2 immunoreactivity was normalized to total protein (n = 15 mice per genotype). Original Western blot images can be found in Supplementary Materials. (**C**,**D**) Representative immunoblot (**C**) and densitometric quantification (**D**) of PAD2-immunoreactive proteins in fecal lysates from male WT and PAD2 KO mice. Total protein staining (Ponceau S) was used as the loading control, and PAD2 immunoreactivity was normalized to total protein (n = 13 mice per genotype). (**E**) Experimental timeline illustrating broad-spectrum antibiotic (ABX) administration, with fecal collection time points indicated in brown asterisks. (**F**–**H**) Representative gross colon images (**F**), cecal-colon mass ((**G**); n = 3 H_2_O, n = 12 ABX [n = 6 WT, n = 6 KO]), and colon length ((**H**); n = 10 H_2_O, n =10 ABX [n = 5 WT, n = 5 KO]) from female WT and PAD2 KO mice following 4 weeks of ABX treatment. Individual points represent biological replicates. For (**G**,**H**), WT and KO ABX-treated female mice were compared by genotype prior to pooling (*p* = 0.2871 and *p* = 0.7259, respectively). For (**H**), the H_2_O group consisted of historical untreated controls from the basal cohort represented in Figure 1 (n = 5 WT and n = 5 KO). Individual points represent biological replicates. (**I**,**J**) Representative immunoblot (**I**) and longitudinal densitometric quantification (**J**) of fecal PAD2 immunoreactivity during ABX treatment (n = 11 mice). (**K**) Representative immunoblots of phosphorylated β-catenin, active β-catenin, Dishevelled-3 (DVL3), casein kinase 1 (CK1), phosphorylated glycogen synthase kinase-3β (p-GSK3β), total GSK3β, and GAPDH from female colons collected at baseline, day 5, and week 4 of ABX treatment. Original Western blot images can be found in Supplementary Materials. (**L**,**M**) Densitometric quantification of phosphorylated β-catenin (**L**) and active β-catenin (**M**) normalized to GAPDH at week 4 of ABX treatment (n = 3 mice per genotype). Data in (**B**,**D**,**G**,**H**) are presented with individual biological replicates and the median indicated; (**J**) is presented as mean ± SEM; and (**L**,**M**) are presented as mean ± SD with individual biological replicates. Longitudinal fecal PAD2 immunoreactivity was analyzed using a mixed-effects model (REML) with Geisser–Greenhouse correction for repeated measures followed by Šídák-adjusted multiple comparisons. Comparisons between two groups were analyzed using two-sided Welch’s *t* tests. * *p* < 0.05; **** *p* < 0.0001. ns: not significant.

Because broad-spectrum antibiotic treatment altered the female KO injury phenotype, fecal protein extracts were next examined to determine whether luminal PAD-reactive proteins accompanied epithelial remodeling. Immunoblotting identified a heterogeneous pattern of fecal PAD2 immunoreactivity, including higher-molecular-weight signal and prominent species migrating at approximately 55 kDa and 37 kDa, with the relative prominence of these species varying among samples. PAD2 immunoreactivity was detectable in both female WT and KO samples with no significant genotype difference, whereas overall PAD2 immunoreactivity was reduced in male KO feces (Figure 6A–D). Because immunoreactive species persisted in PAD2-deficient mice, these signals cannot be assumed to represent host PAD2 and may instead reflect microbial proteins or antibody cross-reactivity. Accordingly, these species are referred to throughout as PAD2-immunoreactive fecal proteins. Broad-spectrum antibiotic treatment was associated with a progressive reduction in fecal PAD2-immunoreactive proteins in both sexes (Figure 6I,J; Figure S3K). ABX treatment produced expected physiological effects, including increased cecal-colon mass [47,48], whereas no detectable change in colon length was observed (Figure 6E–H; Figure S3I,J).

Because broad-spectrum antibiotic treatment altered the structural difference observed between female WT and KO mice during DSS treatment, β-catenin remodeling was examined during longitudinal antibiotic treatment. Proximal colon lysates collected at baseline, Day 5, and Week 4 were examined for components of the β-catenin degradation machinery (Figure 6K). The representative time-course immunoblots show qualitative differences in degradation-associated phospho-β-catenin and active β-catenin, DVL3, CK1, GSK3β, and p-GSK3β during antibiotic exposure. However, these intermediate time points were not quantitatively analyzed. At Week 4, densitometric analysis demonstrated increased degradation-associated phospho-β-catenin and reduced active β-catenin in KO females compared to WT controls (Figure 6L,M).

Collectively, antibiotic treatment was accompanied by both a progressive loss of fecal PAD2-immunoreactive proteins and altered epithelial β-catenin abundance after prolonged treatment. Because the fecal immunoreactivity declined in both genotypes and did not differ significantly between female WT and KO mice at baseline, these parallel observations do not establish that the fecal PAD2-immunoreactive proteins mediate the female epithelial phenotype.

### 3.7. Exogenous PAD2 Is Associated with Increased Citrullination and Reduced Active β-Catenin in HT29 Cells

**Figure 7:**
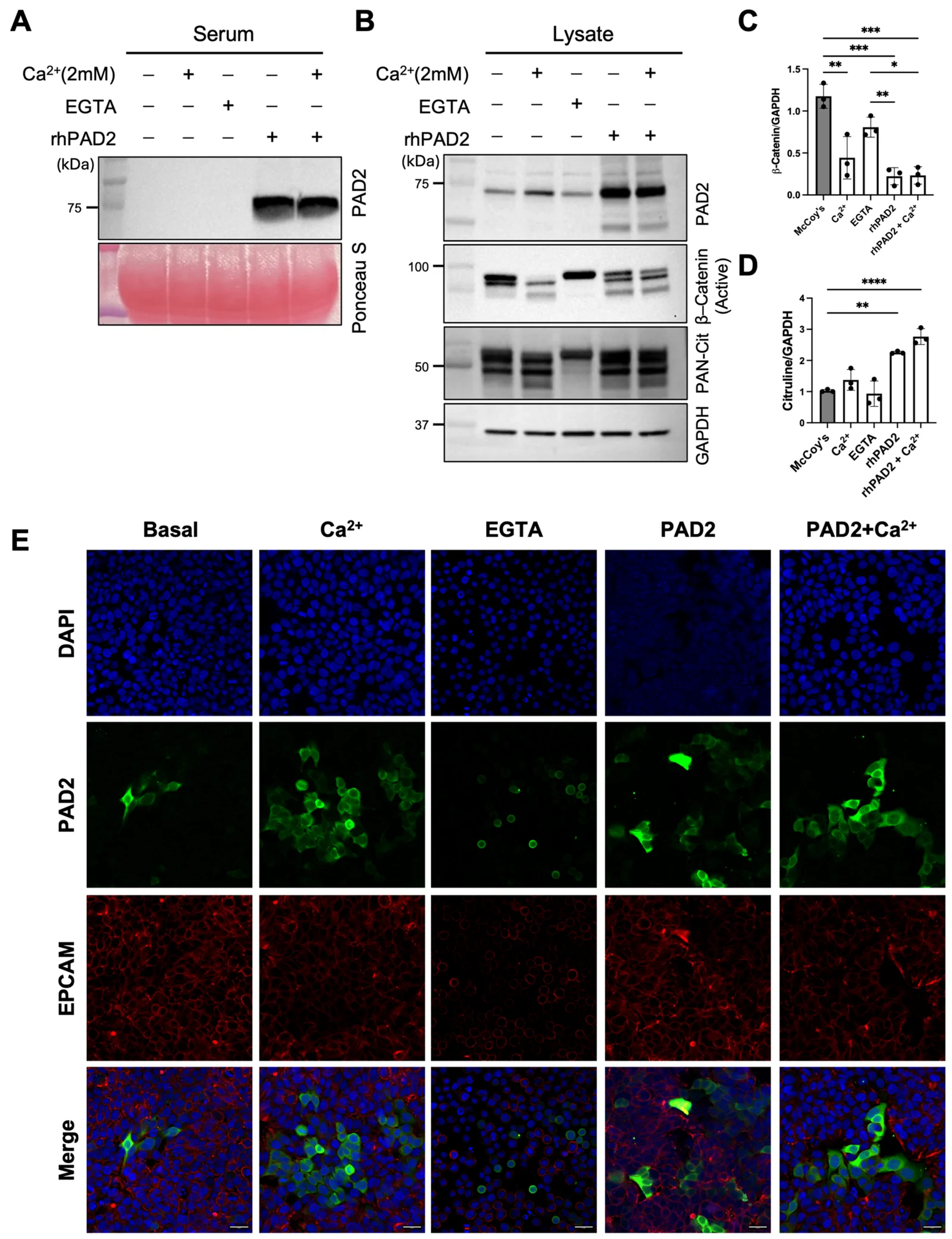
Exogenous PAD2 treatment is associated with citrullination and reduced active β-catenin in HT29 cells (**A**) Representative immunoblot of conditioned medium collected from HT29 cells following 24 h of treatment in McCoy’s 5A medium with 2 mM CaCl2, 2 mM EGTA, 0.5 μg/mL recombinant human PAD2 (rhPAD2), or the indicated combinations. PAD2 immunoreactivity was normalized to total protein (Ponceau S). (**B**) Representative immunoblot of PAD2, active β-catenin, pan-citrulline, and GAPDH in HT29 cell lysates following the indicated 24-h treatments. Original Western blot images can be found in Supplementary Materials. (**C**,**D**) Densitometric quantification of active β-catenin (**C**) and pan-citrulline (**D**) normalized to GAPDH from three independent experiments. (**E**) Representative immunofluorescence images of HT29 cells stained for PAD2 (green), EPCAM (red), and nuclei (DAPI, blue) following the indicated treatments. Representative fields illustrate heterogeneous PAD2 immunoreactivity within the epithelial population. Scale bar = 25 μm. Data are presented as mean ± SD. In vitro treatment groups were analyzed using ordinary one-way ANOVA followed by Šídák-adjusted planned multiple comparisons. * *p* < 0.05; ** *p* < 0.01; *** *p* < 0.001; **** *p* < 0.0001.

The association between antibiotic-sensitive fecal PAD2-immunoreactive proteins and epithelial β-catenin remodeling prompted us to examine whether exogenous PAD2 treatment could alter epithelial β-catenin regulation. To test this possibility, HT29 cells, which express endogenous PAD2, were exposed to recombinant human PAD2 (rhPAD2) under calcium-manipulated conditions. Addition of CaCl_2_ alone reduced active β-catenin relative to McCoy’s 5A medium alone (1.175 ± 0.144 vs. 0.443 ± 0.253; adjusted *p* = 0.0018), indicating that calcium manipulation itself influenced β-catenin abundance. Importantly, rhPAD2 treatment without supplemental calcium reduced active β-catenin relative to McCoy’s 5A alone (1.175 ± 0.144 vs. 0.223 ± 0.102; adjusted *p* = 0.0002; Figure 7B,C). Addition of calcium to rhPAD2 did not further reduce active β-catenin (0.223 ± 0.102 vs. 0.233 ± 0.104; adjusted *p* > 0.9999).

Calcium alone also produced a non-significant increase in pan-citrulline immunoreactivity relative to McCoy’s 5A alone (1.025 ± 0.043 vs. 1.377 ± 0.335; adjusted *p* = 0.5088). In contrast, rhPAD2 alone significantly increased pan-citrulline immunoreactivity (1.025 ± 0.043 vs. 2.255 ± 0.041; adjusted *p* = 0.0014), with greater pan-citrullination following rhPAD2 than calcium alone (adjusted *p* = 0.0146; Figure 7B,D). Immunofluorescence demonstrated heterogeneous association of exogenous rhPAD2 with the epithelial population (Figure 7E).

Together, these findings associate exogenous PAD2 treatment with increased epithelial protein citrullination and reduced active β-catenin abundance in vitro, including in the absence of supplemental calcium. Although these experiments do not establish that PAD2 catalytic activity directly mediates the β-catenin response, they provide a framework in which extracellular PAD2 may modulate epithelial proteins and collectively support future interrogation of extracellular PAD2 and PAD2-immunoreactive luminal proteins as potential modulators of epithelial signaling.

## 4. Discussion

This study identifies PAD2 as a context-dependent modulator in epithelial homeostasis and colonic injury. Four principal findings define this conceptual advance. First, constitutive PAD2 deficiency is associated with distinct basal epithelial states in males and females before the onset of injury. Second, these basal states precede divergent responses to epithelial damage, with PAD2 deficiency associated with greater DSS-induced injury in males, whereas female PAD2-deficient mice exhibit preservation of selected structural epithelial features whose relationship to genotype is altered following broad-spectrum antibiotic treatment. Third, PAD2 deficiency is associated with distinct β-catenin states, characterized by a degradation-associated β-catenin profile in males and preservation of β-catenin protein in females. Finally, the female phenotype is accompanied by genotype-associated differences in intestinal microbial community composition, together with antibiotic-sensitive luminal PAD2-immunoreactive proteins. Together, these findings extend intestinal PAD biology beyond its established role in inflammatory citrullination and identify PAD2 as a candidate interface between epithelial signaling, biological sex, and host–microbial interactions. For comparison, the major sex and treatment phenotypes observed across experimental conditions are summarized in Table 1.

Although pharmacologic PAD inhibition has demonstrated benefit in several experimental colitis studies, these approaches have largely focused on inflammatory citrullination, neutrophil extracellular trap formation, or PAD4-dependent mechanisms [21,22,24,25]. The present findings caution against assumptions that individual PAD isoforms contribute uniformly to intestinal disease. In the present DSS model, PAD2 deficiency increased susceptibility to epithelial injury in males, while female PAD2 deficiency preserved selected structural epithelial endpoints. Because colon length in the longitudinal survival cohort was measured at terminal collection and DSS exposure duration varied according to disease progression, these measurements were interpreted alongside independent fixed Day-7 histological and epithelial analyses rather than as standardized fixed-time comparisons. Moreover, both PAD2 and PAD4 protein abundance increased following DSS exposure, whereas global protein citrullination declined (Figure S2C–H). These observations emphasize that PAD abundance should not be interpreted as a surrogate for enzymatic activity. Calcium availability, cellular localization, substrate accessibility, oxidative conditions, endogenous inhibitors, and enzyme inactivation may each uncouple protein abundance from net citrullination during intestinal injury.

A recent report demonstrated that PAD2 deficiency attenuated TNBS-induced colitis and was accompanied by altered macrophage polarization and reduced inflammatory cytokine production [23]. Although these findings appear inconsistent with the present study, several biological differences between experimental models may contribute to the apparent discrepancy. TNBS and DSS initiate intestinal injury through distinct mechanisms, with TNBS involving prominent hapten-driven immune responses and DSS initiating injury through disruption of the epithelial barrier [49,50,51]. In addition, the previous study examined only male animals and focused on macrophage biology, whereas the present work resolves responses by biological sex and centers on epithelial β-catenin regulation. Rather than representing conflicting observations, these studies collectively suggest that PAD2 function varies among the dominant tissue compartment, mechanism of injury, and biological sex. Such context dependence may prove critical when considering therapeutic strategies directed toward individual PAD isoforms.

Importantly, the sex-divergent phenotypes identified in this study were evident before epithelial injury, supporting a role for PAD2 in basal intestinal epithelium in addition to its well-defined roles following injury and inflammation. Male PAD2-deficient mice exhibited reduced goblet cell abundance together with reduced *Klf4* expression, basal epithelial features that may contribute to increased susceptibility during DSS-induced injury. In contrast, female PAD2-deficient mice developed deeper crypts accompanied by reduced degradation-associated β-catenin phosphorylation and increased total β-catenin abundance. Whether this state contributes to the preservation of selected epithelial features observed following DSS injury remains to be determined. Similar sex dimorphisms in PAD2 biology have been reported in the brain, heart, and developing fetus, although the mechanisms responsible remain poorly understood [52,53,54]. Sex hormones, immune tone, epigenetic regulation, microbial composition and microbial metabolites each represent plausible contributors to the sex-specific epithelial states observed in this study. Whether these factors regulate PAD2 expression directly or instead modify PAD2 substrate availability remains an important question for future investigation.

β-Catenin regulation provides the strongest mechanistic connection between PAD2 deficiency and the epithelial phenotypes observed in this study. Early findings from patient tissues and biochemical studies identified β-catenin as a substrate of PAD2-mediated citrullination while also demonstrating that citrullination can reduce β-catenin stability and Wnt signaling [16,17]. Our findings extend these observations to the intact colon by identifying distinct β-catenin regulatory states in vivo. Male PAD2 deficiency was characterized by degradation-associated β-catenin phosphorylation, reduced total β-catenin abundance, increased CK1 expression, altered crypt localization, and the appearance of a lower-molecular-weight β-catenin-immunoreactive species. Although this biochemical profile is consistent with altered β-catenin stability, the molecular identity of the lower-molecular-weight species was not independently validated. Proteolytic cleavage has been previously reported, although it cannot be concluded that the species observed here represents a β-catenin proteolytic fragment [55,56]. In contrast, female PAD2 deficiency was associated with increased total β-catenin, reduced degradation-associated phosphorylation, and remodeling of epithelial architecture, consistent with a fundamentally distinct regulatory state.

One of the more unexpected findings was the discordance between β-catenin protein abundance and downstream transcriptional responses. Although female PAD2 deficiency increased β-catenin abundance and was accompanied by evidence of enhanced epithelial proliferation, *Axin2* expression was reduced, arguing against generalized activation of canonical Wnt signaling [57]. PAD2 deficiency therefore appears to alter β-catenin regulation in a context-dependent manner rather than functioning as a simple switch for canonical pathway activity. Differences in β-catenin stability, junctional sequestration, subcellular localization, or transcriptional competence may each contribute as factors to this state. In males, increased β-catenin phosphorylation and reduced total β-catenin was accompanied by altered expression of *Myc* and *Ascl2*. Together, these findings suggest that PAD2 deficiency can uncouple β-catenin abundance from uniform canonical transcriptional output, with the resulting epithelial states likely reflecting multiple levels of regulation.

These findings are consistent with PAD2-dependent regulation of β-catenin but do not establish direct β-catenin citrullination as the causal mechanism in vivo. Whole-colon immunoprecipitation after DSS demonstrated reduced β-catenin citrullination together with altered β-catenin abundance. However, these analyses do not distinguish epithelial-intrinsic effects from differences in cellular composition (Figure S2I,J). Defining this mechanism will require epithelial-directed manipulation of PAD2, direct identification of citrullinated β-catenin residues by mass spectrometry, and functional interrogation of citrullination-resistant β-catenin variants. Likewise, primary intestinal cultures from male and female WT and PAD2-deficient mice may provide an additional platform for distinguishing epithelial-intrinsic regulation from systemic and microbiome-associated influences.

The microbiome findings introduce a second level of regulation. Although no genotype-associated differences were detected in the measured α-diversity indices, female PAD2 deficiency was accompanied by genotype-associated differences in microbial community composition. Exploratory analysis of predicted MetaCyc pathway abundances identified directional differences in microbial functional potential. However, no individual pathway remained significant following FDR correction. While several arginine-associated pathways showed directional differences in the exploratory analysis, these findings did not support inference of altered arginine metabolism from the present metagenomic data. Because sequence-based functional predictions reflect metabolic potential rather than metabolic activity, direct measurement of luminal arginine and other microbial metabolites will be necessary to determine whether the genotype-associated differences in microbial community composition are accompanied by functional metabolic changes that contribute to the female epithelial phenotype.

The antibiotic experiments provide functional evidence that the epithelial phenotypes associated with PAD2 deficiency are sensitive to antibiotic treatment. In females, prolonged antibiotics reversed the genotype-associated colon length difference observed during DSS injury. Whereas PAD2 KO females exhibited reduced colonic shortening under DSS alone, WT females exhibited increased colon length, with KO colon length unchanged following ABX + DSS. Moreover, ABX treatment also reduced the overall colonic shortening in both WT and KO males in the contemporaneous arm, indicating that ABX-mediated colonic shortening was not unique to female WT mice. Accordingly, the female result is most conservatively interpreted as a reversal of the genotype-associated colon length relationship, rather than as evidence for a selective female WT response to antibiotics.

Importantly, antibiotics also influence host metabolism, immune signaling, and epithelial physiology independently of microbial depletion [58,59,60]. Both prolonged and short ABX treatment produced marked cecal enlargement without a corresponding change in colon length in the absence of DSS (Figure 6G,H; Figure S3I,J), demonstrating additional gross intestinal effects of antibiotic exposure independent of DSS. Additionally, microbiota depletion has been observed to modify DSS-associated injury, although the magnitude and direction of these effects vary with antibiotic selection and experimental context [61,62]. Thus, the reduction in colonic shortening observed following ABX + DSS may reflect broader antibiotic-associated changes in DSS susceptibility and gastrointestinal physiology, rather than restoration of loss of a single PAD-2 dependent structural phenotype.

Together, these findings demonstrate that the relationship between PAD2 and epithelial responses to DSS is altered in a setting of broad-spectrum antibiotic exposure, while not distinguishing microbial disruption from direct or secondary host effects of the compounds themselves. Fecal microbiota transplantation, defined microbial communities, and targeted metabolomic studies will be important for determining whether the female phenotype is transferable and whether specific microbial factors contribute to the PAD2-associated epithelial phenotypes.

The antibiotic-sensitive fecal PAD2-immunoreactive proteins represent an intriguing observation accompanying microbial disruption. Their persistence in PAD2-deficient mice and progressive reduction during broad-spectrum antibiotic treatment suggest that their abundance is influenced by the intestinal microbial environment, although these findings do not establish their molecular origin. Interestingly, several of the fecal PAD2-immunoreactive species migrated within the molecular-weight range previously reported for selected bacterial PAD homologs [35]. However, immunoreactivity and molecular weight are insufficient to establish molecular identity or origin, and persistence of these species in PAD2-deficient mice could reflect microbial proteins, antibody cross-reactivity, or other immunoreactive fecal proteins. Mass spectrometry, recombinant-protein comparison, and direct PAD activity assays will be necessary to establish their origin and biochemical function.

Our in vitro experiments provide a complementary proof of principle: exogenous rhPAD2 increased cellular citrullination and reduced active β-catenin abundance, supporting the capacity of extracellular PAD2 to alter epithelial protein citrullination and substrate modulation. Because HT29 cells express endogenous PAD2, and PAD enzymes are calcium sensitive, the response to supplemental calcium may reflect multiple calcium-dependent processes, rather than endogenous PAD activity alone. Consistent with this possibility, calcium alone increased mean pan-citrulline immunoreactivity, although this increase was not statistically significant and was smaller than that observed following rhPAD2 treatment. Importantly, the association between exogenous PAD2 and reduced active β-catenin did not depend on supplemental calcium. rhPAD2 alone significantly reduced active β-catenin relative to McCoy’s 5A medium, while supplemental calcium did not further reduce active β-catenin in rhPAD2-treated cells.

Notably, the direction of the HT29-rhPAD2 response is consistent with the female in vivo phenotype, in which PAD2 deficiency was associated with increased β-catenin abundance, but does not directly explain the degradation-associated β-catenin state observed in PAD2-deficient males. The in vitro experiments were intended as a reductionist epithelial proof-of-principle rather than a model of the sex-specific phenotypes observed in vivo, as the divergent male phenotype indicates that additional biological context not represented in the cell-culture system may contribute to the divergent mouse phenotypes. Moreover, extracellular calcium manipulation can independently influence epithelial signaling. However, the rhPAD2-associated reduction in active β-catenin was also observed without supplemental calcium. These experiments nevertheless do not establish that the observed β-catenin response requires PAD2 catalytic activity. Direct evaluation using a catalytically inactive PAD2 preparation or validated pharmacologic inhibition will be needed to establish catalytic dependence. These experiments also do not establish that the fecal PAD2-immunoreactive proteins exert equivalent activity in vivo. Indeed, their abundance may instead reflect a broader microbiota-associated luminal state that changes in parallel with epithelial β-catenin regulation. Alternatively, if these proteins are microbial PAD homologs, differences in catalytic properties, substrate specificity, or extracellular activity could distinguish their effects from those of mammalian PAD2. Biochemical identification and functional characterization of these proteins will therefore be necessary to determine whether they participate directly in epithelial regulation or instead serve as markers of a microbial luminal state.

Several limitations define the next phase of this work. The current PAD2 KO cannot distinguish epithelial, immune, stromal, or developmental contributions to the observed phenotypes. Although loss of PAD2 protein confirmed the KO phenotype, a contribution from linked genetic variation cannot be completely excluded. This PAD2-deficient line has been used in previous studies [63]; however, validation using an independent or conditional model would further distinguish PAD2-specific from linked genetic effects. Operator-dependent morphometric and image analysis were not uniformly performed blinded to sex or genotype, which may represent a limitation of the present study. Additionally, estrous cycle stage, gonadal weights, and circulating sex hormone concentrations were not assessed, limiting our ability to determine whether systemic hormonal differences contribute to the sex-divergent phenotypes observed in PAD2-deficient mice. Thus, the present study cannot distinguish local PAD2 effects from those related to hormonal status or interactions between these factors.

In addition, although the microbiome analyses identified genotype-associated differences in female community composition, the modest cohort size warrants validation in larger, independently replicated studies designed to further resolve genotype effects from litter, cohort, and other environmental sources of variation. Establishing causality will ultimately require epithelial-specific PAD2 manipulation, transfer of PAD2-deficient microbiota, and biochemical identification with functional characterization of the luminal PAD2-immunoreactive proteins. Extension of these observations to sex-stratified human IBD cohorts may also be important for determining their clinical relevance.

## 5. Conclusions

The present study expands intestinal PAD biology beyond inflammatory citrullination by demonstrating context-dependent epithelial consequences of PAD2 deficiency. Rather than producing a uniform response, PAD2 deficiency resulted in distinct epithelial regulatory states in males and females, with the female phenotype additionally demonstrating sensitivity to broad-spectrum antibiotics. Together, these findings indicate that the effects of PAD2 deficiency differ according to biological sex and the surrounding environment, highlighting the importance of biological context when considering PAD2 function in intestinal maintenance and disease. To our knowledge, this study provides the first integrated characterization of PAD2 across male and female epithelial architecture, β-catenin regulation, and antibiotic-sensitive responses. More broadly, these findings position PAD2 biology as a candidate interface among epithelial signaling, biological sex, and the intestinal microbial environment, establishing a foundation for defining the cellular, biochemical, and microbial mechanisms underlying PAD2 function in the colon.

## Figures and Tables

**Table 1:** Summary of epithelial, microbiome, and β-catenin phenotypes associated with PAD2 deficiency. (ND: Not Described). Additional Bold heading within table indicates treatment condition.

Biological Feature	WT Male	KO Male	WT Female	KO Female	Figure Panel(s)
Basal Homeostasis					
Crypt depth	Reference	Unchanged	Reference	Increased	Figure 1D,G
Goblet cells	Reference	Decreased	Reference	Unchanged	Figure 1E,H
*Klf4* expression	Reference	Decreased	Reference	Unchanged	Figure 1I,J
β-catenin abundance	Reference	Decreased	Reference	Increased	Figure 5A,B
Phospho-β-catenin	Reference	Increased	Reference	Decreased	
BrdU labeling	ND	ND	Reference	Increased	Figure S4B
Microbiome composition	Reference	No Detectable genotype-associated difference	Reference	Genotype-associated difference	Figure 3; Figure S3A–F
Fecal PAD2-immunoreactive proteins	Present	Reduced	Present	Present	Figure 6A–D
**3% DSS**					
Disease activity	Reference	Increased	Reference	Unchanged	Figure 2D,E
Survival	Reference	Decreased	Reference	Unchanged	Figure 2B,C
Intestinal permeability	Reference	Increased	Reference	Unchanged	Figure S2A,B
Colon length	Reference	Unchanged	Reference	Preserved	Figure 2F–I
Histopathology	Reference	Worse	Reference	Unchanged	Figure 2J,L,N,P
Alcian Blue-positive epithelium	Reference	Decreased	Reference	Preserved	Figure 2K,M,O,Q
**ABX Alone**					
Goblet cells	ND	ND	Reference	Decreased	Figure 4E,F
β-catenin state	ND	ND	Reference	Decreased active, increased phospho-β-catenin	Figure 6K–M
Fecal PAD2-immunoreactive proteins	Decreased	Decreased	Decreased	Decreased	Figure 6I,J; Figure S3K
**ABX + DSS**					
Disease activity	ND	ND	Reference	Increased	Figure 4D
Colon length	Reference	Decreased: genotype difference maintained	Reference	Decreased: genotype difference reversed	Figure 4A,B; Figure S3G,H
Alcian Blue-positive epithelium	ND	ND	Reference	Decreased	Figure 4E,G

## Data Availability

The metagenomic sequencing data generated in this study are publicly available through the NCBI Sequence Read Archive under BioProject accession PRJNA1508927. Original uncropped immunoblot images underlying the reported immunoblot analyses are provided as Supplementary Source Data. Additional raw numerical data supporting the conclusions of this article will be made available by the corresponding author upon reasonable request.

## Supplementary Materials

The following supporting information can be downloaded at: https://www.mdpi.com/article/10.3390/biom16101415/s1. Figure S1: Validation of the PAD2 knockout mouse model and tissue-specific PAD2 expression; Figure S2: PAD2, β–catenin, and global protein citrullination are differentially regulated during DSS-induced colitis; Figure S3: Additional characterization of microbial diversity and male antibiotic treatment; Figure S4: Additional characterization of epithelial proliferation, differentiation, and β-catenin regulation following PAD2 deficiency; Table S1: Terminal collection day and colon length in the longitudinal DSS survival cohort; Table S2: Pathway abundance analysis; Numerical Source Data: Raw numerical data underlying the graphs presented in the manuscript and supplementary materials; Supplementary Western blots: Full, uncropped immunoblot images underlying the reported immunoblot analyses.

## Abbreviations

The following abbreviations are used in this manuscript:

ABX	Antibiotics
CK1	Casein kinase 1
DAI	Disease activity index
DSS	Dextran sodium sulfate
GSK3β	Glycogen synthase Kinase 3-β
IBD	Inflammatory bowel disease
KO	Knockout
PAD	Peptidylarginine deiminase
PBS	Phosphate-buffered saline
p-GSK3β	Phospho-Glycogen synthase Kinase 3-β
PCoA	Principal coordinates analysis
PERMANOVA	Permutational Multivariate Analysis of Variance
PERMDISP	Permutational Multivariate Analysis of Dispersion
rhPAD2	Recombinant human PAD2
WT	Wild-type

## Appendix A

**Table A1:** Antibodies and Detection Reagents Used for Biochemical and Immunofluorescent Analysis.

Target	Species	Company/Catalog Number
Murine		
PAD2	Rabbit mAb	Abcam, Waltham, CA, USA/322197
β-Catenin (Total)	Rabbit mAb	Cell Signaling Technology, Danvers, MA, USA/8480
Non-phospho (active) β-Catenin	Rabbit mAb	Cell Signaling Technology/8814
p-β-Catenin	Rabbit pAb	Cell Signaling Technology/9561
Cd326 (EPCAM), APC	Rat-anti-mouse mAb	Thermo Scientific Waltham, MA, USA/17-5791-80
Casein Kinase 1(CK1)	Rabbit pAb	Cell Signaling Technology/2655
Phospho-Glycogen synthase kinase-3β (p-GSK3β)	Rabbit mAb	Cell Signaling Technology/5558
Glycogen synthase kinase-3 (GSK3β)	Rabbit mAb	Cell Signaling Technology/12456
DVL3	Rabbit pAb	Cell Signaling Technology/3218
αE-Catenin	Rabbit pAb	Cell Signaling Technology/3236
BrdU	Rat mAb	Abcam/6326
E-Cadherin	Rabbit pAb	BiCell Scientific, Maryland Heights, MO, USA/00101
Pan-Citrulline	Mouse mAb	Cayman, Ann Arbor, MI, USA/30773
PAD4	Rabbit mAb	Abcam/ab214810
p21	Rabbit mAb	Abcam/188224
Cyclin D1	Rabbit mAb	Cell Signaling Technology/55506
GAPDH	Rabbit mAb	Cell Signaling Technology/2118
IgG	Rabbit pAb	Millipore, Burlington, MA, USA/12-370
Fecal		
PAD2	Rabbit pAb	ProteinTech, Rosemont, IL, USA/12110-1-AP
Primary Detection Reagent		
Anti-Citrulline (modified)	Human mAb	Millipore, Burlington, MA, USA/MABS487
Secondary		
Anti-Rabbit HRP	Goat	Cell Signaling Technology/7074
Anti-Rabbit 488	Goat	Invitrogen, Waltham, MA, USA/11008
Anti-Rat 546	Goat	Invitrogen/11081
Anti-Rabbit 647	Goat	Invitrogen/21245
Anti-human IgG HRP	Goat mAb	Millipore/AP112P-CS216591
Anti-Mouse HRP	Goat	Cell Signaling Technology/91196

**Table A2:** PCR and qPCR Primer Sequences.

Gene	Type	Sequence (5′-3′)	Catalog
Murine targets			
*Genomic DNA*			
Padi2-gDNA (WT)	Forward	CTTATTCCTGGGAGCAGCAC	
	Reverse	CTCTCTCTCGGCTCACCTGT	
Padi2-gDNA (LacZ-Neo)	Forward	GATCGGCCATTGAACAAGAT	
	Reverse	GTAGCCGGATCAAGCGTATG	
RT-PCR			
*Myc*	Forward	TCG CTG CTG TCC TCC GAG TCC	OriGene, Rockville, MD, USA MP208493 (NM_010849)
	Reverse	GGT TTG CCT CTT CTC CAC AGA C	
*Axin2*	Forward	ATG GAG TCC CTC CTT ACC GCA T	OriGene, MP201191 (NM_015732)
	Reverse	GTT CCA CAG GCG TCA TCT CCT T	
*Ctnnb1* (β-*Catenin*)	Forward	GTT CGC CTT CAT TAT GGA CTG CC	OriGene, MP201814 (NM_007614)
	Reverse	ATA GCA CCC TGT TCC CGC AAA G	
*Lgr5*	Forward	AGA GCC TGA TAC CAT CTG CAA AC	OriGene, MP207434 (NM_010195)
	Reverse	TGA AGG TCG TCC ACA CTG TTG C	
*Sox9*	Forward	CAC ACG TCA AGC GAC CCA TGA A	OriGene, MP216226 (NM_011448)
	Reverse	TCT TCT CGC TCT CGT TCA GCA G	
*Klf4*	Forward	CTA TGC AGG CTG TGG CAA AAC C	OriGene, MP207225 (NM_010637)
	Reverse	TTG CGG TAG TGC CTG GTC AGT T	
*Ascl2*	Forward	TTT CCT GTG CCG CAC CAG AAC T	OriGene, MP200198 (NM_008554)
	Reverse	CAG CGA CTC CAG ACG AGG TGG	
*Cdh1* (*E*-*Cadherin*)	Forward	GGT CAT CAG TGT GCT CAC CTC T	OriGene, MP201510 (NM_009864)
	Reverse	GCT GTT GTG CTC AAG CCT TCA C	
*Rplp0*	Forward	GCT TCG TGT TCA CCA AGG AGG A	OriGene, MP200211 (NM_007475)
	Reverse	GTC CTA GAC CAG TGT TCT GAG C	
