# Supplementary material for: PAD2 Deficiency Reveals Sex-Divergent Colonic Homeostasis, β-Catenin Remodeling, and Antibiotic-Sensitive Phenotypes
Source: Biomolecules. 2026 Sep 30;16(10):1415. doi: 10.3390/biom16101415 (PMC13702572; doi:10.3390/biom16101415)

Supplementary Western Blot Source Data

A. Molecular weight reference

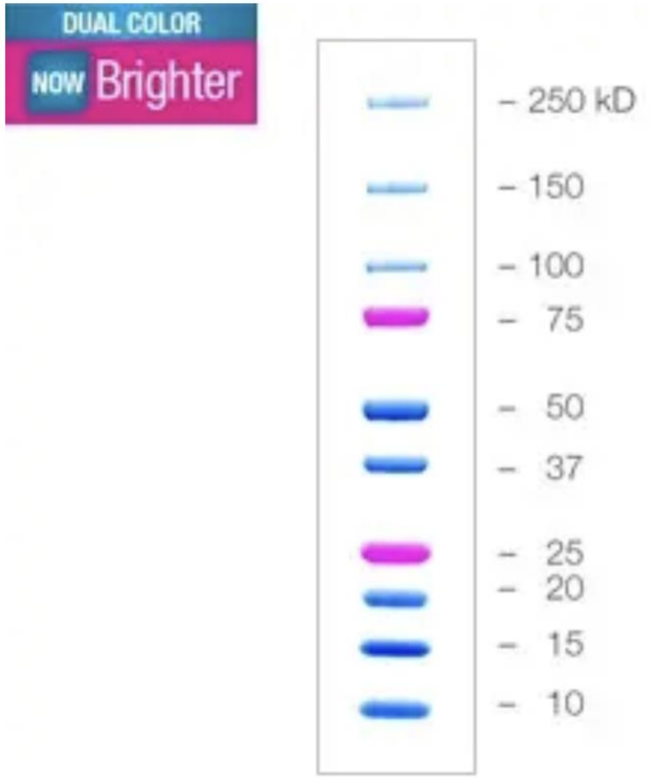

Note: An ‘X’ denotes a lane not included in the displayed figure panel and does not denote a splice. All displayed immunoblot strips contain contiguous lanes from the corresponding original image.

Figure 5A & 5B

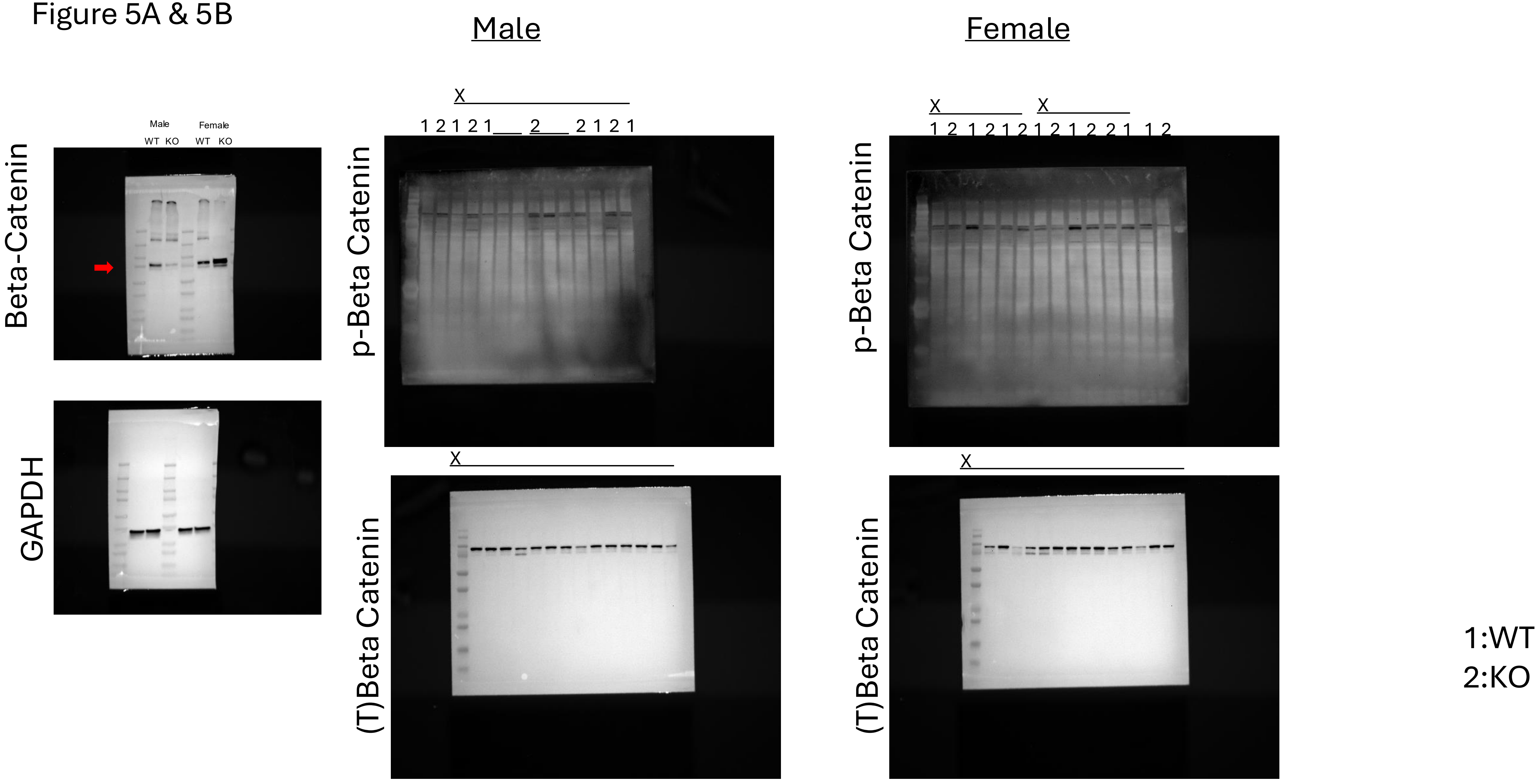
Male
Female
Beta-Catenin
Male
Female
WT KO WT KO
GAPDH
X
1 2 1 2 1 2 2 1 2 1
p-Beta Catenin
X
(T)Beta Catenin
X X
1 2 1 2 1 2 1 2 1 2 2 1 1 2
p-Beta Catenin
X
(T)Beta Catenin
1:WT
2:KO

Additional p-β-catenin and β-catenin samples

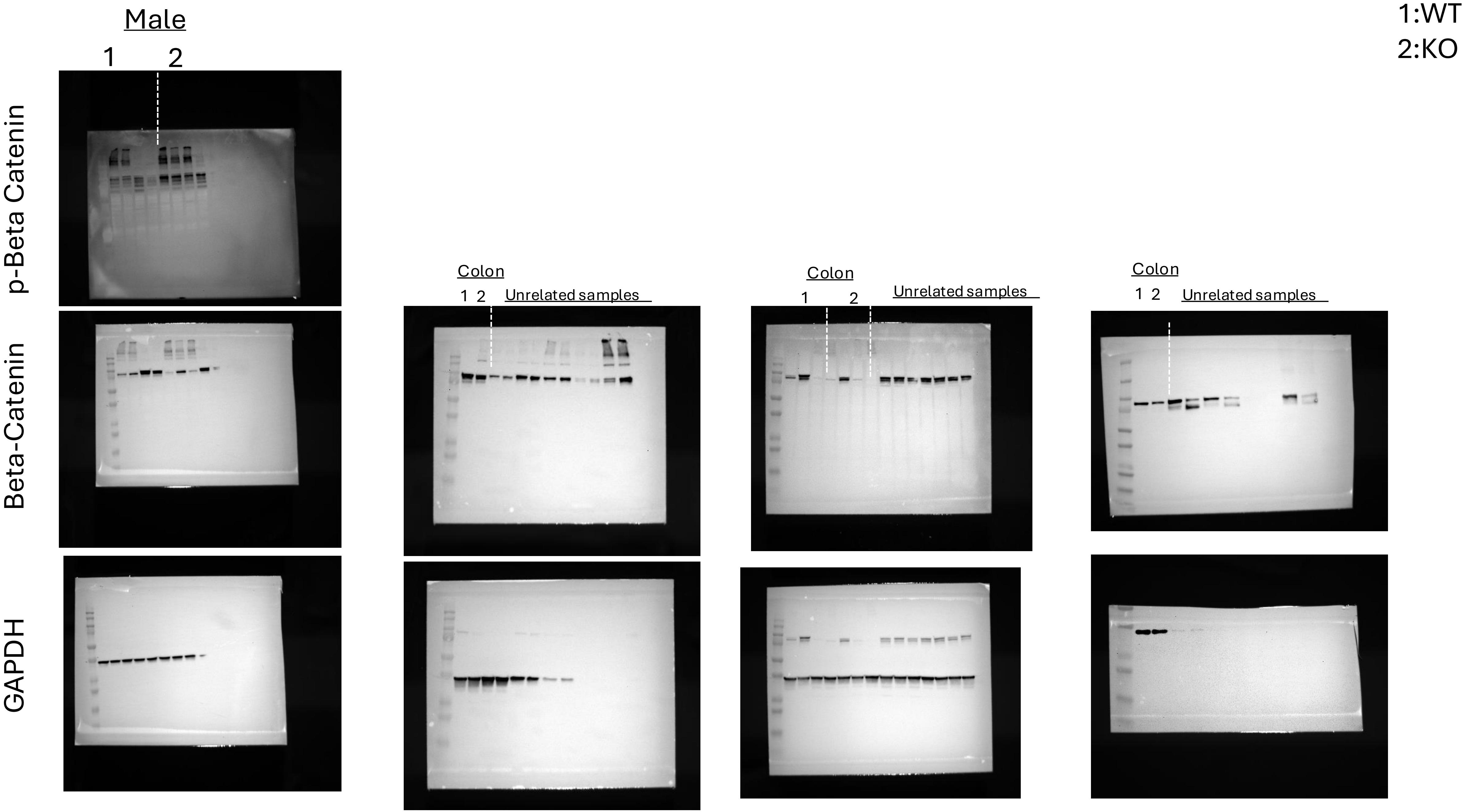

Additional β-catenin samples

Male

Unrelated organs — Colon 1 2

Colon 1 2 1 2 Unrelated samples

Beta-Catenin

GAPDH

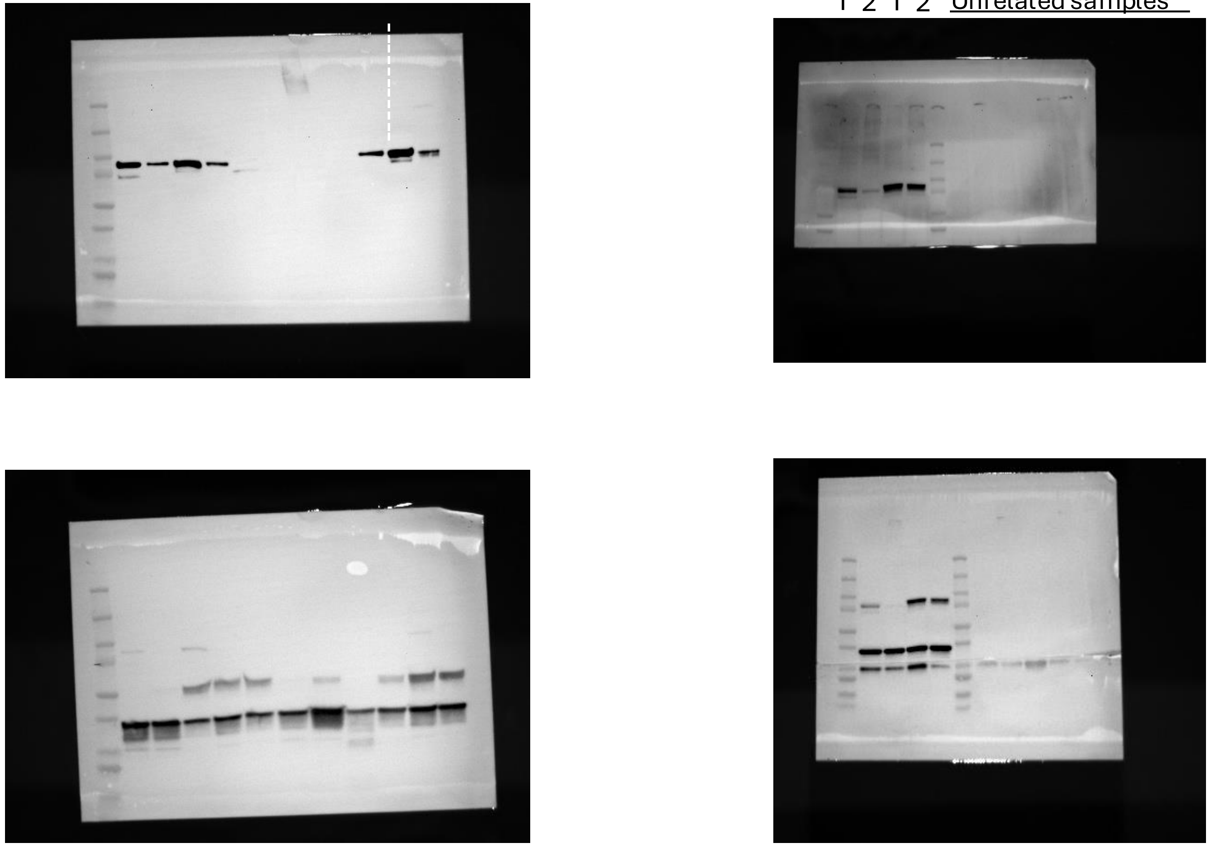

1:WT
2:KO

Additional β-catenin samples

Female

1:WT
2:KO

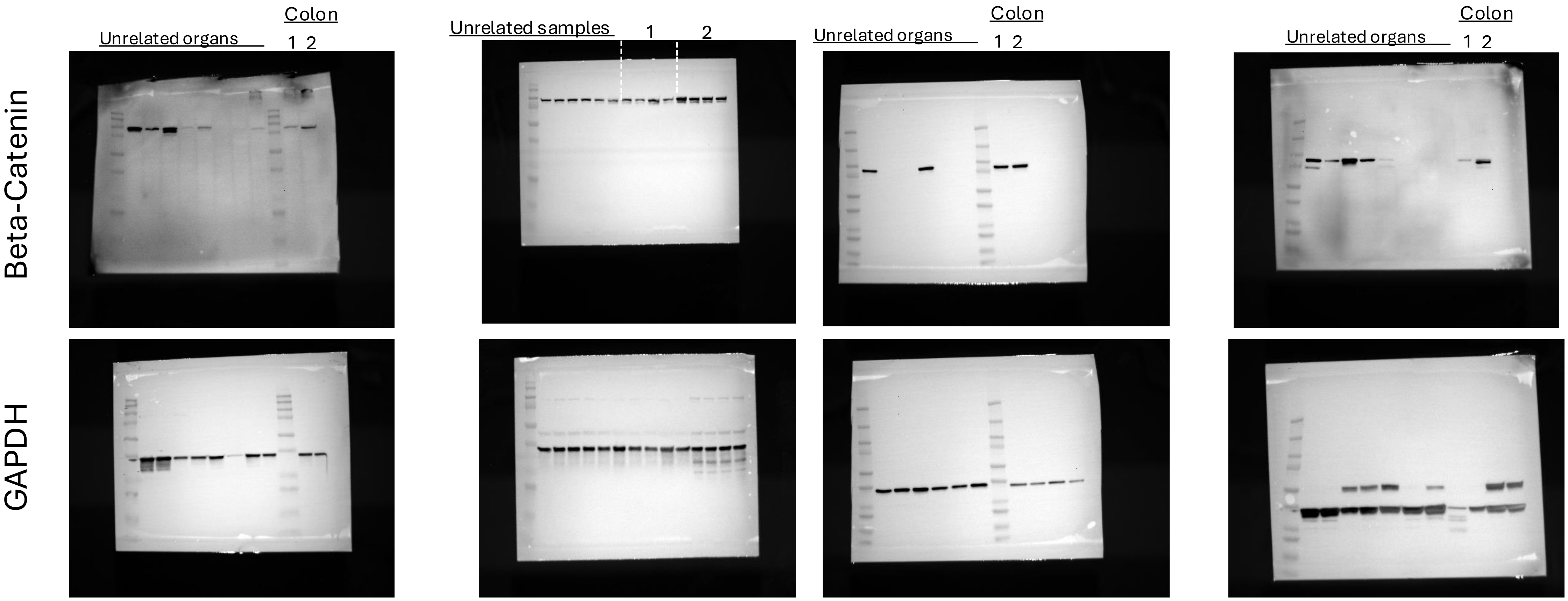

Figure 5C

1:WT
2:KO

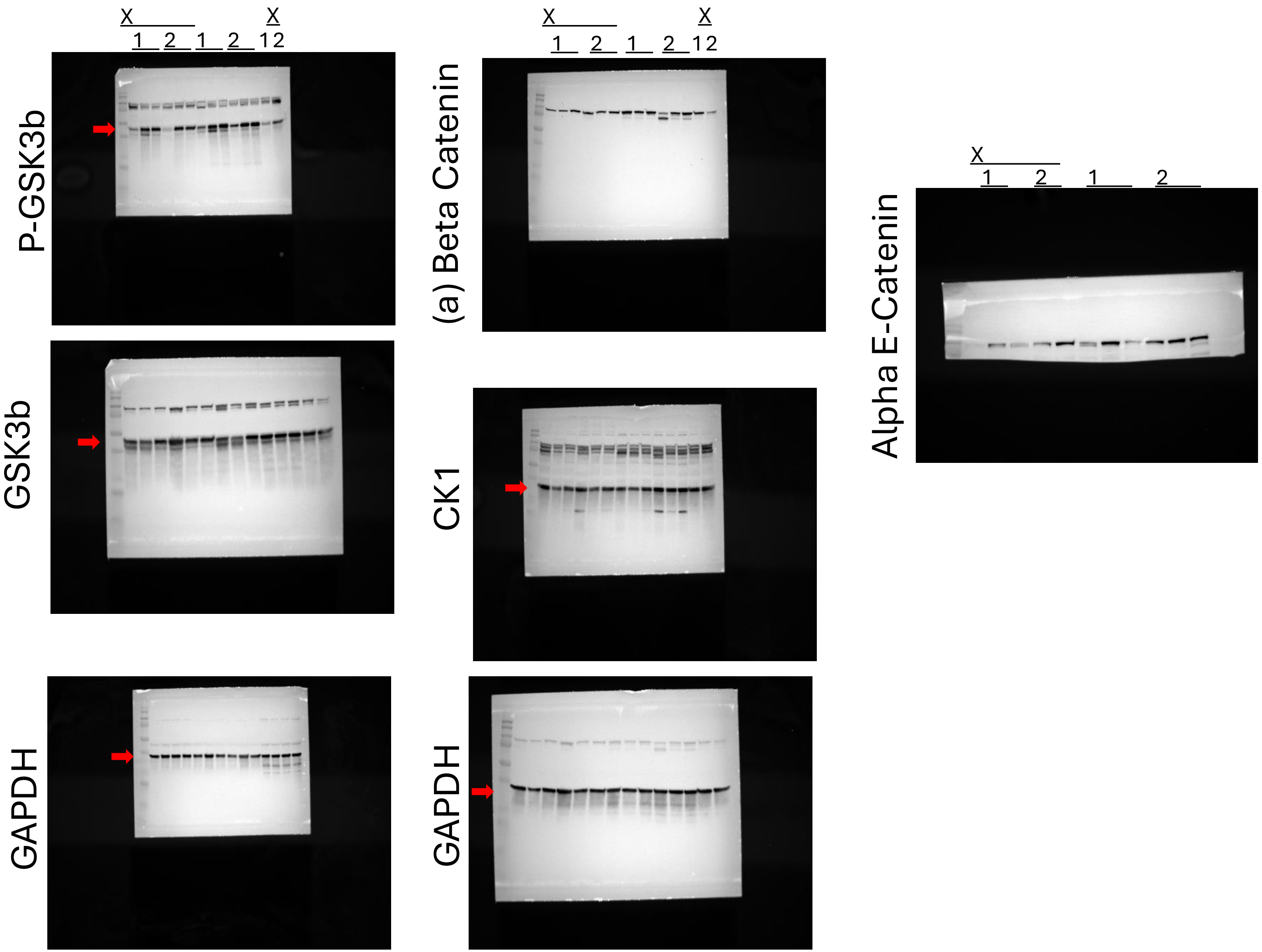

Figure 5D

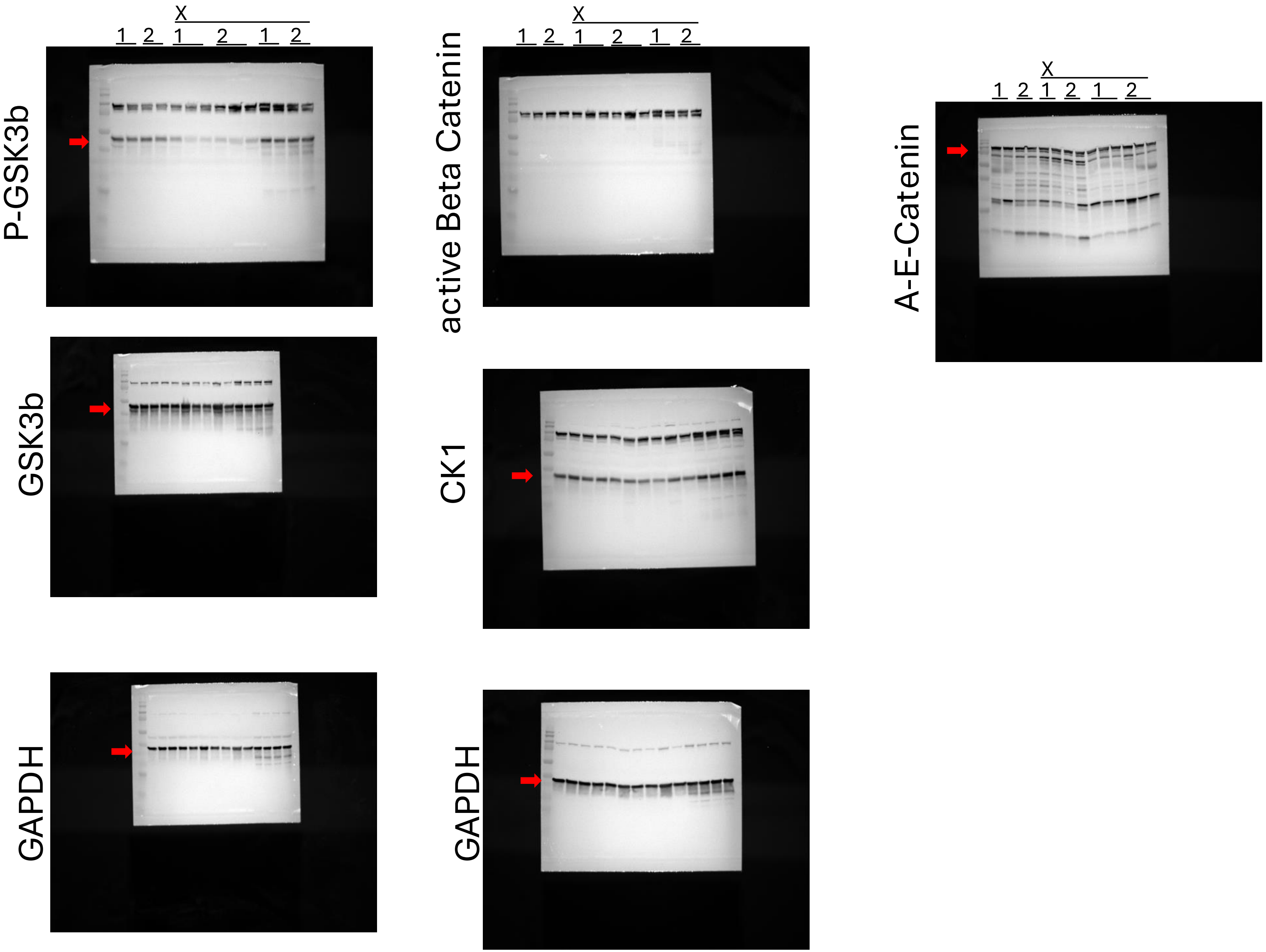

1:WT
2:KO

Figure 6A & 6C

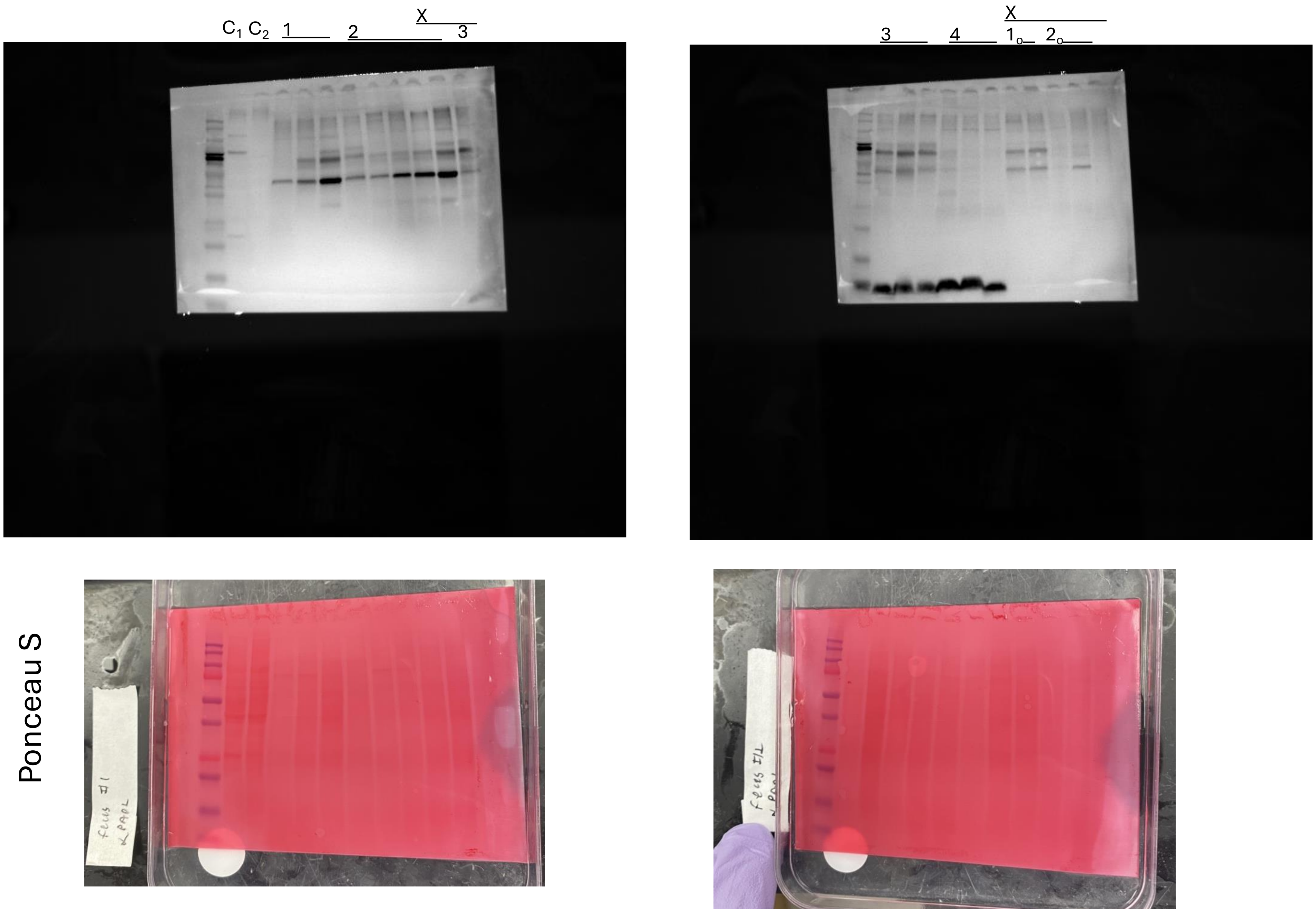

C_1: WT Colon
C_2:KO Colon
1:WT (Female)
2:KO (Female)
3:WT (Male)
4:KO (Male)
1_o: 48week WT Female
2_o: 48week KO Female

Figure 6I

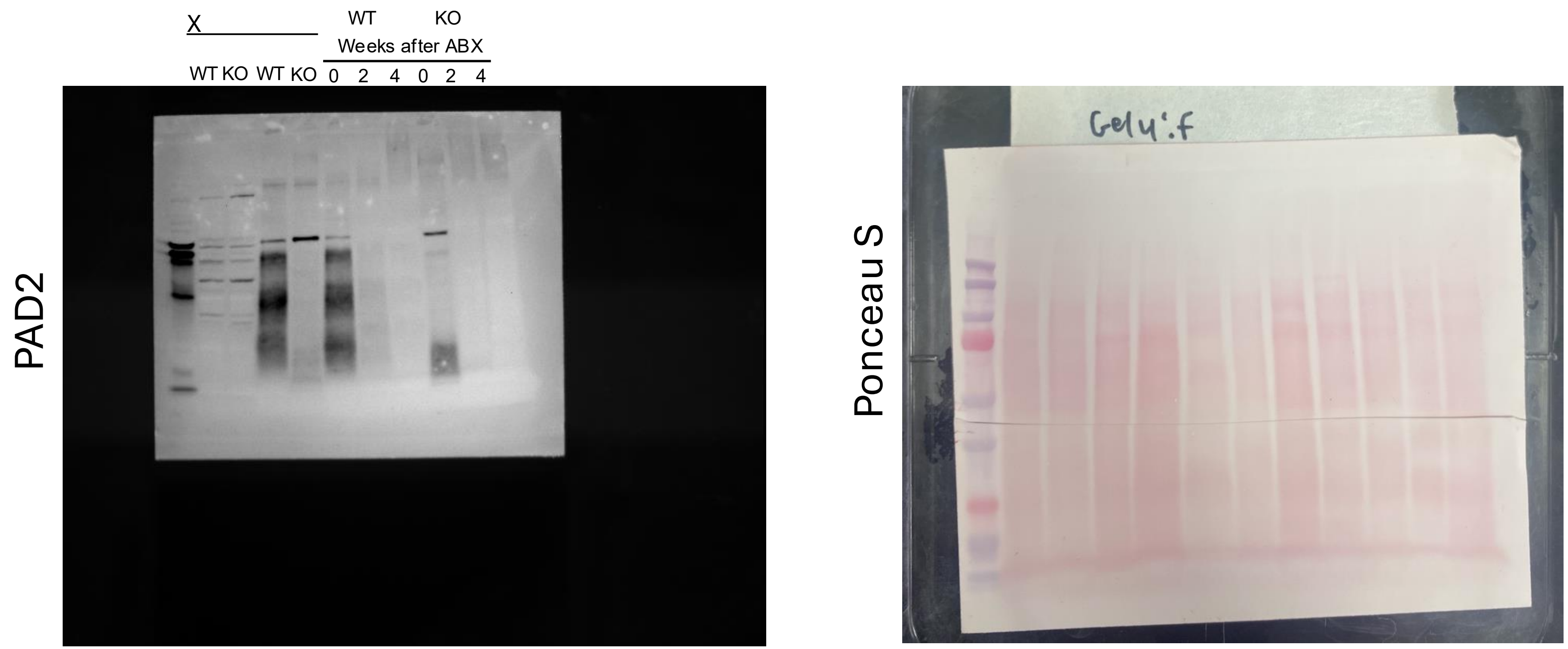

Figure 6K

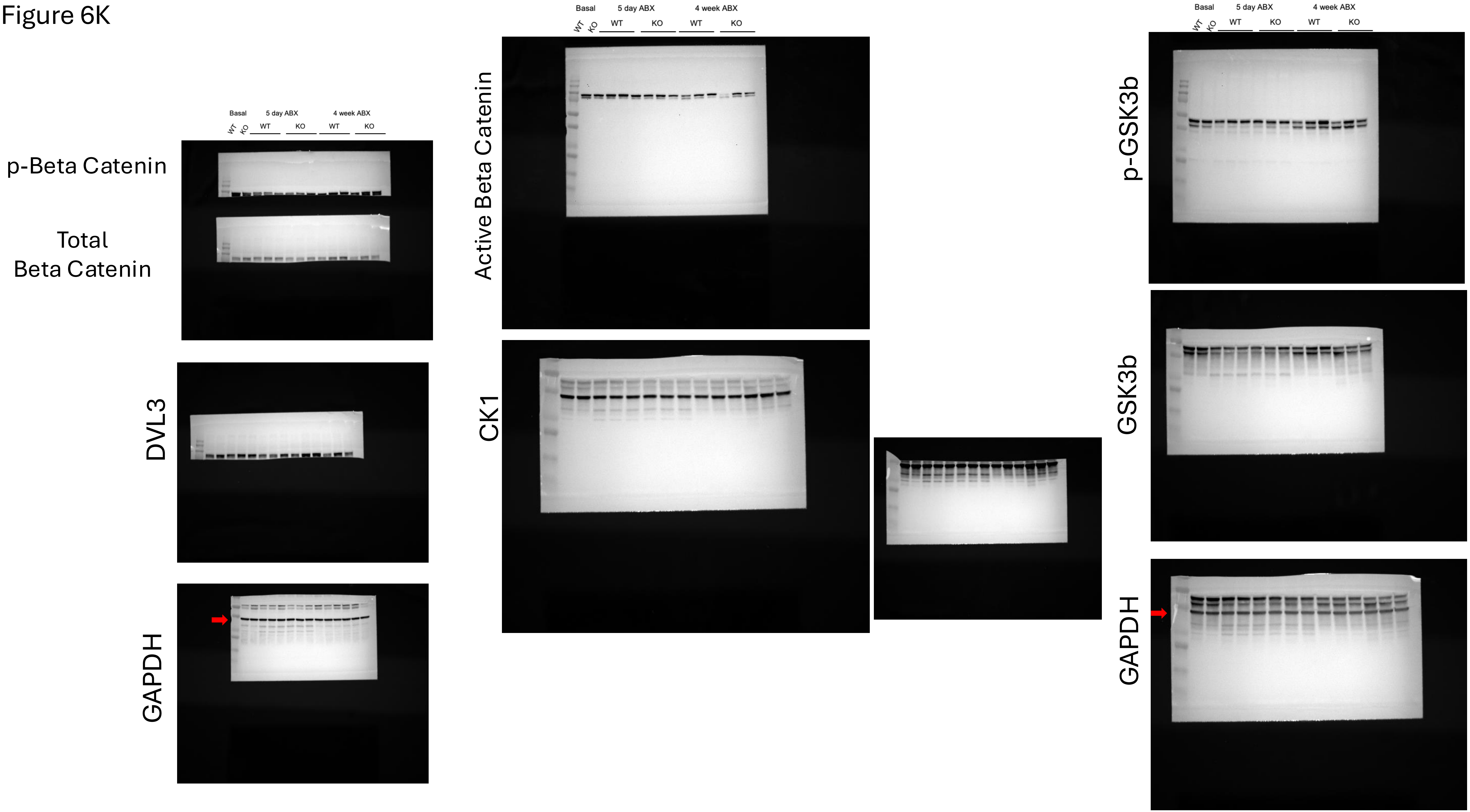
Basal
5 day ABX
4 week ABX
WT
KO
WT
KO
WT
KO
p-Beta Catenin
Total Beta Catenin
DVL3
GAPDH
Active Beta Catenin
CK1
p-GSK3b
GSK3b
GAPDH

Figure 7A

Serum

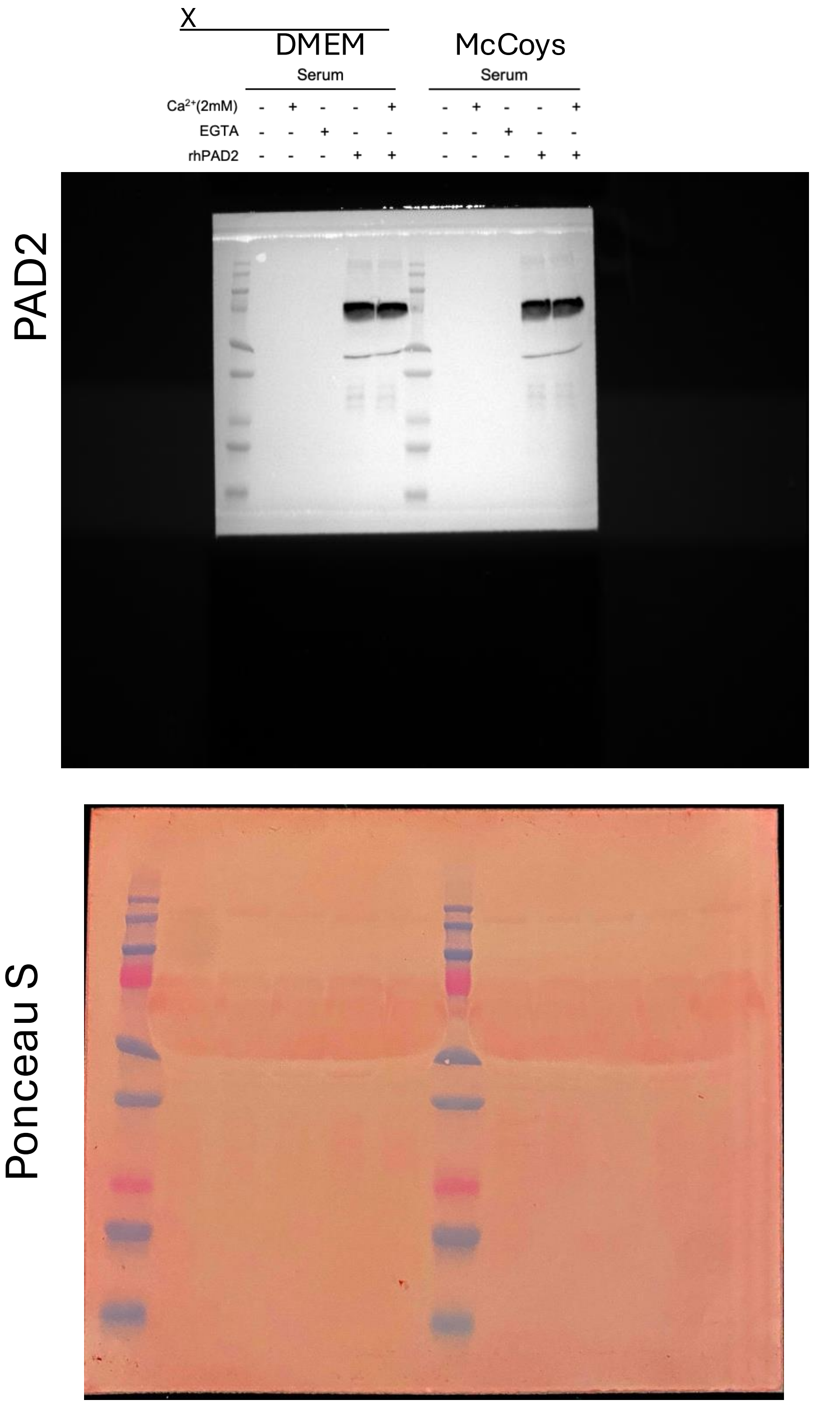

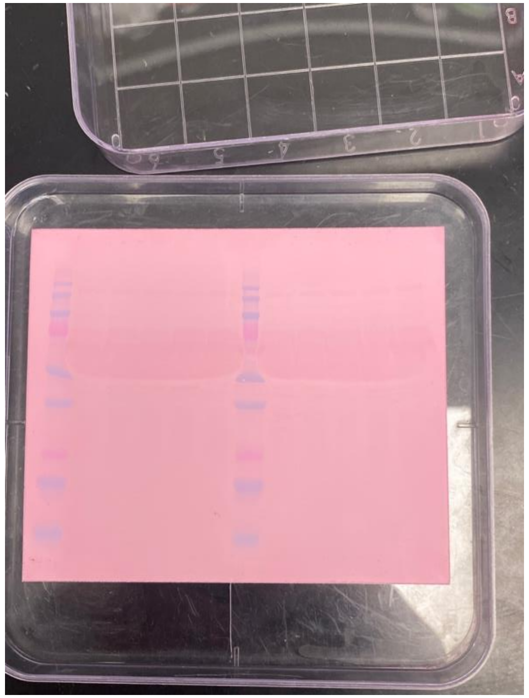

Figure 7B

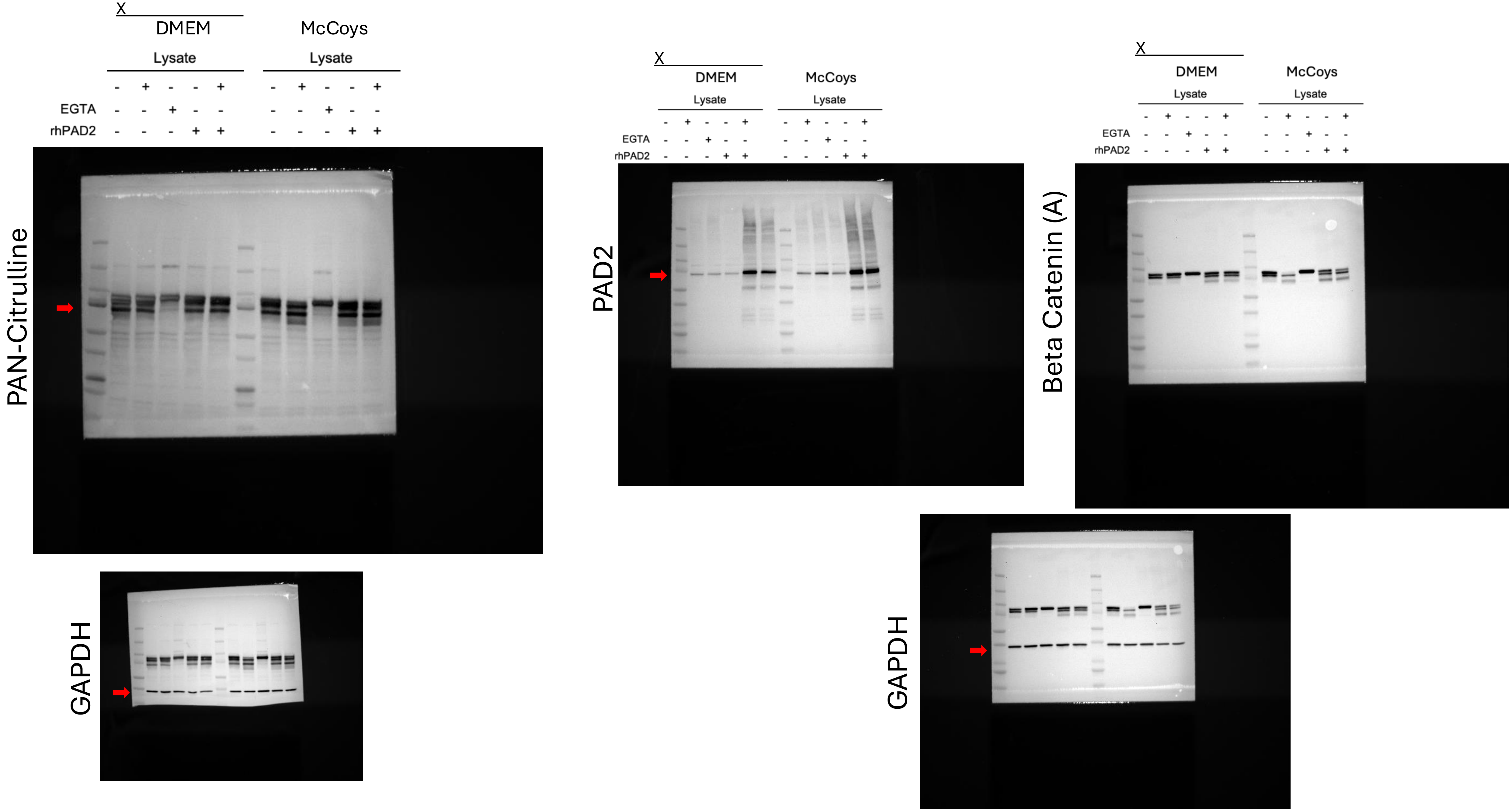

X
DMEM
McCoys
Lysate
Lysate
EGTA
rhPAD2
PAN-Citrulline
GAPDH
X
DMEM
McCoys
Lysate
Lysate
EGTA
rhPAD2
PAD2
X
DMEM
McCoys
Lysate
Lysate
EGTA
rhPAD2
Beta Catenin (A)
GAPDH

Figure S1C

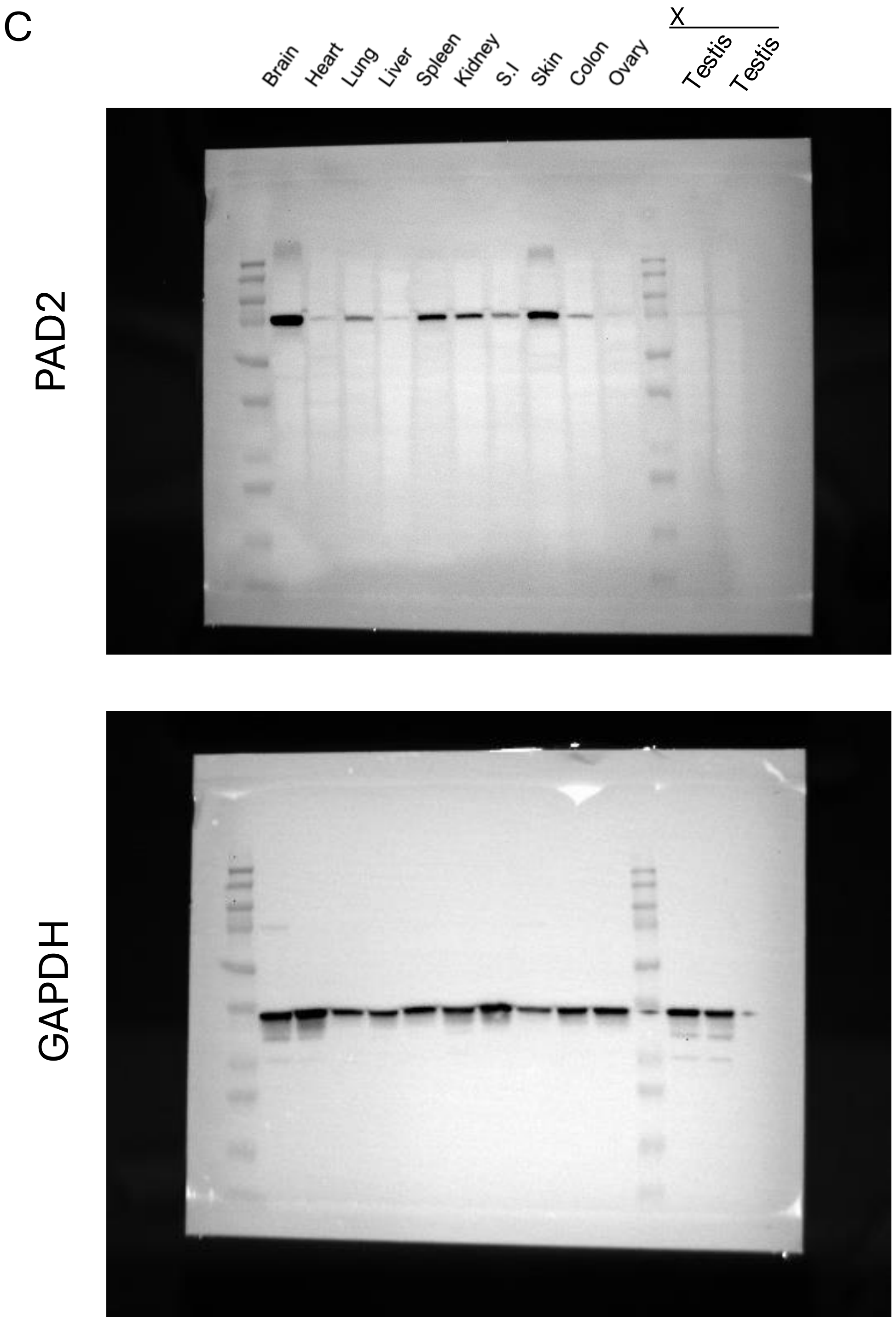
X
Brain
Heart
Lung
Liver
Spleen
Kidney
S.I
Skin
Colon
Ovary
Testis
Testis
PAD2
GAPDH

Additional source data for Figure S1C

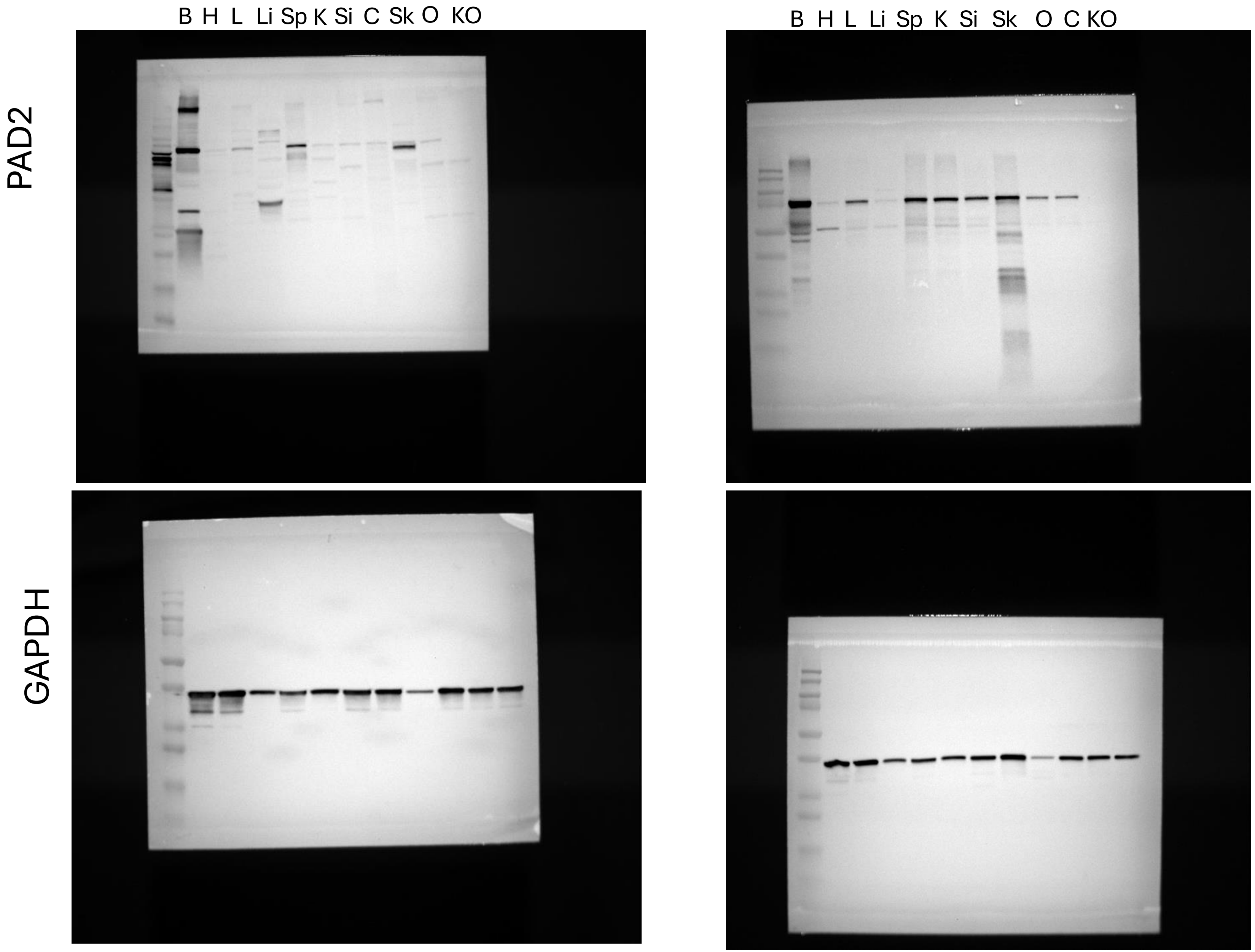

Brain: B
Heart: H
Lung: L
Liver: Li
Spleen: Sp
Kidney: K
Small Intestine: SI
Colon: C
Ovary: O
KO: Knock Out (colon)

Additional source data for Figure S1C

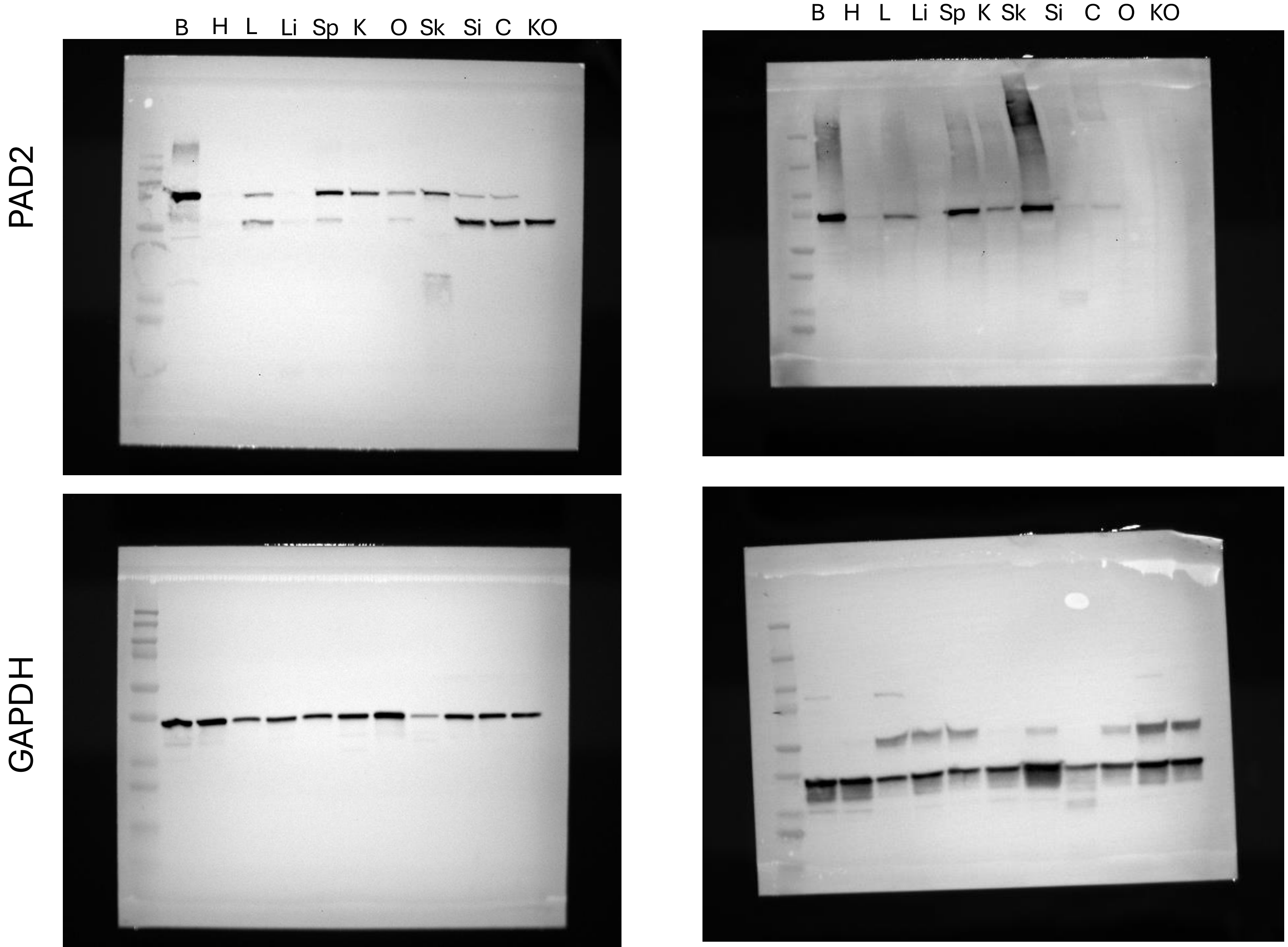

Brain: B
Heart: H
Lung: L
Liver: Li
Spleen: Sp
Kidney: K
Small Intestine: SI
Colon: C
Ovary: O
KO: Knock Out (colon)

*Note: This membrane was sequentially probed with additional antibodies unrelated to the present study prior to GAPDH detection. Additional immunoreactivity at approximately 50 kDa is therefore present on the source image and was not used for quantification.

Male

B H L Li Sp K Sk Si T C KO

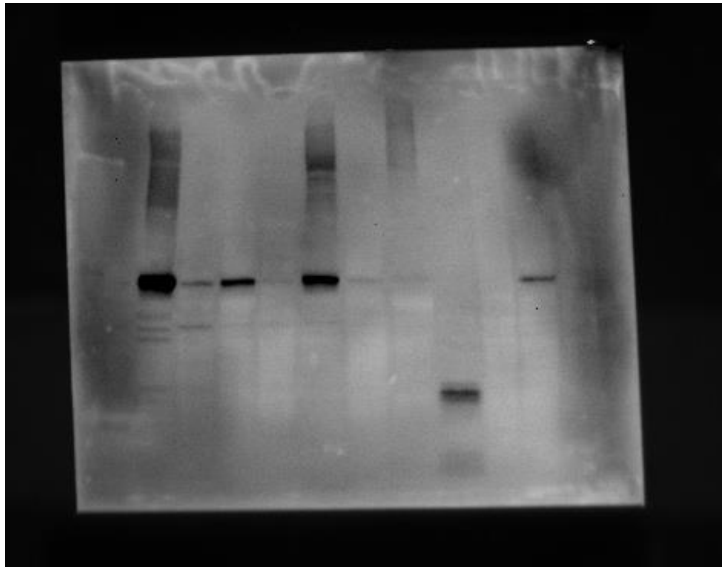

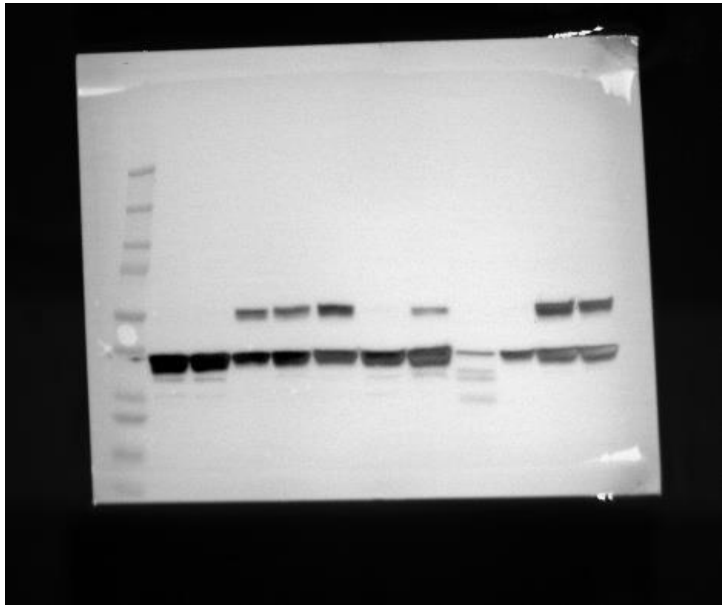

Brain: B
Heart: H
Lung: L
Liver: Li
Spleen: Sp
Kidney: K
Small Intestine: SI
Colon: C
Testi: T
KO: Knock Out (colon)

*Note: This membrane was sequentially probed with additional antibodies unrelated to the present study prior to GAPDH detection. Additional immunoreactivity at approximately 50 kDa is therefore present on the source image and was not used for quantification.

Figure S2 D & S2E

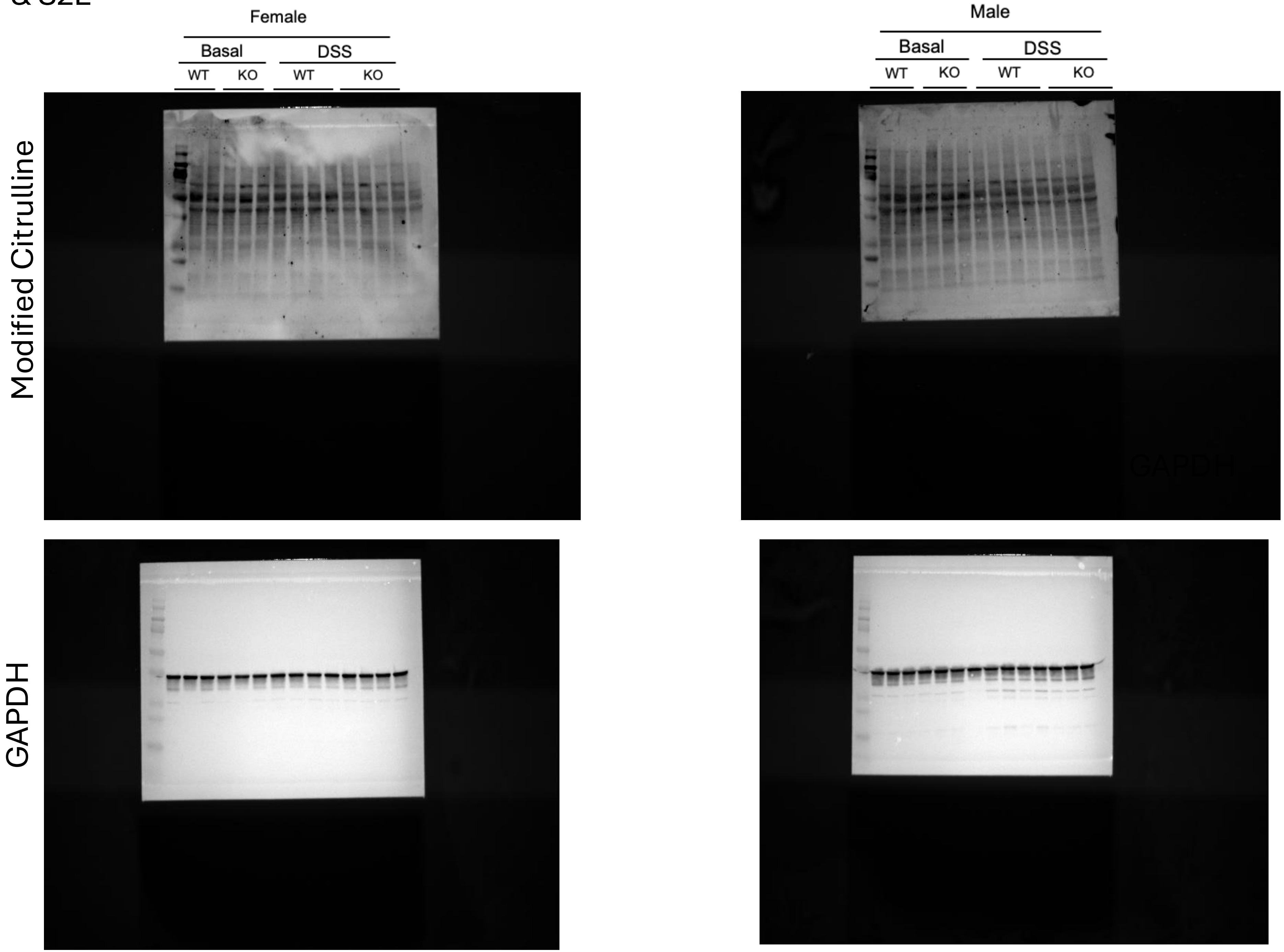

Figure S2F

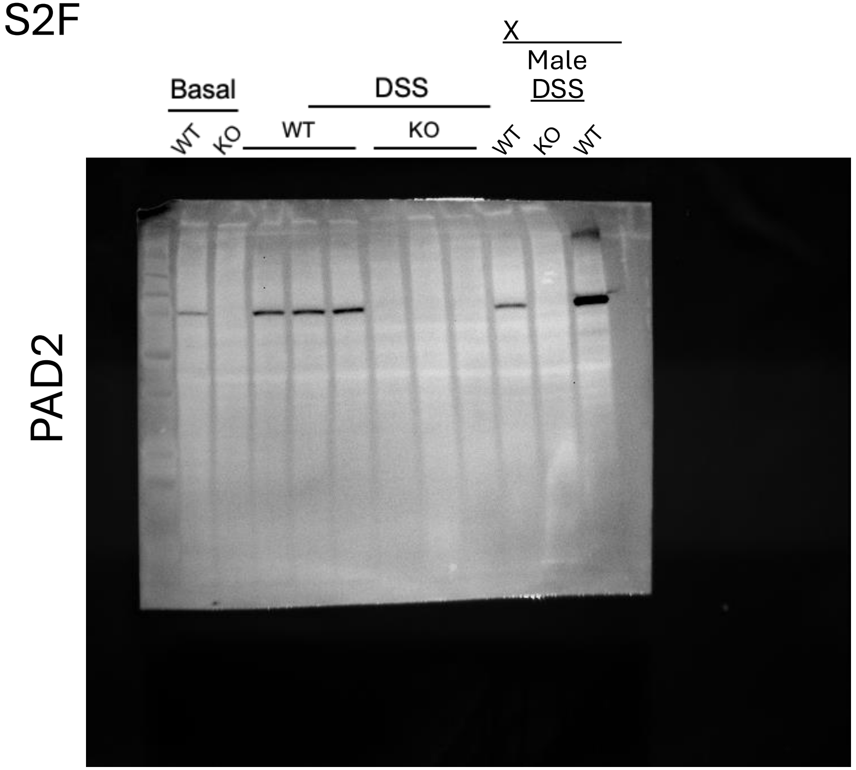
X
Male
DSS
Basal
DSS
WT
KO
WT
KO
WT
KO
WT
PAD2

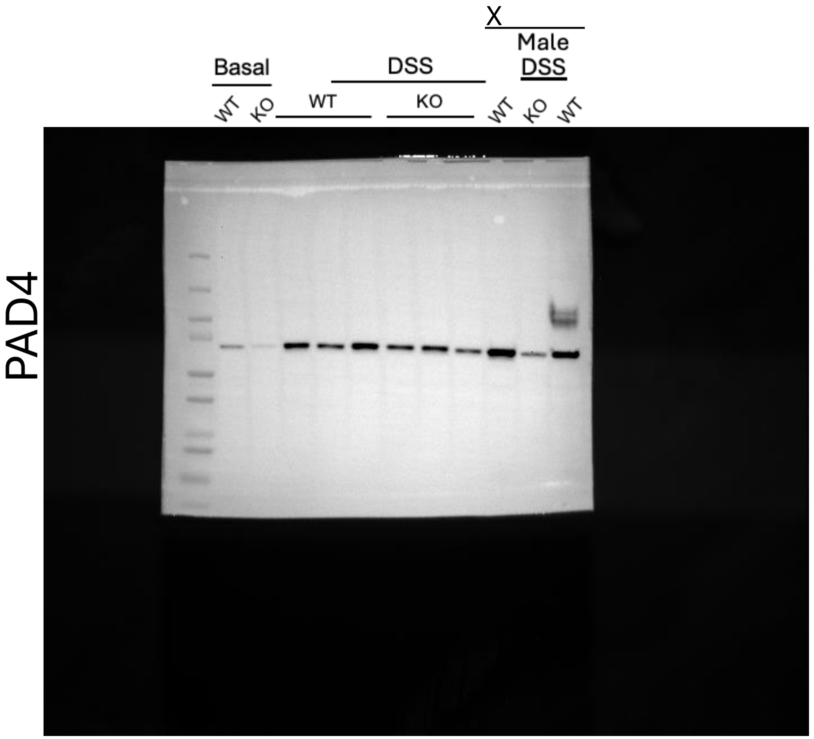
X
Male
DSS
Basal
DSS
WT
KO
WT
KO
WT
KO
WT
PAD4

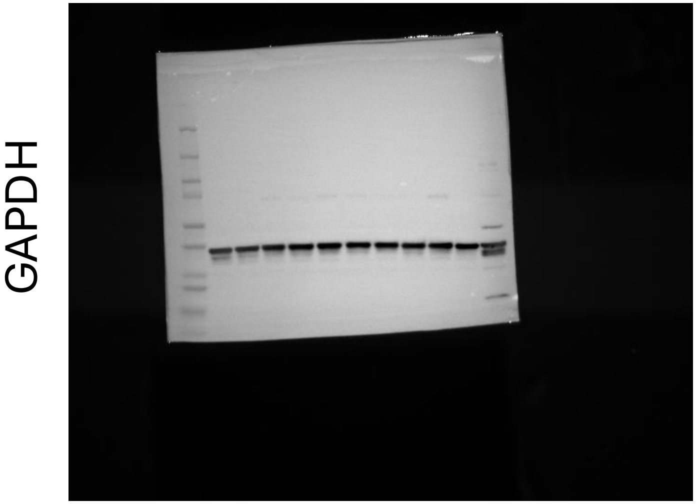
GAPDH

Figure S2I

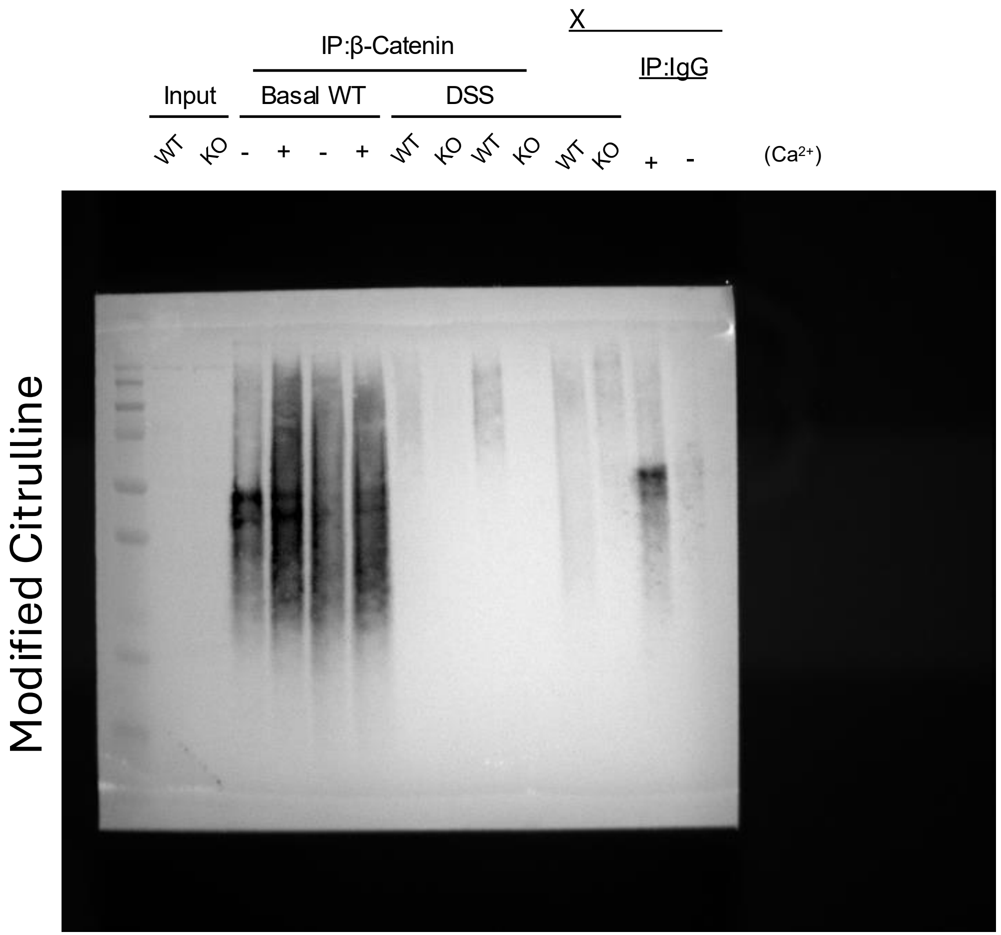
X
IP:β-Catenin
IP:IgG
Input
Basal WT
DSS
WT
KO
-
+
-
+
WT
KO
WT
KO
WT
KO
+
-
(Ca2+)
Modified Citrulline

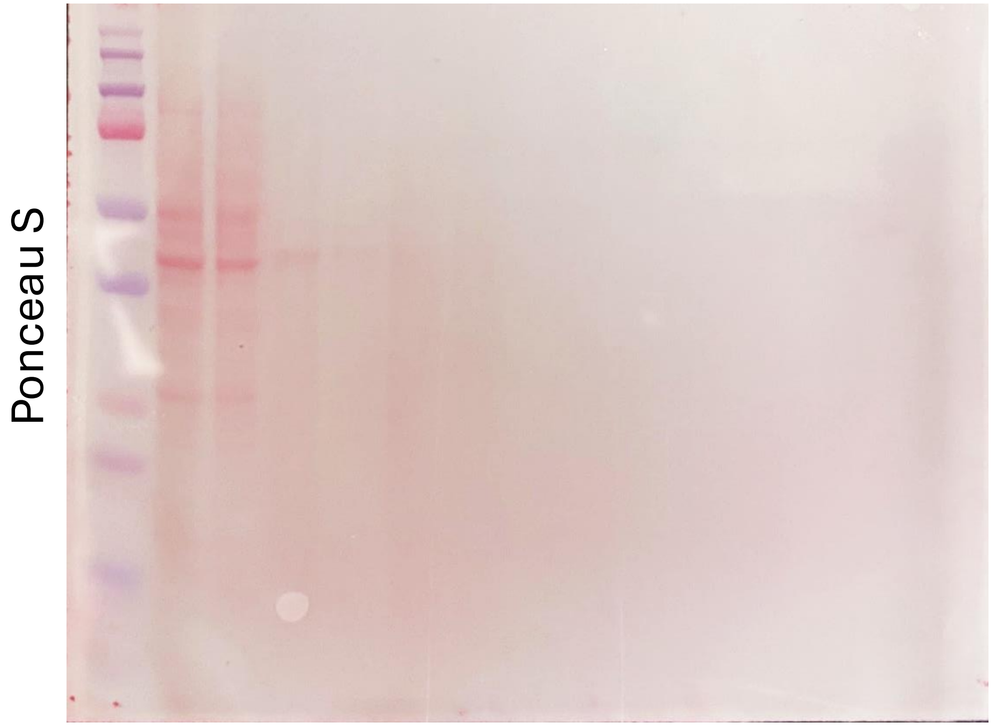
Ponceau S

Figure S2J

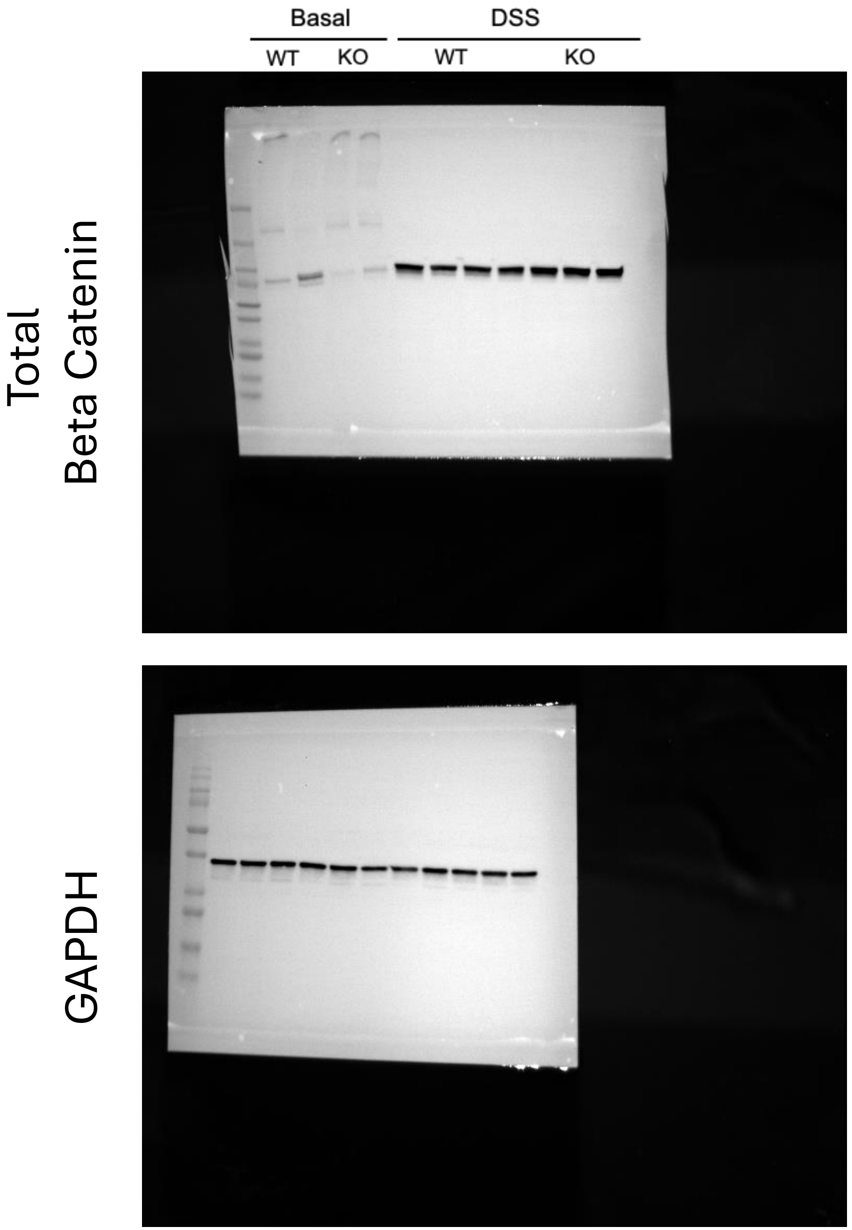

Figure S4C & S4E

Female X

WT KO WT KO WT KO WT KO WT KO

Cyclin D1

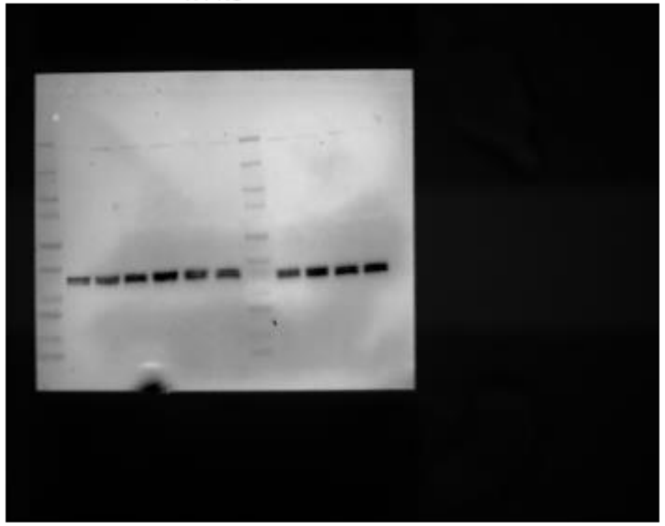

GAPDH

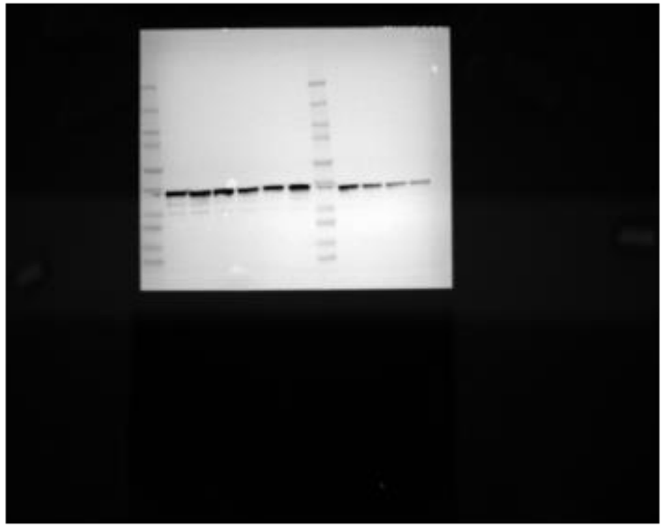

X

Female Male Female

WT KO WT KO WT KO WT WT KO KO KO

p21

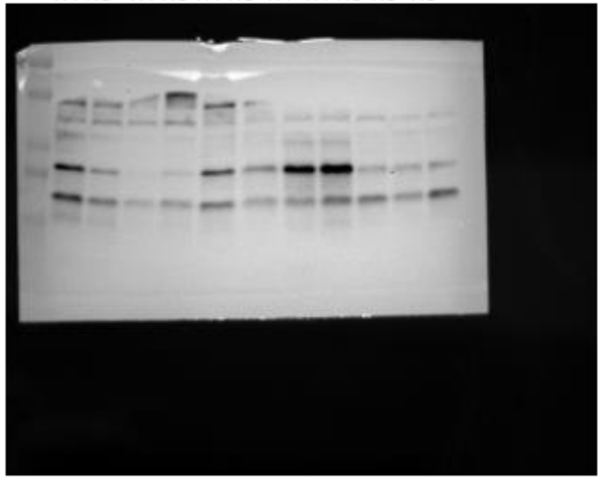

GAPDH

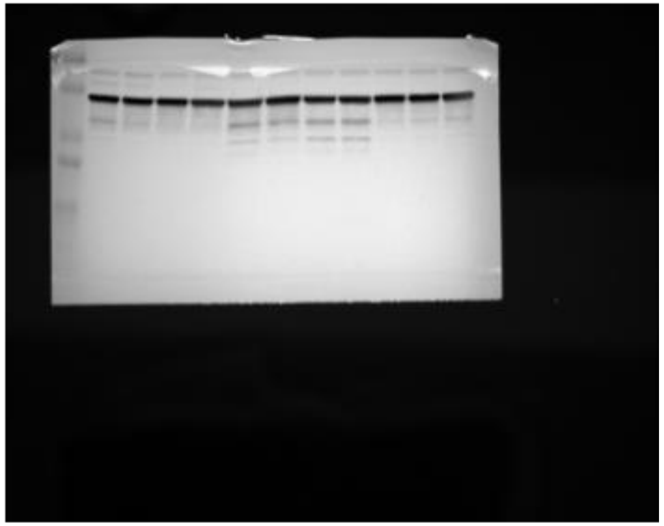

Figure S4G

*Same as labeled in S4G

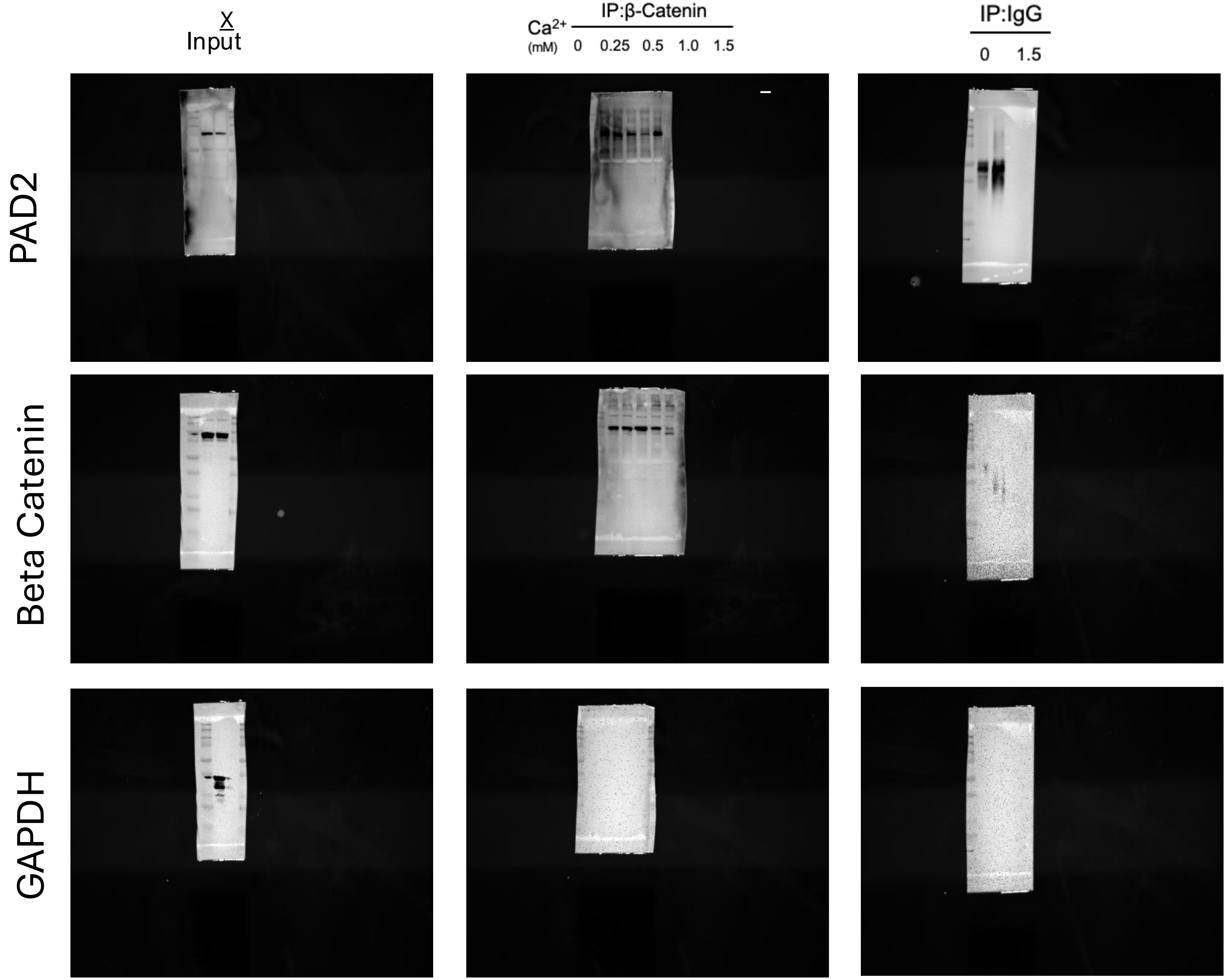

Supplement: Supplementary file 1 [file biomolecules-16-01415-s001.zip › SupplementaryWesternBlots.pdf]
