# Supplementary material for: PAD2 Deficiency Reveals Sex-Divergent Colonic Homeostasis, β-Catenin Remodeling, and Antibiotic-Sensitive Phenotypes
Source: Biomolecules. 2026 Sep 30;16(10):1415. doi: 10.3390/biom16101415 (PMC13702572; doi:10.3390/biom16101415)
Supplement: Supplementary file 1 [file biomolecules-16-01415-s001.zip › Supporting_Materials Figure S1-S4.pdf]

Supporting Materials

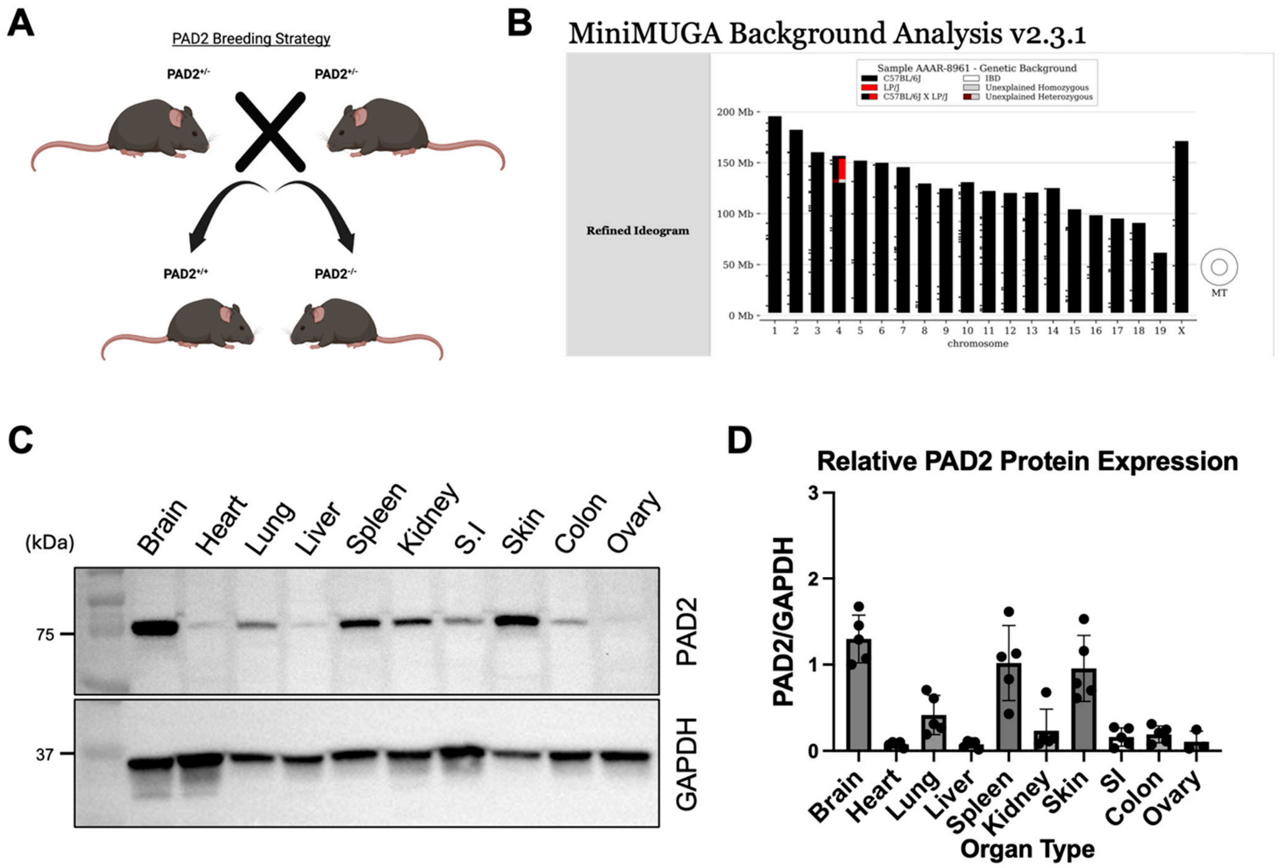

Figure S1. Validation of the PAD2 knockout mouse model and tissue-specific PAD2 expression. (**A**) Breeding strategy used to generate wild-type (WT) and PAD2 knockout (KO) littermate mice for all experiments. (**B**) Representative MiniMUGA genetic strain background analysis performed on heterozygous breeders following six generations of backcrossing to WT C57BL/6J (TransnetYX). (**C**) Representative immunoblot of PAD2 protein expression in brain, heart, lung, liver, spleen, kidney, small intestine (SI), skin, colon, and ovary from WT mice. (**D**) Descriptive densitometric quantification of PAD2 protein abundance normalized to GAPDH across tissues (n = 5 biological replicates per tissue). Densitometry is presented as mean ± SEM.

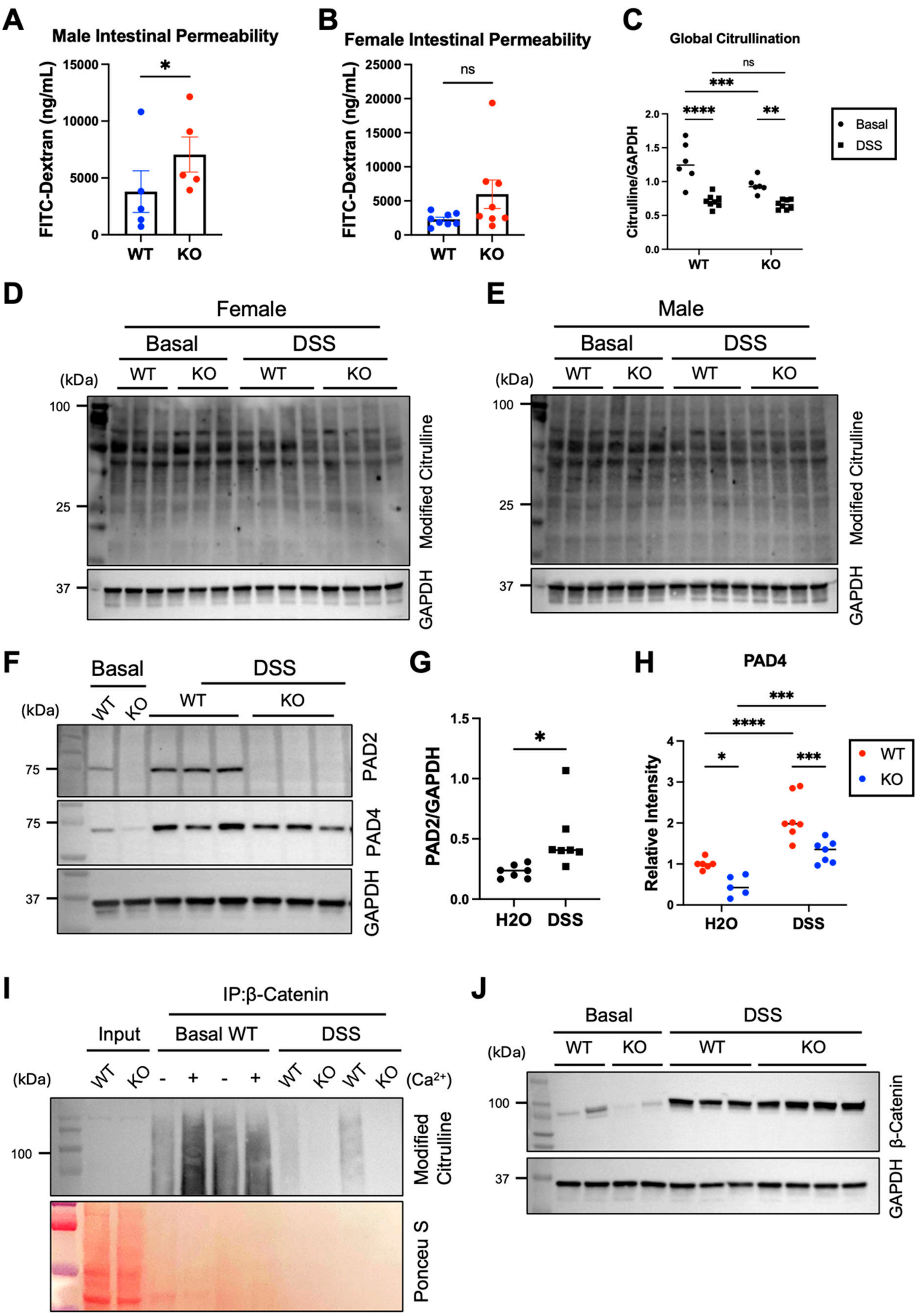
A
Male Intestinal Permeability
FITC-Dextran (ng/mL)
WT
KO
B
Female Intestinal Permeability
FITC-Dextran (ng/mL)
ns
WT
KO
C
Global Citrullination
Citrulline/GAPDH
ns
Basal
DSS
WT
KO
D
Female
Basal
DSS
WT
KO
(kDa)
Modified Citrulline
GAPDH
E
Male
Basal
DSS
WT
KO
(kDa)
Modified Citrulline
GAPDH
F
Basal
DSS
(kDa)
WT
KO
PAD2
PAD4
GAPDH
G
PAD2/GAPDH
H2O
DSS
H
PAD4
Relative Intensity
WT
KO
H2O
DSS
I
IP:β-Catenin
Input
Basal WT
DSS
(kDa)
WT
KO
(Ca2+)
Modified Citrulline
Ponceu S
J
Basal
DSS
(kDa)
WT
KO
β-Catenin
GAPDH

Figure S2. PAD2, β–catenin, and global protein citrullination are differentially regulated during DSS-induced colitis. (**A, B**) Intestinal permeability assessed by FITC-dextran following 7 days of 3% DSS exposure in male (A) and female (B) WT and PAD2 KO mice (male, n = 5/genotype; female, *n* = 8/genotype). **(C)** Combined densitometric quantification of modified citrulline normalized to GAPDH from male and female mice. Individual data points represent one mouse (basal WT and KO groups, *n* = 6 mice per genotype; DSS-treated WT and KO groups, *n* = 8 mice per genotype). (**D, E**) Representative immunoblots of modified citrulline in proximal colon lysates from female (D) and male (E) WT and PAD2 KO mice under basal conditions and following 7 days of 3% DSS exposure. (**F**) Representative immunoblots of PAD2, PAD4, and GAPDH from female WT and PAD2 KO proximal colon lysates under basal conditions and following 7 days of 3% DSS exposure. (**G**) Combined densitometric quantification of PAD2 normalized to GAPDH from male and female WT mice under basal conditions and following DSS exposure. (**H**) Combined densitometric quantification of PAD4 normalized to GAPDH from male and female WT and PAD2 KO mice under basal conditions and following DSS exposure (WT basal, *n* = 6; WT DSS, *n* = 7; KO basal, *n* = 5; KO DSS, *n* = 7). (**I**) Representative immunoblot of modified citrulline following β-catenin immunoprecipitation. Samples include input WT and KO total colon lysates, WT lysates incubated in the absence or presence of 1.5 mM $CaCl_2$, and WT and KO colons following 7 days of 3% dextran sulfate sodium (DSS) exposure. Total protein staining (Ponceau S) served as the loading control. (**J**) Representative immunoblot of total β-catenin in proximal colon lysates from male WT and PAD2 KO mice under basal conditions and following 7 days of 3% DSS exposure. Data in A and B are presented as mean ± SEM, with individual biological replicates shown. Data in C, G, and H are presented as individual biological replicates with the mean indicated. Intestinal permeability and PAD2 densitometry were analyzed using two-sided Welch's *t* tests. Modified citrulline and PAD4 densitometry were analyzed using two-way ANOVA followed by Šídák-adjusted planned comparisons. * $P < .05$; ** $P < .01$; *** $P < .001$; **** $P < .0001$.

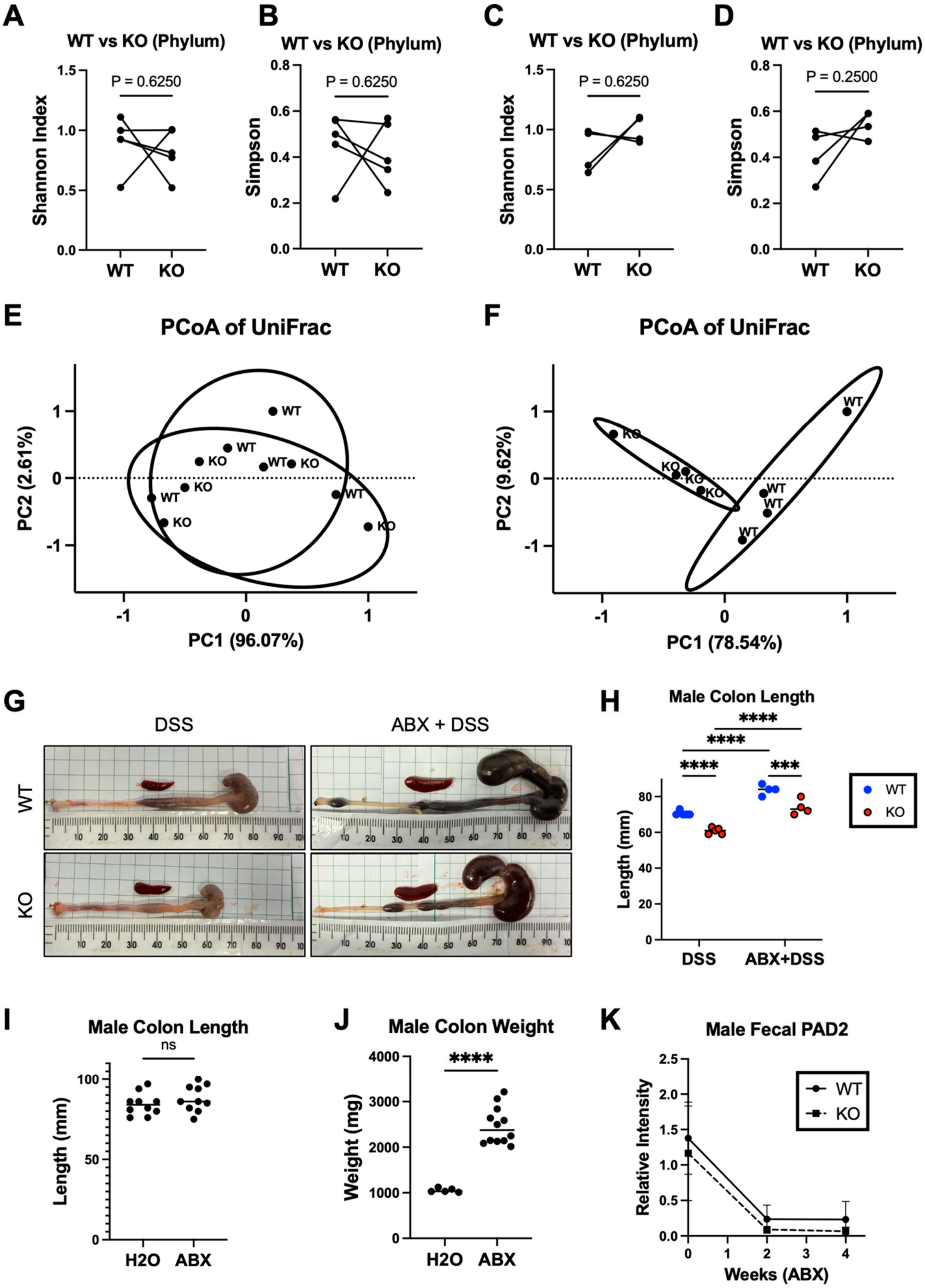
A
WT vs KO (Phylum)
P = 0.6250
Shannon Index
WT
KO
B
WT vs KO (Phylum)
P = 0.6250
Simpson
C
WT vs KO (Phylum)
P = 0.6250
Shannon Index
D
WT vs KO (Phylum)
P = 0.2500
Simpson
E
PCoA of UniFrac
PC2 (2.61%)
PC1 (96.07%)
F
PCoA of UniFrac
PC2 (9.62%)
PC1 (78.54%)
G
DSS
ABX + DSS
WT
KO
H
Male Colon Length
Length (mm)
DSS
ABX+DSS
I
Male Colon Length
ns
Length (mm)
H2O
ABX
J
Male Colon Weight
Weight (mg)
H2O
ABX
K
Male Fecal PAD2
Relative Intensity
Weeks (ABX)

Figure S3. Additional characterization of microbial diversity and male antibiotic treatment. (**A-D**) Shannon diversity index and Simpson diversity index of male (A, B) and female (C, D) fecal microbiota at baseline. Individual biological replicates are shown, with lines connecting matched WT and PAD2 KO littermates. (**E, F**) Principal coordinates analysis (*P*CoA) of male (E; Pseudo-F = 0.18864, P = 0.916) and female (F; Pseudo-F = 7.76303, P = 0.026) microbial communities at the genus level using weighted UniFrac distances and PERMANOVA with 999 permutations. (**G**) Representative gross colon images from male WT and PAD2 KO mice following 7 days of 2.5% DSS exposure in the presence or absence of 4 weeks of pretreatment with broad-spectrum antibiotics (ABX). (**H**) Colon length measurements from male WT and PAD2 KO mice following 7 days of 2.5% DSS treatment with or without 4 weeks of ABX (WT + DSS, n = 5; KO + DSS, n = 5; WT + ABX + DSS, n = 4; KO + ABX + DSS, n = 4). These mice represent the same prolonged ABX experiment shown for females in Figure 4A, B. (**I**) Colon length measurements from male WT and PAD2 KO mice following 4 weeks of ABX treatment in the absence of DSS (n = 10). (**J**) Cecal-colon weight from male WT and PAD2 KO mice following 4 weeks of ABX treatment in the absence of DSS (Basal, n = 5; ABX, n = 12). (**K**) Longitudinal densitometric quantification of male fecal PAD2 immunoreactivity during ABX treatment (n = 11 mice). Data in H-J are presented as individual biological replicates with the mean indicated; K is presented as mean ± SEM. Alpha-diversity indices were analyzed using two-sided Wilcoxon matched-pairs signed-rank tests. Community composition (β-diversity) was evaluated using weighted UniFrac distances and PERMANOVA with 999 permutations. Colon length (H) was analyzed using two-way ANOVA followed by Šídák-adjusted planned comparisons. Longitudinal fecal PAD2 immunoreactivity was analyzed using mixed-effects model (REML) with Geisser-Greenhouse correction for repeated measures followed by Šídák-adjusted multiple comparisons. Comparisons between two groups were analyzed using two-sided Welch's t tests. * $P < .05$; ** P < .01; *** $P < .001$; **** P < .0001.

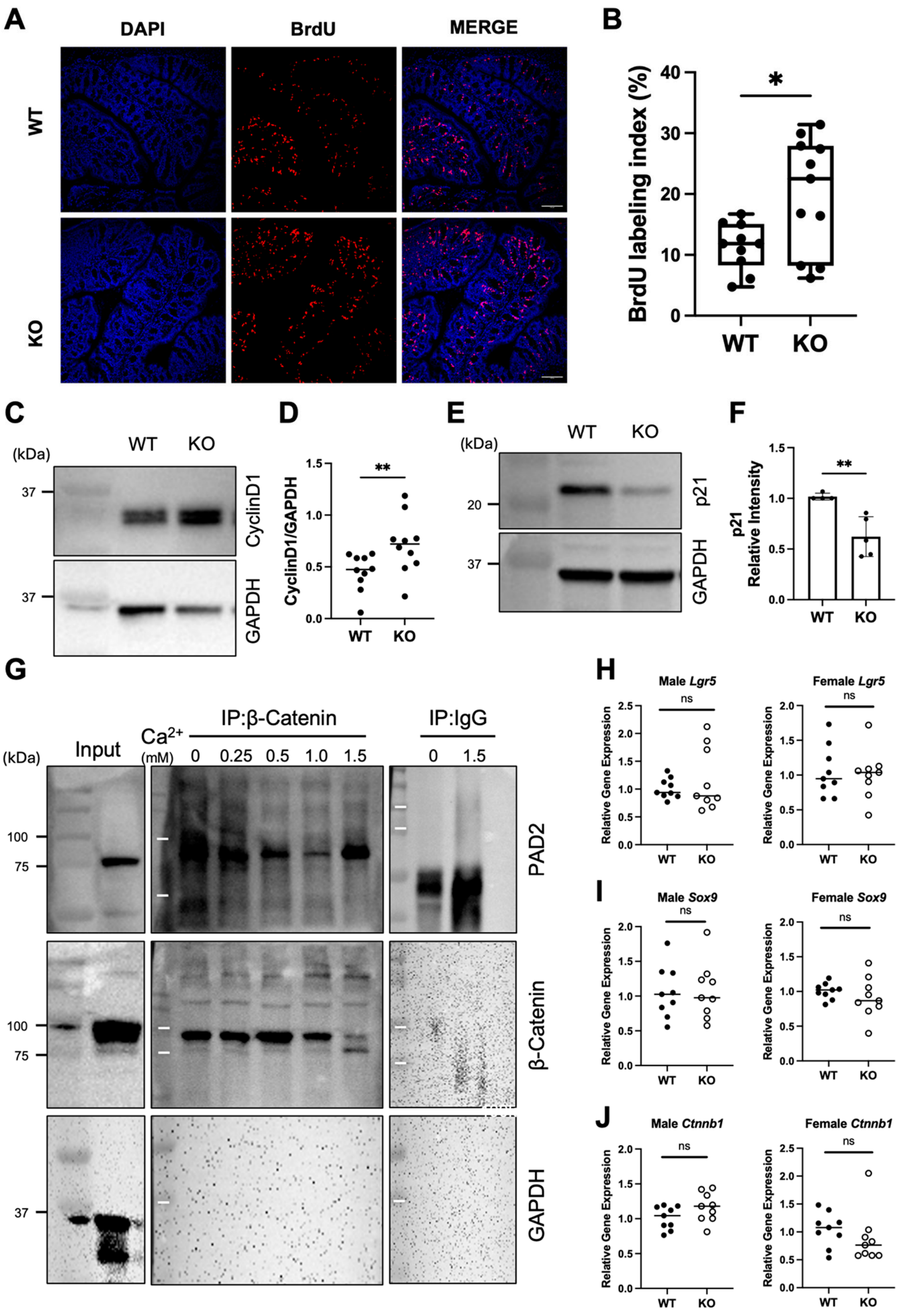

Figure S4. Additional characterization of epithelial proliferation, differentiation, and β-catenin regulation following PAD2 deficiency. (**A, B**) Representative immunofluorescence images of BrdU-positive nuclei (A) and BrdU labeling index (B) in female

WT and PAD2 KO colons collected 24 hours after BrdU administration. Scale bar = 100 μm. (**C, D**) Representative immunoblots (C) and densitometric quantification (D) of Cyclin D1 normalized to GAPDH in female WT and PAD2 KO proximal colon lysates (n = 10 mice per genotype). (**E, F**) Representative immunoblots (E) and densitometric quantification (F) of p21 normalized to GAPDH in female WT and PAD2 KO proximal colon lysates (n = 4 WT and 5 KO mice). (**G**) Representative immunoblots of PAD2 and β-catenin following β-catenin immunoprecipitation from proximal WT colon lysates. Samples include WT input lysate, PAD2-positive control, immunoprecipitated β-catenin following incubation with 0, 0.25, 0.5, 1.0, or 1.5 mM $CaCl_2$, and WT IgG immunoprecipitation controls as indicated. **(H)** Relative *Lgr5* mRNA expression in male (P = 0.7880) or female (P = 0.7050) WT and PAD2 KO colons (n = 9 mice per sex and genotype). **(I)** Relative *Sox9* mRNA expression in male (P = 0.6152) or female (P = 0.0884) WT and PAD2 KO colons (n = 9 mice per sex and genotype). **(J)** Relative *Ctnnb1* mRNA expression in male (P = 0.0679) or female (P = 0.2973) WT and PAD2 KO colons (n = 9 mice per sex and genotype). Data in B are presented as a box-and-whisker plot showing the median, interquartile range, and minimum-to-maximum values, with individual biological replicates shown. Data in D, F, and H-J are presented as mean ± SD, with individual biological replicates shown. BrdU labeling index, Cyclin D1 abundance, and p21 abundance were analyzed using two-sided Welch's t tests. Gene expression was analyzed using two-way ANOVA with genotype and qPCR run as factors, followed by Šídák-adjusted planned comparisons. * $P < .05$; ** P < .01; *** $P < .001$; **** P < .0001.
