# Supplementary material for: PAD2 Deficiency Reveals Sex-Divergent Colonic Homeostasis, β-Catenin Remodeling, and Antibiotic-Sensitive Phenotypes
Source: Biomolecules. 2026 Sep 30;16(10):1415. doi: 10.3390/biom16101415 (PMC13702572; doi:10.3390/biom16101415)
Supplement: Supplementary file 1 [file biomolecules-16-01415-s001.zip › Table S1_Terminal_Collection.pdf]

Animal	Sex	Genotype	Colon Length (mm)	Day at Collection
3918	M	WT	61	Day 14
3965	M	WT	62	Day 9
3993	M	WT	65	Day 10
5586	M	WT	63	Day 14
5714	M	WT	60	Day 12
6068	M	WT	55	Day 13
6069	M	WT	52	Day 14
6070	M	WT	52	Day 14
6071	M	WT	55	Day 14
6072	M	WT	59	Day 11
3967	M	KO	50	Day 10
3970	M	KO	51	Day 10
UT1	M	KO	54	Day 10
UT2	M	KO	61	Day 9
3975	M	KO	56	Day 10
5528	M	KO	67	Day 11
5529	M	KO	62	Day 9
5585	M	KO	60	Day 10
5594	M	KO	52	Day 10
5599	M	KO	54	Day 11
5478	F	WT	55	Day 10
5480	F	WT	55	Day 15
5417	F	WT	48	Day 16
5524	F	WT	51	Day 9
5525	F	WT	54	Day 9
5589	F	WT	52	Day 10
5590	F	WT	48	Day 16
5724	F	WT	66	Day 11
5725	F	WT	53	Day 10
5726	F	WT	48	Day 11
5326	F	KO	58	Day 16
5481	F	KO	56	Day 9
UT1	F	KO	71	Day 11
UT2	F	KO	55	Day 14
5451	F	KO	63	Day 10
5452	F	KO	62	Day 9
5453	F	KO	52	Day 11
5454	F	KO	58	Day 11
5455	F	KO	65	Day 10
5416	F	KO	48	Day 9

Table S1: Terminal Collection Day and Colon Length in the Longitudinal DSS Survival Cohort. Terminal collection timing varied among animals according to disease progression; therefore, colon-length measurements represent terminal outcomes rather than fixed-time comparisons
