# Supplementary material for: PAD2 Deficiency Reveals Sex-Divergent Colonic Homeostasis, β-Catenin Remodeling, and Antibiotic-Sensitive Phenotypes
Source: Biomolecules. 2026 Sep 30;16(10):1415. doi: 10.3390/biom16101415 (PMC13702572; doi:10.3390/biom16101415)
Supplement: Supplementary file 1 [file biomolecules-16-01415-s001.zip › Table_S2_Pathway_Abundance_Analysis.pdf]

A. Female mice (n = 4 WT/KO matched littermate pairs)								
**MetaCyc pathway**	**WT median abundance**	**PAD2 KO median abundance**	**Median paired difference (KO–WT)**	**Median KO/WT ratio**	**Rank-biserial effect size**	**Wilcoxon W**	**Raw P**	**BH-FDR (q)**
folate transformations III (E. coli)	522.998	247.523	-244.782	0.473	-1.000	0	0.1250	0.1838
glycolysis III (from glucose)	539.941	173.612	-333.066	0.322	-1.000	0	0.1250	0.1838
L-arginine biosynthesis I (via L-ornithine)	336.348	378.400	45.070	1.125	0.400	3	0.6250	0.6250
L-arginine biosynthesis II (acetyl cycle)	335.217	390.844	62.207	1.166	0.400	3	0.6250	0.6250
chorismate biosynthesis I	423.239	240.992	-121.087	0.569	-1.000	0	0.1250	0.1838
superpathway of branched chain amino acid biosynthesis	478.167	307.282	-137.663	0.643	-0.600	2	0.3750	0.4464
coenzyme A biosynthesis I (prokaryotic)	445.855	252.501	-189.853	0.566	-1.000	0	0.1250	0.1838
superpathway of coenzyme A biosynthesis III (mammals)	465.943	282.923	-177.231	0.607	-1.000	0	0.1250	0.1838
superpathway of aromatic amino acid biosynthesis	372.763	225.048	-98.856	0.604	-0.800	1	0.2500	0.3378
dTDP-beta-L-rhamnose biosynthesis	649.220	344.746	-270.073	0.531	-1.000	0	0.1250	0.1838
superpathway of fatty acid biosynthesis initiation (E. coli)	403.587	303.001	-75.599	0.751	-0.400	3	0.6250	0.6250
gluconeogenesis I	537.359	289.678	-260.812	0.539	-1.000	0	0.1250	0.1838
glycolysis I (from glucose 6-phosphate)	497.337	276.655	-218.805	0.556	-1.000	0	0.1250	0.1838

L-isoleucine biosynthesis I (from threonine)	714.315	590.416	-151.032	0.827	-0.400	3	0.6250	0.6250
methylerythritol phosphate pathway I	475.059	283.397	-168.671	0.597	-1.000	0	0.1250	0.1838
phosphopantothenate biosynthesis I	483.603	178.011	-311.275	0.368	-1.000	0	0.1250	0.1838
superpathway of coenzyme A biosynthesis I (bacteria)	433.425	207.801	-232.636	0.479	-1.000	0	0.1250	0.1838
peptidoglycan biosynthesis I (meso-diaminopimelate containing)	533.251	313.486	-176.019	0.588	-1.000	0	0.1250	0.1838
glycolysis IV	836.156	475.347	-325.255	0.568	-1.000	0	0.1250	0.1838
L-lysine biosynthesis III	483.856	237.119	-247.505	0.490	-1.000	0	0.1250	0.1838
folate transformations II (plants)	746.220	381.486	-357.862	0.511	-1.000	0	0.1250	0.1838
PWY-4242	419.484	250.122	-165.156	0.596	-1.000	0	0.1250	0.1838
L-lysine biosynthesis VI	490.815	327.962	-159.352	0.668	-0.800	1	0.2500	0.3378
L-isoleucine biosynthesis III	426.247	270.747	-121.973	0.635	-0.600	2	0.3750	0.4464
glycolysis II (from fructose 6-phosphate)	455.821	210.588	-245.233	0.462	-1.000	0	0.1250	0.1838
UMP biosynthesis I	777.264	451.870	-266.387	0.581	-1.000	0	0.1250	0.1838
inosine 5'-phosphate degradation	514.352	168.117	-271.418	0.327	-1.000	0	0.1250	0.1838
cis-vaccenate biosynthesis	419.721	251.463	-168.259	0.599	-1.000	0	0.1250	0.1838
5-aminoimidazole ribonucleotide biosynthesis I	461.536	345.491	-74.036	0.749	-0.600	2	0.3750	0.4464
5-aminoimidazole ribonucleotide biosynthesis II	627.992	396.309	-174.430	0.631	-1.000	0	0.1250	0.1838

inosine-5'-phosphate biosynthesis I	578.405	215.169	-319.456	0.372	-1.000	0	0.1250	0.1838
inosine-5'-phosphate biosynthesis II	544.719	194.973	-308.409	0.358	-1.000	0	0.1250	0.1838
superpathway of adenosine nucleotides de novo biosynthesis II	450.376	217.264	-254.437	0.482	-1.000	0	0.1250	0.1838
S-adenosyl-L-methionine salvage I	395.117	229.122	-150.026	0.580	-0.600	2	0.3750	0.4464
chorismate biosynthesis from 3-dehydroquinate	500.989	248.416	-206.910	0.496	-1.000	0	0.1250	0.1838
superpathway of 5-aminoimidazole ribonucleotide biosynthesis	627.992	396.309	-174.430	0.631	-1.000	0	0.1250	0.1838
peptidoglycan biosynthesis III (mycobacteria)	393.486	270.719	-128.453	0.688	-0.400	3	0.6250	0.6250
UDP-N-acetylmuramoyl-pentapeptide biosynthesis II (lysine-containing)	384.088	242.661	-145.278	0.632	-0.600	2	0.3750	0.4464
UDP-N-acetylmuramoyl-pentapeptide biosynthesis I (meso-diaminopimelate containing)	519.771	307.498	-154.713	0.592	-1.000	0	0.1250	0.1838
adenine and adenosine salvage III	577.062	381.680	-172.297	0.661	-0.800	1	0.2500	0.3378
queuosine biosynthesis I (de novo)	588.972	352.140	-250.995	0.598	-1.000	0	0.1250	0.1838
pyruvate fermentation to isobutanol (engineered)	714.315	590.416	-151.032	0.827	-0.400	3	0.6250	0.6250

adenosine ribonucleotides de novo biosynthesis	900.369	553.117	-285.226	0.614	-1.000	0	0.1250	0.1838
guanosine ribonucleotides de novo biosynthesis	567.288	319.481	-301.261	0.563	-1.000	0	0.1250	0.1838
superpathway of adenosine nucleotides de novo biosynthesis I	534.021	263.411	-303.368	0.493	-1.000	0	0.1250	0.1838
L-arginine biosynthesis IV (archaebacteria)	337.711	377.283	48.507	1.117	0.400	3	0.6250	0.6250
gondoate biosynthesis (anaerobic)	374.374	222.281	-145.264	0.594	-1.000	0	0.1250	0.1838
superpathway of purine nucleotides de novo biosynthesis I	428.628	218.629	-223.997	0.510	-1.000	0	0.1250	0.1838
tRNA charging	764.876	467.129	-299.453	0.611	-1.000	0	0.1250	0.1838
L-valine biosynthesis	714.315	590.416	-151.032	0.827	-0.400	3	0.6250	0.6250

B. Male mice (n = 5 WT/KO matched littermate pairs)								
**MetaCyc pathway**	**WT median abundance**	**PAD2 KO median abundance**	**Median paired difference (KO−WT)**	**Median KO/WT ratio**	**Rank-biserial effect size**	**Wilcoxon W**	**Raw P**	**BH-FDR (q)**
folate transformations III (E. coli)	220.252	333.713	15.176	1.515	0.333	5	0.6250	0.9448
glycolysis III (from glucose)	225.110	253.806	28.696	1.127	-0.200	6	0.8125	0.9448
L-arginine biosynthesis I (via L-ornithine)	173.866	173.183	-15.389	0.996	0.067	7	1.0000	1.0000
L-arginine biosynthesis II (acetyl cycle)	165.363	176.956	-33.138	1.070	0.067	7	1.0000	1.0000
chorismate biosynthesis I	168.669	185.368	26.832	1.099	0.200	6	0.8125	0.9448

superpathway of branched chain amino acid biosynthesis	186.879	228.983	-28.369	1.225	0.200	6	0.8125	0.9448
coenzyme A biosynthesis I (prokaryotic)	219.159	249.656	-32.444	1.139	0.200	6	0.8125	0.9448
superpathway of coenzyme A biosynthesis III (mammals)	223.235	281.263	-27.567	1.260	0.200	6	0.8125	0.9448
dTDP-beta-L-rhamnose biosynthesis	277.486	452.046	39.367	1.629	0.333	5	0.6250	0.9448
superpathway of fatty acid biosynthesis initiation (E. coli)	199.569	253.006	60.655	1.268	-0.067	7	1.0000	1.0000
gluconeogenesis I	285.011	227.894	-30.431	0.800	-0.333	5	0.6250	0.9448
glycolysis I (from glucose 6-phosphate)	240.772	194.705	-22.943	0.809	-0.333	5	0.6250	0.9448
L-histidine biosynthesis	227.266	281.501	17.244	1.239	0.333	5	0.6250	0.9448
L-isoleucine biosynthesis I (from threonine)	359.173	476.834	49.046	1.328	0.200	6	0.8125	0.9448
methylerythritol phosphate pathway I	207.664	305.929	-0.813	1.473	0.200	6	0.8125	0.9448
phosphopantothenate biosynthesis I	197.509	290.748	44.887	1.472	0.200	6	0.8125	0.9448
superpathway of coenzyme A biosynthesis I (bacteria)	202.388	251.358	-1.893	1.242	0.067	7	1.0000	1.0000
peptidoglycan biosynthesis I (meso-diaminopimelate containing)	235.456	279.547	18.879	1.187	0.333	5	0.6250	0.9448
glycolysis IV	415.288	493.827	44.984	1.189	0.333	5	0.6250	0.9448
L-lysine biosynthesis III	209.813	332.071	41.978	1.583	0.333	5	0.6250	0.9448

folate transformations II (plants)	345.966	491.268	69.610	1.420	0.333	5	0.6250	0.9448
PWY-4242	199.521	251.089	-11.220	1.258	0.200	6	0.8125	0.9448
L-lysine biosynthesis VI	203.009	333.621	45.186	1.643	0.200	6	0.8125	0.9448
L-isoleucine biosynthesis III	155.334	188.540	-21.522	1.214	0.200	6	0.8125	0.9448
glycolysis II (from fructose 6-phosphate)	196.033	158.582	-18.290	0.809	-0.333	5	0.6250	0.9448
UMP biosynthesis I	295.657	473.347	1.693	1.601	0.333	5	0.6250	0.9448
inosine 5'-phosphate degradation	204.606	309.361	12.960	1.512	0.467	4	0.4375	0.9448
cis-vaccenate biosynthesis	176.680	209.614	-2.648	1.186	-0.067	7	1.0000	1.0000
5-aminoimidazole ribonucleotide biosynthesis I	171.182	222.146	11.214	1.298	0.200	6	0.8125	0.9448
5-aminoimidazole ribonucleotide biosynthesis II	226.078	329.082	60.835	1.456	0.200	6	0.8125	0.9448
inosine-5'-phosphate biosynthesis I	242.523	384.407	57.516	1.585	0.467	4	0.4375	0.9448
inosine-5'-phosphate biosynthesis II	224.262	366.194	56.077	1.633	0.467	4	0.4375	0.9448
superpathway of guanosine nucleotides de novo biosynthesis II	194.885	252.469	64.375	1.295	0.200	6	0.8125	0.9448
superpathway of adenosine nucleotides de novo biosynthesis II	240.779	325.120	95.031	1.350	0.333	5	0.6250	0.9448
S-adenosyl-L-methionine salvage I	183.891	252.925	23.559	1.375	0.333	5	0.6250	0.9448
chorismate biosynthesis from	182.653	282.289	36.520	1.545	0.333	5	0.6250	0.9448

3-dehydroquinate								
superpathway of 5-aminoimidazole ribonucleotide biosynthesis	226.078	329.082	60.835	1.456	0.200	6	0.8125	0.9448
peptidoglycan biosynthesis III (mycobacteria)	170.953	202.264	-6.913	1.183	-0.067	7	1.0000	1.0000
UDP-N-acetylmuramoyl-pentapeptide biosynthesis I (meso-diaminopimelate containing)	223.709	261.254	16.829	1.168	0.333	5	0.6250	0.9448
adenine and adenosine salvage III	283.131	318.790	86.963	1.126	0.200	6	0.8125	0.9448
queuosine biosynthesis I (de novo)	266.285	467.783	38.206	1.757	0.333	5	0.6250	0.9448
pyruvate fermentation to isobutanol (engineered)	359.173	476.834	49.046	1.328	0.200	6	0.8125	0.9448
adenosine ribonucleotides de novo biosynthesis	420.916	550.667	57.767	1.308	0.333	5	0.6250	0.9448
guanosine ribonucleotides de novo biosynthesis	308.493	333.769	151.853	1.082	0.333	5	0.6250	0.9448
superpathway of guanosine nucleotides de novo biosynthesis I	214.558	274.260	71.722	1.278	0.200	6	0.8125	0.9448
superpathway of adenosine nucleotides de novo biosynthesis I	284.117	382.618	91.279	1.347	0.333	5	0.6250	0.9448
L-arginine biosynthesis IV (archaebacteria)	174.434	174.086	-13.904	0.998	0.067	7	1.0000	1.0000

superpathway of purine nucleotides de novo biosynthesis I	214.464	264.117	70.466	1.232	0.333	5	0.6250	0.9448
tRNA charging	354.495	446.524	39.826	1.260	0.333	5	0.6250	0.9448
L-valine biosynthesis	359.173	476.834	49.046	1.328	0.200	6	0.8125	0.9448

Table S2: Pathway Abundance Analysis. Abundance values are quantitative MetaCyc pathway-abundance outputs from the One Codex functional analysis workflow and represent predicted metagenomic functional potential rather than measured metabolic activity or metabolite production. Median paired difference is the median of within-pair PAD2 KO-WT abundance differences. Positive values indicate higher abundance in KO; negative values indicate lower abundance in KO. Rank-biserial effect size summarizes the paired direction and magnitude; positive values indicate higher abundance in KO and negative values indicate lower abundance in KO. No pathway met an FDR-adjusted significance threshold of $q < 0.05$ in either sex. Female minimum raw $P = 0.125$; male minimum raw $P = 0.4375$.
